# Temporal Trends and ICD-11-Mapped Patterns of Otology Research in Saudi Arabia, 1978–2024: A Scoping Review Using Negative Binomial Modelling

**DOI:** 10.3390/audiolres16030094

**Published:** 2026-06-22

**Authors:** Nawaf Khayal Alkhayal, Mohammed Sherif, Yousef Fares Shata, Leen Z. Alotaibi, Fayez A. Alhabib, Hesham Saleh Almofada

**Affiliations:** 1 College of Medicine, Alfaisal University, Riyadh 11533, Saudi Arabia; msherif@alfaisal.edu (M.S.); alhabib.fayez@gmail.com (F.A.A.);; 2 Otorhinolaryngology Head and Neck Surgery and Communication Sciences Department, King Faisal Specialist Hospital and Research Center, Riyadh 11211, Saudi Arabia

**Keywords:** scoping review, ear diseases, Saudi Arabia, ICD-11 classification, negative binomial regression

## Abstract

**Purpose:** This study aimed to map publication trends, topical focus, study designs, and institutional concentration in otology research in Saudi Arabia from 1978 to 2024 to deduce any topical, regional, institutional, or funding disparities in the field of otology in the country. **Methods:** We conducted a scoping review of studies on human ear diseases in Saudi Arabia, searching PubMed and the Cochrane Library from inception to 31 December 2024. Bibliometric characteristics were charted, topics were mapped to ICD-11 chapters, and temporal trends were modelled using negative binomial regression with a single data-driven breakpoint. **Results:** Of 2227 records identified, 510 studies were included. Annual output increased by 9.28% (95% CI 7.05–11.55). An inflection occurred around 2017, with slower growth before 2017 (7.2%/year, 95% CI 5.3–9.1) and faster growth from 2018 onward (23.9%/year, 95% CI 18.6–29.4). The institutional affiliation of first authors was concentrated in a small number of organizations, led by King Saud University. Observational studies predominated (441/510), whereas experimental studies were limited (16/510). ICD-11 mapping showed the greatest concentration in “Ear and mastoid” (189/510, 37.1%) and “Factors influencing health status or contact with health services” (179/510, 35.1%) chapters. Funding was reported in 75 studies. **Conclusions:** PubMed- and Cochrane-indexed otology and hearing health research output in Saudi Arabia has grown substantially, particularly since 2017, but remains concentrated by institution, region, study design, and topic. The dominance of cochlear implant and hearing impairment research, together with limited multicenter, experimental, vestibular, tinnitus, and rehabilitation-focused studies, identifies priorities for future audiology and neurotology research planning.

## 1. Introduction

Ear diseases involve a broad range of pathologies that can arise in the outer, middle, or inner ear and typically manifest as symptoms such as tinnitus, vertigo, pain, hearing loss, and discharge [1]. In Saudi Arabia, these conditions account for a significant proportion of otolaryngology-related visits—38.2% of all ENT presentations in the Qassim University outpatient cohort (2018–2020; n = 2596) [2]. At the global level, the Global Burden of Disease (GBD) 2019 analysis estimated that 1.57 billion people—approximately 1 in 5—experienced hearing loss in 2019, with projections reaching 2.45 billion by 2050 [3].

Despite these statistics, research on ear diseases in Saudi Arabia remains fragmented. For instance, several investigations have highlighted fundamental gaps in the literature, such as with respect to the efficacy of newborn hearing screening programs and complications in post-cochlear implant patients [4,5]. In addition, many studies have reported constraints related to single-centre recruitment and small sample sizes, limiting the generalizability of their findings [6,7]. These methodological limitations hinder the development of broad policy recommendations and clinical guidelines. Additionally, prior work has emphasized the fact that local data on the relationship between hearing loss and dementia are limited [6]. These examples illustrate the evident gap in the literature concerning local research.

In the broader international context, ear diseases have been the subject of a rapidly expanding body of research. Bibliometric analyses indicate that the literature on ear disorders is voluminous and diverse. For instance, more than 5700 tinnitus-related articles were published between 2001 and 2020, with the number of publications increasing dramatically since 2010 [8]. Research on hearing loss is even more extensive, accounting for more than 8000 publications between 1980 and 2023 [9,10]. Studies of chronic otitis media reveal that the most influential articles have more than 1000 citations, and journal-specific h-indices for hearing research often exceed 50, reflecting sustained citation impact [11]. This surge underscores the global recognition of ear diseases as a major public health issue and highlights the diversity of topics investigated, from genetics and pathophysiology to clinical interventions, quality-of-life research, and technological innovations such as artificial intelligence (AI) and novel imaging techniques [9].

Moreover, global output is concentrated in only a few research-intensive countries. The United States, Germany, the United Kingdom, China, and Japan jointly contribute the most ear-disease publications, with the U.S. alone accounting for nearly one-third of tinnitus research [8]; similar dominance is observed with respect to hearing-loss studies [9]. Specialized journals such as *Hearing Research*, *Ear and Hearing*, and *Otology & Neurotology* publish large volumes of otologic research and achieve high citation rates [8]. In contrast, Saudi Arabia’s contribution to the global ear disease literature remains modest, and it is not among the top-producing countries worldwide [8,9]. Furthermore, research on otology is often misaligned with actual audiovestibular symptom prevalence and seems to prefer hearing impairment over vestibular symptoms [12,13,14]. To discern and highlight possible deficits in otologic research in Saudi Arabia, we have conducted a scoping review of ear disease research along with topical mapping via the ICD-11.

## 2. Methods

### 2.1. Study Design and Reporting

The review followed Arksey and O’Malley’s framework with Joanna Briggs Institute’s (JBI) refinements and is reported in line with the Preferred Reporting Items for Systematic reviews and Meta-Analyses extension for Scoping Reviews (PRISMA-ScR) guidelines [15,16]. In line with the conventions for scoping reviews, we did not conduct a study-level risk-of-bias/critical appraisal of the included sources. A protocol was not registered; eligibility criteria and analysis plans were specified before screening began.

### 2.2. Information Sources and Search Strategy

A comprehensive literature search was performed on PubMed and the Cochrane Library from database inception to 31 December 2024 to avoid partial-year bias. Strategies combined title/abstract keywords and MeSH terms for ear diseases with terms for Saudi Arabia, using Boolean operators and truncation. The full, reproducible strategies (including any filters and limits) are presented in Table S1. All records were managed in Zotero (v6.0); duplicates were removed using Zotero’s native duplicate-detection workflow.

### 2.3. Eligibility (PCC Framework)

#### 2.3.1. Population

All otology-related populations and scholarly outputs were eligible except for animal studies.

#### 2.3.2. Concept

Otology-related research on prevalence/risk factors, diagnosis, management, outcomes, prevention, quality of life, health policy, or technologies (e.g., implants, audiology) across all study designs (observational, interventional, reviews (systematic reviews, literature reviews), qualitative, basic science, bibliometrics).

#### 2.3.3. Context

Studies conducted in Saudi Arabia or in collaboration with Saudi institutions; multi-country studies were eligible when Saudi data were reported separately or when a Saudi institution was a contributing site.

#### 2.3.4. Additional Criteria

Indexed in PubMed or the Cochrane Library. We excluded animal-only studies and non-Saudi settings without separable Saudi data. No language restrictions were applied during the search stage.

### 2.4. Selection of Sources of Evidence

In the first screening stage, two independent reviewers assessed the titles and abstracts of all retrieved records against the PCC-derived eligibility criteria. Citations not meeting the predefined eligibility criteria were excluded, while those with unclear relevance were retained for further review. Duplicate records, arising from overlapping database coverage, were identified and removed prior to screening. The second screening stage involved a full-text review of selected citations conducted by two independent reviewers. Reasons for exclusion at the full-text stage were recorded using PCC-linked categories (out of scope for population/concept/context or not Saudi context). Disagreements at either step were resolved by consensus with a third reviewer.

### 2.5. Data Charting Process and Data Items

We used a standardized, pilot-tested charting form to extract bibliographic details (authors, month/year, journal, and publication type), study characteristics (topic, region within Saudi Arabia, design, and first-author affiliations), and funding/conflict of interest statements. First-author affiliation was used as the unit for institutional and regional attribution because PubMed consistently indexes the first author’s affiliation across the full study period, whereas complete affiliation data for all contributing authors are inconsistently available for older records; fractional and full-author counting were therefore not applied to avoid systematic missingness across the 1978–2024 series. Data were primarily extracted from PubMed records (author panel for names/affiliations; record fields for publication type, journal, and date; and indexed funding/COI statements). To harmonize the topical classification, two reviewers mapped each record to the International Classification of Diseases (ICD)-11 (chapter → block → category) using the official ICD browser and coding tool to ensure uniformity (Supplementary Methods S1). One primary reviewer charted all records, and a second reviewer independently verified charted fields by reference to the PubMed record; discrepancies were resolved by consensus or a third reviewer. Agreement between the primary reviewer’s initial ICD-11 assignments and the final reconciled assignments (following independent verification and third-reviewer adjudication) was quantified using Cohen’s kappa at the chapter, block, and category levels, with 95% confidence intervals. Data were managed in Microsoft Excel (Microsoft 365) with routine backups. Institutional names were harmonized using predefined affiliation-cleaning rules to account for institutional renaming and restructuring (Supplementary Methods S2).

### 2.6. Synthesis of Results

Given the heterogeneity of topics and designs, we used descriptive synthesis. We narratively summarized bibliometric variables (year/month, journal, affiliations) and study characteristics (design, topic, funding, conflicts). The mapped evidence is presented in the figures and tables.

### 2.7. Statistical Analysis

#### 2.7.1. Overdispersion Assessment and Model Selection

We initially fit a Poisson generalized linear model (GLM, log link) to assess annual counts [17]. The Pearson dispersion statistic ($\phi =\frac{\chi_{Pearson}^{2}}{{df}_{residual}}$) substantially exceeded unity (ϕ = 2.97), which indicated significant overdispersion and invalidating the Poisson assumption of variance equal to the mean [17,18]. Consequently, we employed negative binomial regression (NB2 parameterization), which accommodates overdispersion through an additional dispersion parameter (α) with variance [18,19]:


$$Var\left(Y_{i}|x_{i}\right)=\;\mu_{i}+\;\alpha \mu_{i}^{2}$$

#### 2.7.2. Primary Trend Analysis

The NB2 model was specified as follows:

$$\mu ᵢ\;=\;E[Yᵢ\;|\;xᵢ]\;=\;exp\{\beta_{0}\;+\;\beta_{1}\;({year}_{i}\;-\;min\{year\})\}$$

where $\mu_{i}$ is the expected count in year i, and years were centred at the first observed year to make the intercept interpretable and to reduce potential collinearity. We estimated the *APC* as follows:

$$APC\;=\;100\;\times \;(exp(\beta_{1})\;-\;1)$$

which represents the year-on-year change in expected counts [17,19]. For primary inference, we used Newey–West HAC standard errors with maxlags = 3.

#### 2.7.3. Change-Point (Hinge) Scan and Selection

To allow for a data-driven change in slope in an unknown year, we used a continuous hinge parameterization with ${hinge}_{i}=\max\left(0,\;{year}_{i}-\;bp\right)$ and fit following segmented/hinge regression principles [20].


$$\log\left(\mu_{i}\right)=\beta_{0}+\beta_{1}year_{c,i}+\beta_{2}{hinge}_{i}$$

Candidate break years were restricted by a symmetric 15% trimming of the observed time points so that each segment contained at least h = ⌊0.15T⌋ observed years (with *T* the series length) [21,22]. For each candidate bp ∈ [ min(year) + h,\; max(year) − h ], we fit a discrete NB2 model (re-estimating α) and recorded Akaike’s information criterion (*AIC*); the breakpoint was chosen by reference to the minimum *AIC*, and improvements were summarized as Δ*AIC* relative to the single-slope NB2 model [23,24]. If *AIC* ties occurred, the earliest *bp* in the grid was retained.

#### 2.7.4. Inference for the Selected Breakpoint

For the chosen breakpoint, we fit the piecewise NB2 model by maximum likelihood (estimating *α* jointly with the regression coefficients) and then computed Newey–West HAC standard errors (maxlags = 1) for inference [25,26]. This choice was guided by Pearson’s residual autocorrelation at lag 1 exceeding the ±2/√n band [27]. Pre- and postbreak APCs were derived from $\beta_{1}$ and $\beta_{1}+\;\beta_{2}$, respectively; the postbreak APC confidence interval was determined using the delta method [28] with

$$Var\left(\beta_{1}+\beta_{2}\right)=Var\left(\beta_{1}\right)+Var\left(\beta_{2}\right)+2\cdot Cov\left(\beta_{1},\;\beta_{2}\right)$$

(HAC alters standard errors and CIs only; coefficients and AIC are unchanged).

### 2.8. Model Validation

We assessed estimate stability using leave-one-year-out (LOYO) cross-validation, sequentially omitting each year and refitting the NB2 model with the remaining 46 observations while retaining the global centering origin (year_c = year − min(year)) (Table S4). This approach provides an internal validation check that estimates are not unduly influenced by any single year.

All analyses were conducted in Python 3.9.6 using statsmodels 0.14.5, numpy 2.0.2, and pandas 2.3.3.

### 2.9. Seasonality of Publication Month

We aggregated studies with a known publication month into 12 bins. We tested the null of no seasonality using Pearson χ^2^ goodness-of-fit with 11 df under (i) equal frequency (uniform) and (ii) frequency proportional to days per month. Statistical significance was assessed at a two-sided significance level of 0.05. Records without a reported publication month were excluded from the seasonality analyses but retained for all the other syntheses.

### 2.10. Topical Diversity (ICD-11)

Each record was assigned to a primary ICD-11 chapter. For the diversity/top-k calculations, we used a hybrid ICD-11 *focus category* variable: the two most frequent chapters (Ear and mastoid (Ch.10)and Factors influencing health status or contact with health services (Ch.24)) were subdivided using ICD-11 directory groupings (Block and, for Ch.24, Categories), whereas all other topics were retained at the chapter level. We computed the following: Shannon entropy $H^{\prime }=\;-\sum p_{i}\ln p_{i}$; Hill number D1 =expH′; Simpson concentration $\lambda \;=\;\sum p_{i}^{2}$; Hill number D2 =1λ; and Pielou evenness $J^{\prime }=\frac{H^{\prime }}{\ln S}$. Among these, *S* is the number of non-zero categories. We also report the top-k shares (cumulative proportion for k = 2, 3, and 5). Although extension codes are modifiers rather than diseases, we retained them as a standalone chapter. This approach is in line with our aim of capturing the *research landscape* rather than performing strict clinical diagnostic coding. The primary topic of research for each article was determined by each individual reviewer and was then searched for in the ICD-11 browsing tool to elicit the directory leading to the most appropriate diagnostic code. The studies were then assigned the chapter containing their disease code as well as a “Block” attribute that contained the parent directory of the code. For articles that are within chapter 24, a further subdivision “Category” was attributed to the studies to allow clearer differentiation of studies on analysis; this allocation addresses the directory directly containing the code. The subcategories within “Category” included the following: “Presence of device, implants or grafts” which was assigned studies that specifically contained populations with cochlear implants, discussed the effect, design, or function of cochlear implants or studied populations undergoing cochlear implantation and “Contact with health services for specific surgical interventions”, which, on the other hand, was allotted studies that addressed otological surgical interventions as well as analysis of surgical techniques outside of cochlear implantation. Furthermore, “Contact with health services for purposes of examination or investigation” was a subcategory attributed to studies addressing diagnostic or screening otological investigations, and “Contact with health services for nonsurgical interventions not involving devices” included studies that assessed or studied other non-surgical otological procedures or interventions outside of cochlear implantation.

### 2.11. Journal Quality (Scimago SJR and Quartiles)

Journal quality was evaluated using SJR scores and quartile classifications, with each article matched to the SJR value reported for its year of publication rather than a single contemporary value. Mean SJR values and the share of Q1 journals were calculated separately for the 1978–2017 and 2018–2024 periods. The change in Q1 share was expressed as both an absolute difference (percentage points) and a relative percentage increase between the two periods. Percent change was reported descriptively; statistical testing was not applied since SJR values represent population-level bibliometric indicators. Journals not indexed in Scimago were excluded from quartile-based calculations but retained for descriptive totals; for years before 1999, the 1999 SJR values were used as proxies given the absence of earlier data [29].

### 2.12. Global Comparator Construction and Analysis

To contextualize the national trend against the broader literature, we constructed a global comparator in PubMed using the ear- and otology-specific title/abstract, keyword, and MeSH terms from the primary search, with Saudi-affiliated records excluded; the full comparator query is provided in Table S8. Yearly publication counts were retrieved by publication date for 1978–2024. These counts are unscreened query hits rather than studies passing the eligibility screen applied to the Saudi corpus and are therefore used only to characterize the growth rate of the global literature, not its absolute volume. The series was analysed with the same negative binomial (NB2, log-link) model and breakpoint search used in the primary analysis; for the comparator, we report the overall (single-slope) average annual growth rate and additionally tested whether a change in slope at 2017 improved the global fit. Full specifications and outputs are reported in Table S8. The Cochrane Library was not separately queried for the comparator, which is descriptive and is intended only to situate the Saudi trajectory relative to global activity rather than to support causal inference.

## 3. Results

### 3.1. Study Selection

**Figure 1:**
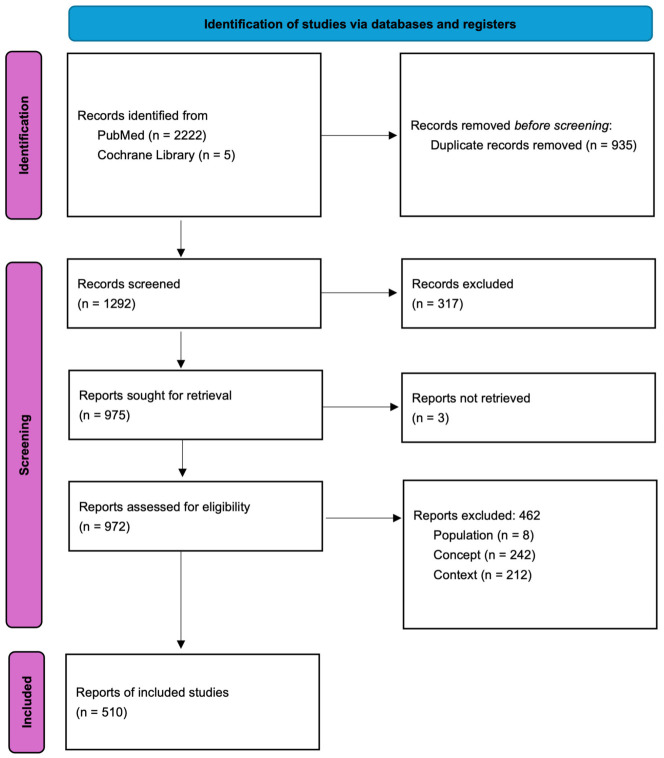
PRISMA-ScR flow diagram of identification, screening, eligibility, and inclusion (1978–2024), with exclusions summarized by PCC.

The search identified 2222 PubMed and 5 Cochrane records. After 935 duplicates were removed, 1292 records underwent title/abstract screening, and 317 records were excluded with 3 not retrieved. The remaining 972 full texts were assessed, and 462 were excluded because they did not meet the eligibility criteria. In total, 510 studies were included (Figure 1). Of the 462 full texts excluded, the reasons per PCC were as follows: Concept—not ear-/otology-focused (n = 242); Population—not disease-/human-focused (n = 8); and Context—not a Saudi-affiliated publication (n = 212).

### 3.2. Publication Trends by Year

**Figure 2:**
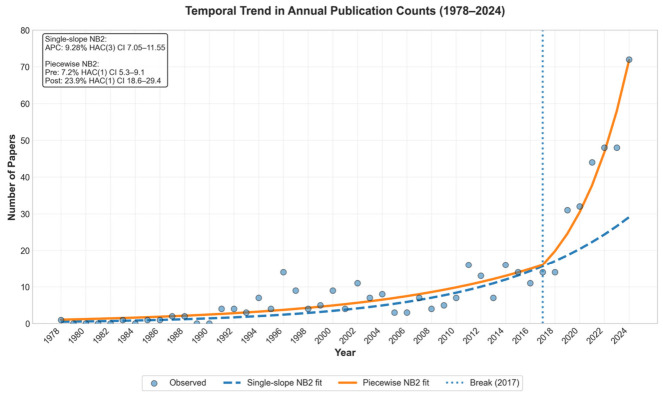
Publication trends in Saudi otology research (1978–2024). The dashed blue line shows the single-slope NB2 fit; the solid orange line shows the piecewise NB2 fit. The vertical dotted line marks the data-driven breakpoint in 2017.

Annual counts increased over time, progressing from minimal output in the 1980s–1990s (typically 1–5 papers/year) to reach higher volumes beginning in 2019 (2019: 31; 2020: 32; 2021: 44; 2022: 48; 2023: 48; 2024: 72) (Figure 2).

Over the 47-year study period (1978–2024, total n = 510), we observed a consistent upwards trend in annual publication counts. The negative binomial regression estimated an annual percentage change of 9.28% (Newey–West HAC (3), 95% CI: 7.05–11.55%) (relative risk per year = 1.093) (Table S3 and Figure S2).

Leave-one-year-out cross-validation confirmed estimate stability, in which context the APCs ranged from 8.91% to 9.79% (mean = 9.28%) across all 47 iterations with zero-convergence failures (Table S4 and Figure S3). Model diagnostics revealed an estimated dispersion parameter (α = 0.175), confirming substantial overdispersion relative to the Poisson model (φ = 2.97).

#### 3.2.1. Single-Break (Hinge) Analysis

A hinge NB2 with a breakpoint in 2017 improved the fit (AIC = 243.6 vs. 250.6; ΔAIC = −7.0). The coefficients implied that *APC* pre-2017 ≈ 7.2%/year ($\beta_{1}\approx \;$ 0.0695) and that *APC* post-2017≈ 23.9%/year ($\beta_{1}+\;\beta_{2}$ ≈ 0.214). The dispersion of the hinge model was smaller (α ≈ 0.046).

#### 3.2.2. Structural Break Analysis

Data-driven breakpoint scanning revealed 2017 as the optimal change point, with the piecewise model providing a substantially improved fit (AIC = 243.6) compared with the linear model (AIC = 250.6; ΔAIC = −7.0; Figure 2). The ΔAIC ≤ 2 confidence set, which was computed from the piecewise scan, included 2015–2017, thus indicating moderate uncertainty in precise breakpoint timing but clear evidence of a change around 2017 (Table S5 and Figure S4).

The piecewise model revealed notably different growth rates in the pre- and post-2017 periods, detailed as follows.

- Pre-2017 (1978–2017, 40 years): Annual increase of 7.18% (Newey–West HAC (1), 95% CI: 5.31–9.09%)
- Post-2017 (2018–2024, 7 years): Annual increase of 23.92% (Newey–West HAC (1), 95% CI: 18.63–29.44%)
- Acceleration: The slope change at the turn of 2017 corresponded to a positive hinge coefficient (β = 0.145, SE = 0.028) and improved the descriptive fit of the model, consistent with an acceleration in output around 2017 rather than a single uniform long-term trend.

The post-2017 growth rate represents an approximately threefold acceleration relative to the pre-2017 period. The piecewise model also exhibited a lower overdispersion parameter (α = 0.046 vs. 0.175 in the linear model), thus suggesting better capture of the underlying data-generating process.

Additional model diagnostics and coefficient estimates are provided in the Tables S6A,B and S7, with residual plots shown in Figures S5 and S6.

### 3.3. Publication Timing by Month (Seasonality)

The monthly counts totaled 498 and ranged from 31 (May) to 58 (October) (Table 1). Twelve records lacked month metadata and were excluded from the seasonality tests. The distributions did not differ from uniform expectations (χ^2^ = 13.81; df = 11; *p* = 0.244) or from month length-adjusted expectations (χ^2^ = 12.65; df = 11; *p* = 0.317).

### 3.4. First-Author Organization

Research output was concentrated in only a few institutions: King Saud University (n = 179/510, 35.10%), King Faisal Specialist Hospital and Research Centre (34/510, 6.67%), King Abdulaziz University (31/510, 6.08%), Imam Abdulrahman Bin Faisal University (24/510, 4.71%), King Khalid University (19/510, 3.73%), King Saud bin Abdulaziz University for Health Sciences (18/510, 3.53%), King Abdulaziz Medical City (15, 2.94%), and Prince Sultan Military Medical City (15/510, 2.94%). Numerous additional organizations contributed 1–10 papers each.

### 3.5. Regions

**Figure 3:**
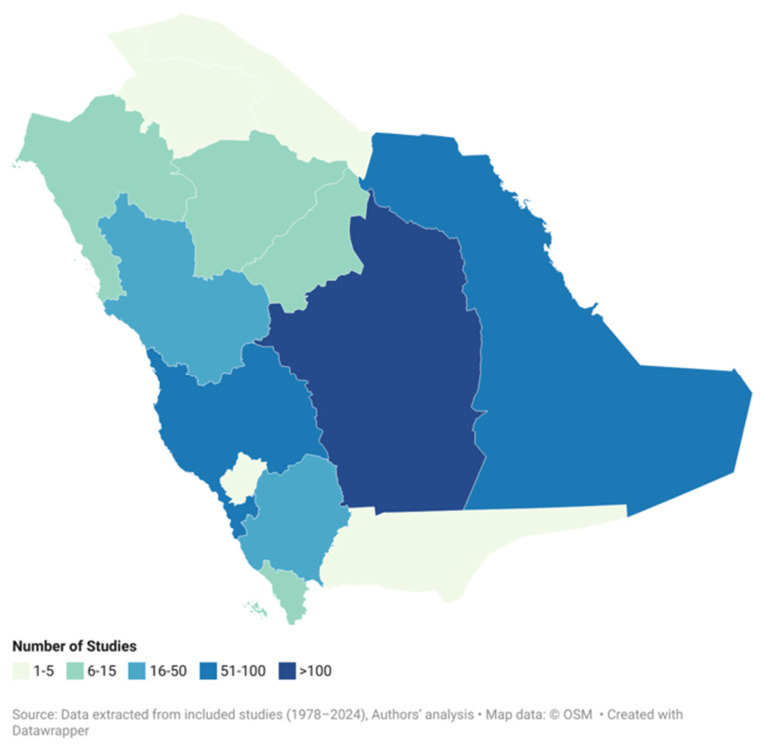
Regional distribution of otology studies in Saudi Arabia, 1978–2024: counts and percentages of studies by first-author affiliation region. Darker shading indicates higher counts, with Riyadh as the dominant hub (>300 studies), followed by Makkah and the Eastern Province (50–100), mid-range activity in Asir (16–30), and sparse output (1–15) across the remaining regions.

Regionally, the research output was highly concentrated in Riyadh (303/510; 59.41%), with secondary hubs in Makkah (68/510; 13.33%) and the Eastern Province (58/510; 11.37%). Mid-tier contributions were observed from Asir (29/510; 5.69%) and Madinah (16/510; 3.14%), followed by Qassim (11/510; 2.16%). The remaining regions each accounted for ≤1% of the papers: Tabuk (5/510; 0.98%), Jizan (5/510; 0.98%), Hail (5/510; 0.98%), Al Jawf (3/510; 0.59%), Al Bahah (3/510; 0.59%), Najran (2/510; 0.39%), and Northern Borders (2/510; 0.39%). Region counts reflect first-author affiliation. Multiaffiliation papers were assigned by reference to the first author (Figure 3). This pattern highlights a Riyadh-centric landscape with relatively little but notable activity along the Western and Eastern corridors.

### 3.6. Journals

Outputs appeared across a wide set of journals (Table 2). The leading outlets included *Saudi Medical Journal* (44/510), *International Journal of Pediatric Otorhinolaryngology* (36/510), *European Archives of Oto-Rhino-Laryngology* (24/510), *Cureus* (22), *Annals of Saudi Medicine* (22/510), *Journal of Laryngology & Otology* (19/510), and *Ear*, *Nose & Throat Journal* (15/510). Other journals included *Otology & Neurotology* (10/510), *Scientific Reports* (7/510), *Neurosciences* (Riyadh) (7/510), and *Laryngoscope* (7/510). The mean Scimago Journal Rank (SJR) increased modestly from 0.615 (1978–2017) to 0.638 (2018–2024), corresponding to a 3.68% increase in average journal impact. The share of publications in Q1 journals rose from 24.53% to 28.63%, representing a +4.10 percentage-point (16.71% relative) increase in high-impact outputs.

### 3.7. Publication Types

Among the 510 studies, 441 (86.47%) were observational (including 104 case reports), 49 (9.61%) were systematic reviews/meta-analyses, 16 (3.14%) were experimental, and 4 (0.78%) were other (2 consensus guideline and 2 technical innovation/feasibility reports).

### 3.8. Study Type by Journal

**Figure 4:**
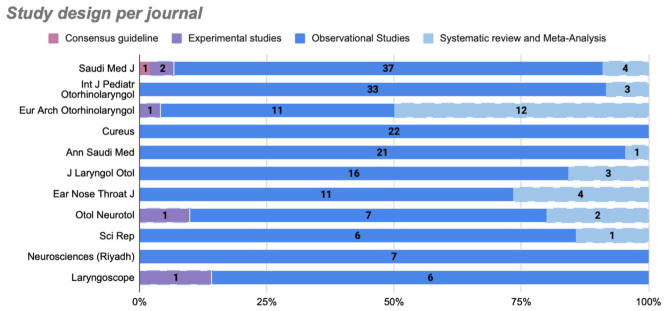
Cross-tabulation of study design by journal for the top outlets listed in Table 2 (counts and row percentages).

Studies published in *Saudi Medical Journal* (n = 44) and *International Journal of Pediatric Otorhinolaryngology* (n = 36) were predominantly observational (37 and 33), with 4 and 3 systematic reviews/meta-analyses, respectively, 2 experimental studies, and 1 consensus guideline for *SMJ*. *European Archives of Oto-Rhino-Laryngology* (n = 24) included 11 observational studies, 12 systematic reviews/meta-analyses, and 1 experimental study. *Cureus* (n = 22) was exclusively observational. *Annals of Saudi Medicine* (n = 22) and *Journal of Laryngology & Otology* (n = 19) primarily featured observational designs (n = 21 and n = 16), with minimal other types. Experimental studies were rare overall, appearing in only fifteen journals—*European Archives of Oto-Rhino-Laryngology*, *Otology & Neurotology*, *Saudi Medical Journal*, *PLoS One*, *Life*, *Laterality*, *Assistive technology*, *American Annals of the Deaf*, *Anaesthesia and Intensive Care*, *Frontiers in Public Health*, *Canadian Journal of Anaesthesia*, *Clinical and Experimental Otorhinolaryngology*, *International Immunopharmacology*, *J Deaf Stud Deaf Educ* and *Laryngoscope*—with one study each, with the exception of two for *SMJ* (Figure 4).

### 3.9. Topical Mapping (ICD-11)

**Figure 5:**
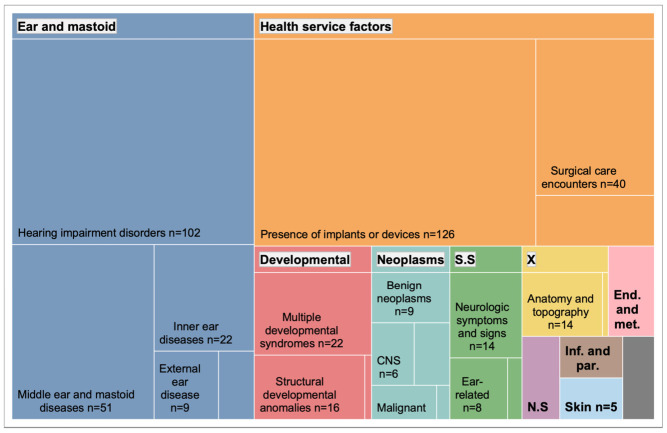
Treemap of included studies by ICD-11 chapter and block; low-frequency blocks (n < 10) grouped and unlabeled. Abbreviations: CNS, central nervous system; End met, endocrine, nutritional or metabolic diseases; Inf par, certain infectious or parasitic diseases; NS, diseases of the nervous system; SS, symptoms, signs or clinical findings, not elsewhere classified; X, Extension codes.

All 510 records were mapped across 16 ICD-11 chapters and blocks (Figure 5). Two chapters dominated: Ear and mastoid (Ch.10) (189/510; 37.06%) and Factors influencing health status or contact with health services (Ch.24) (179/510; 35.10%). Additional contributions included Developmental anomalies (Ch.20) (39/510; 7.65%), Neoplasms (Ch.2) (26/510; 5.10%), Symptoms, signs or clinical findings not elsewhere classified (Ch.21) (24/510; 4.71%), Extension Codes (Ch.X) (15/510; 2.94%), Endocrine, Nutritional or Metabolic Diseases (Ch.5) (8/510; 1.57%), Diseases of the nervous system (Ch.8) (6/510; 1.18%), Diseases of the musculoskeletal system or connective tissue (Ch.15) (5/510; 0.98%), Diseases of the skin (Ch.14) (5/510; 0.98%), Certain infectious or parasitic diseases (Ch.1) (5/510; 0.98%), Supplementary section for functioning assessment (Ch.V) (2/510; 0.39%), Injury, poisoning or certain other consequences of external causes (Ch.22) (2/510; 0.39%), Diseases of the circulatory system (Ch.11) (2/510; 0.39%), and Diseases of the visual system (Ch.9) (2/510; 0.39%). Only one article was concerned with Diseases of the immune system (Ch.4) (0.2%).

Agreement between the initial and final reconciled ICD-11 assignments was almost perfect at the chapter level (kappa = 0.93, 95% CI 0.90–0.95; 483/510 records concordant), block level (kappa = 0.92, 95% CI 0.89–0.94), and category level (kappa = 0.88, 95% CI 0.85–0.91). Chapter-level discrepancies (27/510) were concentrated at the boundary between active ear pathology (Ch.10) and device-related contact with health services (Ch.24). As this compared the primary reviewer’s initial coding with the reconciled coding rather than two independent blinded codings, these values index the stability of the initial assignments through verification and adjudication rather than fully blinded dual coding.

### 3.10. Diversity Metrics and Commonly Iterated Blocks (Bootstrap 95% CIs)

Across the hybrid ICD-11 focus categories (S = 24), topical diversity was moderate: Shannon *H*′ = 2.409 (2.289–2.478) with effective number ^1D = exp(*H*′) = 11.122 (9.866–11.920). However, concentration remained substantial (Simpson concentration *λ* = 0.132 (0.119–0.150), with effective number ^2D = 1/λ = 7.569 (6.647–8.416); evenness J′ = H′/ln S = 0.758). The most frequently iterated categories were *Presence of devices, implants, or grafts (Ch.24)* (126/510; 24.7%; largely cochlear implant work) and *Disorders with hearing impairment (Ch.10)* (102/510; 20.0%), followed by *Diseases of the middle ear or mastoid (Ch.10)* (51/510; 10.0%), *Contact with health services for specific surgical interventions (Ch.24)* (40/510; 7.8%), and *Developmental anomalies* (39/510; 7.6%). Collectively, the top two categories accounted for 44.7% of publications, the top three for 54.7%, and the top five for 70.2% (Table S2 and Figure S1).

### 3.11. Funding, Multicentre Studies, and Collaboration

Funding was reported in 75 studies, whereas 414 studies reported no funding, and 21 studies were unclear in this regard. Among the studies whose funding status was known (n = 489), 15.34% were funded, and 84.66% were non-funded. Seven multicenter studies were identified (6 observational), of which four were initiated by King Saud University. Among studies with disclosures, funding sources were dominated by universities (~50.67%) and government (~37.33%), with smaller contributions from industry (~5.33%), international sources (~9.33%), and NGOs (~1.33%) with some overlap between funding sources. Counts of funded studies increased (29/206 → 46/283) between the pre- and post-2018 periods. MED-EL involvement was noted in 27 studies (23 on cochlear implantation), and conflicts of interest were reported in 3/27. Conflict of interest disclosure practices are heterogeneous, and industry involvement may be underreported.

## 4. Discussion

This study documents a notable and sustained increase in the otology research productivity of Saudi Arabia over the past decade, with a clear post-2017 inflection point. Across the indicators we analyzed, outputs accelerated after 2017 in a manner consistent with an acceleration in output rather than year-to-year fluctuation alone. In practical terms, the Saudi otology health research system moved from a low-growth baseline to a more robust trajectory: overall productivity increased by 9.28% per year on average between 1978 and 2024, and segmented trend analyses indicate a modeled change in slope around 2017, with the annual growth rate increasing from 7.2% per year before 2018 to 23.9% per year thereafter. High-impact outputs increased modestly, with a 3.68% rise in mean SJR and a 16.71% relative (+4.10 percentage-point) increase in Q1 publications, which is consistent with an impact-oriented shift after 2017. This growth coincides with broader clinical and industrial priorities under the National Industry Strategy and the expansion of cochlear-implant centers nationwide [30]. This shift has also been accompanied by increasing multicenter collaboration and methodological maturation, considering the rising experimental and systematic review output since 2017. These findings suggest that national research reforms have coincided with measurable changes at the frontier of production.

### 4.1. Global Comparator

Using the same MeSH strategy, study window, and modelling approach but excluding Saudi affiliations, the global otology corpus showed a modest and steady increase of about 3.5% per year (95% CI 3.4–3.7%) (Table S8). This estimate is provided to contextualize the national trend rather than to imply causation. The Saudi trajectory remained substantially steeper and showed a clear post-2017 acceleration. Applying the same breakpoint search to the global series, a change in slope at the turn of 2017 did not improve the global fit, and the only supported inflection in the global series occurred earlier, around the mid-1990s; the marked post-2017 acceleration therefore appears specific to the Saudi corpus rather than a reflection of global publishing trends.

### 4.2. Institutional Concentration

Output remains anchored in a small set of institutions—KSU (35.10%), KFSH&RC (6.67%), and KAU (6.08%)—with the top five accounting for 56.27% of national publications. KSU’s share is congruent with the KSU 2030 Updated Strategic Plan, which links KPIs to indexed publications and patents and operationalizes them via priority domains, grant reorganization, research chairs, and interdisciplinary incentives; expansion of Riyadh Techno Valley and the Medical City further supports sustained output [31]. Furthermore, King Saud University’s extensive tenure and prestige as the first fully established university in Saudi Arabia may have contributed to its dominance in research output [32,33,34,35].

### 4.3. Geography

Production is geographically centralized in Riyadh 59.41% (303), Makkah (68), and the Eastern Province (58); the Southern and Northern regions each had <10 papers. This mirrors the distribution of tertiary hospitals, research centers, and postgraduate programs, suggesting capacity rather than topic preference as the primary driver. Under the Health Sector Transformation Program’s (HSTP) health cluster model, regional research development is prioritized; subsequent waves may show convergence toward greater geographic parity [30].

### 4.4. Thematic Structure and Diversification

Research in Saudi Arabia is concentrated in hearing loss and cochlear implantation, dominated by the topics ‘Ear and mastoid’ 37.06% and ‘Factors influencing health status’ 35.10%; few studies addressed vestibular disorders or tinnitus, and very few skull-base tumor papers were identified. Within our mapping of the ICD-11 Codes, 24.7% (126/510) of studies were device-based and were largely cochlear implant work. These studies were mapped to Chapter 24 (‘Presence of devices, implants or grafts’) rather than Chapter 10 because their primary ICD-11 codes describe the presence of and contact with health services for an implanted device rather than an active ear pathology; this is consistent with our stated aim of characterizing the research landscape rather than assigning strict clinical diagnoses. Although cochlear implantation ultimately treats sensorineural hearing loss, this literature centers on surgery, programming, and outcomes rather than hearing loss epidemiology, supporting its treatment as a distinct cluster. When the two dominant chapters were subdivided into focus categories, these device-related studies (24.7%) slightly exceeded those on disorders with hearing impairment (20.0%, 102/510), so combining them would have masked cochlear implantation as the single largest specific research focus. The scarcity of studies on vestibular disorders and tinnitus follow a global trend in preferential research on hearing impairment [12,13]. Moreover, the dominance of implant-related research likely reflects clinical volume plus a growing device ecosystem, rather than publication bias alone. Consistent with this, cochlear implant centers in Saudi Arabia have expanded markedly over the past six years, increasing from five centers in 2018 to seventeen by 2024, according to the Saudi Ministry of Health [36]. The more pronounced growth observed in clinical otology and hearing restoration studies, health services and outcomes research, and device-based innovation work (largely cochlear implantation) likely reflects alignment with national priorities (e.g., non-communicable diseases, digital health, and health services research) and the broader expansion of Saudi Arabia’s research and innovation ecosystem, including increased R&D financing, digital health capacity, and institutional research infrastructure [30,37,38,39,40].

### 4.5. Funding Signals and Timing

Funding visibility was limited across the included Saudi otology literature, with funding reported in only 75 of 489 studies with known funding status. Although the absolute number of funded studies increased from 29 before 2018 to 46 during 2018–2024, the proportion of funded studies increased only modestly, from 14.1% to 16.3%. These findings are therefore better interpreted as evidence of persistently limited funding visibility rather than a major post-2017 transformation in study-level funding. This interpretation is consistent with bibliometric literature showing that funding acknowledgments are useful but incomplete indicators of research support, because disclosure and indexing practices vary across journals, databases, document types, and time [41,42].

The predominance of university and government funders among studies with disclosed funding is consistent with the broader Saudi R&D profile, in which government accounted for 50% of R&D expenditure and the education sector accounted for 83% of R&D workers in 2021 [43]. The concentration of visibly funded work in cochlear implantation and hearing restoration topics may reflect the stronger institutional embedding of screening, implantation, and rehabilitation pathways rather than industry-driven sponsorship [36,44,45]. Although the post-2017 rise in otology output coincided with wider health-sector and research-system reforms, including HSTP, RDIA, and Saudi NIH activity, this study cannot establish whether those reforms caused the observed publication growth or changes in funding visibility [30,37,46,47].

### 4.6. Study Design Variety and Multicentre Capacity

Observational designs dominated (86.47%); experimental work was limited (3.14%) but increased after 2017, peaking in 2023–2024, and systematic reviews rose in parallel, suggesting methodological maturation. Only seven multicenter studies were identified. Given typical 2–5-year lead times from the HSTP launch to multicenter publication, counts likely lag policy initiatives. The Saudi NIH aims to unify authorities/centers and direct funds towards implementation milestones, which may improve multicenter capacity over time [46]. The preference for conducting observational studies may be associated with their relatively simpler logistics and accessibility [48]. On the other hand, there are many reported barriers slowing down the production of experimental work, mainly inadequacies in structure, funding, and resources [49].

### 4.7. Limitations

Several limitations should be considered. First, the search was limited to PubMed and the Cochrane Library, so non-indexed local, regional, or grey literature outputs may have been missed. In particular, the omission of dedicated bibliometric databases such as Scopus and Web of Science, which provide broader coverage of regional journals and citation tracking, is likely to underestimate the total volume of local and non-indexed Saudi otology output and precludes citation-based impact analysis; the reported counts should therefore be interpreted as describing PubMed- and Cochrane-indexed activity rather than the complete national literature. Second, first-author affiliation was used as a pragmatic proxy for institutional and regional research leadership, which may not fully capture shared authorship, multicenter governance, or collaborative contributions. Third, funding and conflict-of-interest data were derived primarily from indexed records and available publication disclosures and may therefore underestimate true financial, institutional, or in-kind support. Fourth, ICD-11 mapping enabled structured topical classification, but some studies addressed more than one condition or could plausibly fit more than one category; therefore, assigning a single primary topic involved reviewer judgment. Fifth, because this was a scoping and bibliometric review, no formal study-level risk-of-bias or quality appraisal was performed. Finally, bibliometric patterns describe publication activity and research visibility but cannot establish study quality, clinical impact, policy uptake, or causal effects of national reforms.

## 5. Conclusions

The PubMed- and Cochrane-indexed Saudi otology literature expanded substantially between 1978 and 2024, with modeled acceleration around the 2017 breakpoint, but remained concentrated by institution, region, topic, and study design. Output was led by a small number of first-author institutions and was geographically concentrated in Riyadh, while hearing impairment, cochlear implantation, and health service-related categories accounted for a large share of the research portfolio. Observational studies predominated, whereas experimental and multicenter studies remained uncommon. Funding visibility was limited overall and increased only modestly as a proportion of total output. These findings provide a baseline for research planning and suggest the need to broaden institutional and regional participation, diversify otology research topics, strengthen multicenter infrastructure, and improve transparent reporting of funding and conflicts of interest.

## Figures and Tables

**Table 1:** Monthly publication counts and seasonality tests: monthly publication counts (Jan–Dec) and expected counts under (i) uniform and (ii) chi2 contributions.

Month	Number of Papers	Expected Counts (Uniform)	Chi2 Contribution (Uniform)
January	39	41.5	0.15060241
February	36	41.5	0.728915663
March	46	41.5	0.487951807
April	37	41.5	0.487951807
May	31	41.5	2.656626506
June	36	41.5	0.728915663
July	39	41.5	0.15060241
August	43	41.5	0.054216867
September	39	41.5	0.15060241
October	58	41.5	6.560240964
November	49	41.5	1.355421687
December	45	41.5	0.295180723

**Table 2:** Top journals publishing otology research from Saudi Arabia: journals with ≥5 publications between 1978 and 2024, ordered by frequency.

Journal	N (%)
*Saudi Med. J.*	44 (8.63)
*Int. J. Pediatr. Otorhinolaryngol.*	36 (7.06)
*Eur. Arch. Otorhinolaryngol.*	24 (4.71)
*Cureus*	22 (4.31)
*Ann. Saudi Med.*	22 (4.31)
*J. Laryngol. Otol.*	19 (3.73)
*Ear Nose Throat J.*	15 (2.94)
*Otol. Neurotol.*	10 (1.96)
*Sci. Rep.*	7 (1.37)
*Neurosciences (Riyadh)*	7 (1.37)
*Laryngoscope*	7 (1.37)
*J. Family Med. Prim. Care*	6 (1.18)
*Am. J. Case Rep.*	5 (0.98)
*World Neurosurg.*	5 (0.98)
*PLoS One*	5 (0.98)
*J. Int. Adv. Otol.*	5 (0.98)
*J. Family Community Med.*	5 (0.98)
*Int. J. Surg. Case Rep.*	5 (0.98)
*Int. J. Audiol.*	5 (0.98)
*Indian J. Otolaryngol. Head Neck Surg.*	5 (0.98)
*Cochlear Implants Int.*	5 (0.98)

Notes: The percentages are relative to all 510 included studies. The remaining journals each contributed < 1% of the output.

## Data Availability

The data presented in this study are included within the article and its Supplementary Materials. The full extracted dataset of included studies is provided as Data File S1, and the SCImago Journal Rank scores and quartile classifications are provided as Data File S2; no additional data were generated.

## Supplementary Materials

The following supporting information can be downloaded at: https://www.mdpi.com/article/10.3390/audiolres16030094/s1, Table S1: Search strategies and PRISMA-S reporting; Methods S1: ICD-11 mapping decision rules; Methods S2: Affiliation cleaning and institutional harmonization rules; Table S2: ICD-11 top-k concentration of focus categories; Figure S1: ICD-11 top-k concentration of focus categories; Table S3: HAC maxlags sensitivity for single-slope NB2 APC; Figure S2: HAC maxlags sensitivity for single-slope NB2 APC; Table S4: Leave-one-year-out APC stability analysis; Figure S3: Leave-one-year-out APC stability analysis; Table S5: Breakpoint AIC scan grid and ΔAIC ≤ 2 confidence set; Figure S4: Breakpoint AIC scan grid and ΔAIC ≤ 2 confidence set; Table S6: Piecewise NB2 model with 2017 hinge breakpoint; Table S7: Residual autocorrelation diagnostics; Table S8: Global counts dataset; Figure S5: Residual autocorrelation diagnostics for the single-slope NB2 model; Figure S6: Residual autocorrelation diagnostics for the piecewise NB2 model; Data File S1: Extracted dataset of included studies; Data File S2: Scimago Journal Rank scores and quartile classifications. Supplementary Checklist S1. PRISMA-ScR Checklist [50].
