# Supplementary material for: Temporal Trends and ICD-11-Mapped Patterns of Otology Research in Saudi Arabia, 1978–2024: A Scoping Review Using Negative Binomial Modelling
Source: Audiol Res. 2026 Jun 22;16(3):94. doi: 10.3390/audiolres16030094 (PMC13295472; doi:10.3390/audiolres16030094)
Supplement: Supplementary file 1 [file audiolres-16-00094-s001.zip › audiolres-4382950-supplementary.pdf]

Supplementary information

Checklist S1.Preferred Reporting Items for Systematic reviews and Meta-Analyses extension for Scoping Reviews (PRISMA-ScR) Checklist [50]

SECTION	ITEM	PRISMA-ScR CHECKLIST ITEM	REPORTED ON PAGE #
**TITLE**			
Title	1	Identify the report as a scoping review.	1
**ABSTRACT**			
Structured summary	2	Provide a structured summary that includes (as applicable): background, objectives, eligibility criteria, sources of evidence, charting methods, results, and conclusions that relate to the review questions and objectives.	1
**INTRODUCTION**			
Rationale	3	Describe the rationale for the review in the context of what is already known. Explain why the review questions/objectives lend themselves to a scoping review approach.	2
Objectives	4	Provide an explicit statement of the questions and objectives being addressed with reference to their key elements (e.g., population or participants, concepts, and context) or other relevant key elements used to conceptualize the review questions and/or objectives.	3
**METHODS**			
Protocol and registration	5	Indicate whether a review protocol exists; state if and where it can be accessed (e.g., a Web address); and if available, provide registration information, including the registration number.	3
Eligibility criteria	6	Specify characteristics of the sources of evidence used as eligibility criteria (e.g., years considered, language, and publication status), and provide a rationale.	3
Information sources*	7	Describe all information sources in the search (e.g., databases with dates of coverage and contact with authors to identify additional sources), as well as the date the most recent search was executed.	3
Search	8	Present the full electronic search strategy for at least 1 database, including any limits used, such that it could be repeated.	3; full strategy in Table S1
Selection of sources of evidence†	9	State the process for selecting sources of evidence (i.e., screening and eligibility) included in the scoping review.	3
Data charting process‡	10	Describe the methods of charting data from the included sources of evidence (e.g., calibrated forms or forms that have been tested by the team before their use, and whether data charting was done independently or in duplicate) and any processes for obtaining and confirming data from investigators.	4
Data items	11	List and define all variables for which data were sought and any assumptions and simplifications made.	4,5,6
Critical appraisal of individual sources of evidence§	12	If done, provide a rationale for conducting a critical appraisal of included sources of evidence; describe the methods used and how this information was used in any data synthesis (if appropriate).	NA
Synthesis of results	13	Describe the methods of handling and summarizing the data that were charted.	4-6
**RESULTS**			
Selection of sources of evidence	14	Give numbers of sources of evidence screened, assessed for eligibility, and included in the review, with reasons for exclusions at each stage, ideally using a flow diagram.	6
Characteristics of sources of evidence	15	For each source of evidence, present characteristics for which data were charted and provide the citations.	6-12
Critical appraisal within sources of evidence	16	If done, present data on critical appraisal of included sources of evidence (see item 12).	NA
Results of individual sources of evidence	17	For each included source of evidence, present the relevant data that were charted that relate to the review questions and objectives.	6-13
Synthesis of results	18	Summarize and/or present the charting results as they relate to the review questions and objectives.	6-13
**DISCUSSION**			
Summary of evidence	19	Summarize the main results (including an overview of concepts, themes, and types of evidence available), link to the review questions and objectives, and consider the relevance to key groups.	13-15
Limitations	20	Discuss the limitations of the scoping review process.	15
Conclusions	21	Provide a general interpretation of the results with respect to the review questions and objectives, as well as potential implications and/or next steps.	15-16
**FUNDING**			
Funding	22	Describe sources of funding for the included sources of evidence, as well as sources of funding for the scoping review. Describe the role of the funders of the scoping review.	16

JBI = Joanna Briggs Institute; PRISMA-ScR = Preferred Reporting Items for Systematic reviews and Meta-Analyses extension for Scoping Reviews.
* Where *sources of evidence* (see second footnote) are compiled from, such as bibliographic databases, social media platforms, and Web sites.
† A more inclusive/heterogeneous term used to account for the different types of evidence or data sources (e.g., quantitative and/or qualitative research, expert opinion, and policy documents) that may be eligible in a scoping review as opposed to only studies. This is not to be confused with *information sources* (see first footnote).
‡ The frameworks by Arksey and O'Malley (6) and Levac and colleagues (7) and the JBI guidance (4, 5) refer to the process of data extraction in a scoping review as data charting.
§ The process of systematically examining research evidence to assess its validity, results, and relevance before using it to inform a decision. This term is used for items 12 and 19 instead of "risk of bias" (which is more applicable to systematic reviews of interventions) to include and acknowledge the various sources of evidence that may be used in a scoping review (e.g., quantitative and/or qualitative research, expert opinion, and policy document).

Table (S1): Search strategies and PRISMA-S reporting

Final update: October 2, 2025, **09:00 (Asia/Riyadh)**

Initial pilot runs: January 2025

ID	Database	Platform	Coverage (pub yrs)	Fields & filters	Last run (date & time, TZ)	Ran by	Export format	Records retrieved
**P1**	PubMed	NIH web	1978–2024	MeSH + all fields; Humans; no language limit; no design filters	2025-Oct-02 09:00 (Asia/Riyadh)	NKK	**.nbib** (All fields)	700
**P2**	PubMed	NIH web	1978–2024	Title/Abstract keywords; MeSH mapping as available; Humans; no language limit	2025-Oct-02 09:00 (Asia/Riyadh)	NKK	**.nbib** (All fields)	631
**P3**	PubMed	NIH web	1978–2024	Title/abstract (tiab); Affiliation (Saudi Arabia); Humans; no language limit	2025-Oct-02 09:00 (Asia/Riyadh)	NKK	**.nbib** (All fields)	891
**C1**	Cochrane Library	Wiley	1978–2024	MeSH + ti,ab,kw; no language limit	2025-Oct-02 09:00 (Asia/Riyadh)	NKK	**.RIS**	5

For keyword-only (tiab) and affiliation queries (P2–P3), year filters began later (1983/1987) to reflect indexing coverage; the MeSH/all-fields query (P1) covered 1978–2024, ensuring that overall coverage matched the review window.

P1 — PubMed (MeSH/all fields, Saudi Arabia)

(((("Ear"[Mesh]) OR "Ear Diseases"[Mesh]) OR "Labyrinth Diseases"[Mesh]) OR ("Ear Auricle"[Mesh] OR "Acupuncture, Ear"[Mesh] OR "Cholesteatoma, Middle Ear"[Mesh] OR "Round Window, Ear"[Mesh] OR "Oval Window, Ear"[Mesh] OR "Middle Ear Ventilation"[Mesh] OR "Ear, Inner"[Mesh] OR "Ear, Middle"[Mesh] OR

"Ear, External"[Mesh] OR "Ear Protective Devices"[Mesh] OR "Ear Ossicles"[Mesh] OR "Ear Neoplasms"[Mesh] OR "Ear Deformities, Acquired"[Mesh] OR "Ear Cartilage"[Mesh] OR "Ear Canal"[Mesh] OR "Otitis"[Mesh] OR "Otitis Media with Effusion"[Mesh] OR "Ossicular Prosthesis"[Mesh])) OR ("Otolaryngology"[Mesh] OR "Neurotology"[Mesh])) OR "Skull Base"[Mesh]) AND ("Saudi Arabia"[Mesh] OR "Saudi Arabia"[All Fields])

Filters: 1978–2024

P2 — PubMed (procedures/conditions keywords + Saudi)

("Facial nerve decompression" OR "Otoplasty" OR "Tympanoplasty" OR "Ossiculoplasty" OR "Mastoidectomy" OR "Stapedectomy" OR "Stapedotomy" OR "Cholesteatoma Surgery" OR "Cochlear Implant" OR "Labyrinthectomy" OR "Endolymphatic Sac Surgery" OR "Translabyrinthine Approach" OR "Middle Fossa Approach" OR "Retrosigmoid/Suboccipital Approach" OR "Glomus Tumor Surgery" OR "Eustachian Tube Balloon Dilation" OR "Particle Repositioning Maneuver" OR "Intratympanic Injections" OR "Tympanomastoidectomy" OR "Stapedectomy" OR "Labyrinthectomy" OR "SNHL" OR "Hearing loss" OR "Hearing aid" OR "Bone conduction") AND ("Saudi Arabia"[Mesh] OR "Saudi Arabia"[All Fields])

Filters: 1983–2024

P3 — PubMed (tiab ear terms + Saudi Affiliation)

("ear"[tiab] OR "Malignant otitis externa"[tiab] OR "Otomycosis"[tiab] OR "Cholesteatoma"[tiab] OR "myringitis"[tiab] OR "Tympanosclerosis"[tiab] OR "Adhesive middle ear disease"[tiab] OR "dislocation of ear ossicles"[tiab] OR "Discontinuity of ear ossicles"[tiab] OR "Polyp of middle ear"[tiab] OR "Middle ear cicatrix"[tiab] OR "hearing loss"[tiab] OR "Otosclerosis"[tiab] OR "vestibular syndrome"[tiab] OR "Disorders of vestibular function"[tiab] OR "Labyrinthine fistula"[tiab] OR "Labyrinthine dysfunction"[tiab] OR "Noise effects on inner ear"[tiab] OR "Congenital hearing impairment"[tiab] OR "Acquired hearing impairment"[tiab] OR "Deafness"[tiab] OR "Ototoxic"[tiab] OR "Presbycusis"[tiab] OR "Sudden idiopathic hearing loss"[tiab] OR "Hereditary hearing loss"[tiab] OR "Auditory synaptopathy"[tiab] OR "Auditory neuropathy"[tiab] OR "Otalgia"[tiab] OR "effusion of ear"[tiab] OR "Disorders of acoustic nerve"[tiab] OR "Atrophy ear"[tiab] OR "postmastoidectomy cavity"[tiab] OR "eustachian apparatus"[tiab] OR "pinnae"[tiab] OR "Otocephaly"[tiab] OR "Accessory auricle"[tiab] OR "Foreign body in ear"[tiab])

AND Saudi Arabia[Affiliation]

Filters: 1987–2024

C1 — Cochrane library (MeSH + ti,ab,kw)

(#1 OR #2 OR #3) AND #4

#1 [mh "Ear"] OR [mh "Ear Diseases"] OR [mh "Labyrinth Diseases"] OR [mh "Ear Auricle"] OR [mh "Acupuncture, Ear"] OR [mh "Cholesteatoma, Middle Ear"] OR [mh "Round Window, Ear"] OR [mh "Oval Window, Ear"] OR [mh "Middle Ear Ventilation"] OR [mh "Ear, Inner"] OR [mh "Ear, Middle"] OR [mh "Ear, External"] OR [mh "Ear Protective Devices"] OR [mh "Ear Ossicles"] OR [mh "Ear Neoplasms"] OR [mh "Ear Deformities, Acquired"] OR [mh "Ear Cartilage"] OR [mh "Ear Canal"] OR [mh "Otitis"] OR [mh "Otitis Media with Effusion"] OR [mh "Ossicular Prosthesis"] OR [mh "Otolaryngology"] OR [mh "Neurotology"] OR [mh "Skull Base"]

#2 "Facial nerve decompression":ti,ab,kw OR "Otoplasty":ti,ab,kw OR "Tympanoplasty":ti,ab,kw OR "Ossiculoplasty":ti,ab,kw OR "Mastoidectomy":ti,ab,kw OR "Stapedectomy":ti,ab,kw OR "Stapedotomy":ti,ab,kw OR "Cholesteatoma Surgery":ti,ab,kw OR "Cochlear Implant":ti,ab,kw OR "Labyrinthectomy":ti,ab,kw OR "Endolymphatic Sac Surgery":ti,ab,kw OR "Translabyrinthine Approach":ti,ab,kw OR "Middle Fossa Approach":ti,ab,kw OR "Retrosigmoid/Suboccipital Approach":ti,ab,kw OR "Glomus Tumor Surgery":ti,ab,kw OR "Eustachian Tube Balloon Dilation":ti,ab,kw OR "Particle Repositioning Maneuver":ti,ab,kw OR "Intratympanic Injections":ti,ab,kw OR "Tympanomastoidectomy":ti,ab,kw OR "SNHL":ti,ab,kw OR "Hearing loss":ti,ab,kw OR "Hearing aid":ti,ab,kw OR "Bone conduction":ti,ab,kw

#3 "ear":ti,ab,kw OR "Malignant otitis externa":ti,ab,kw OR "Otomycosis":ti,ab,kw OR "Cholesteatoma":ti,ab,kw OR "myringitis":ti,ab,kw OR "Tympanosclerosis":ti,ab,kw OR "Adhesive middle ear disease":ti,ab,kw OR "dislocation of ear ossicles":ti,ab,kw OR "Discontinuity of ear ossicles":ti,ab,kw OR "Polyp of middle ear":ti,ab,kw OR "Middle ear cicatrix":ti,ab,kw OR "Otosclerosis":ti,ab,kw OR "vestibular syndrome":ti,ab,kw OR "Disorders of vestibular function":ti,ab,kw OR "Labyrinthine fistula":ti,ab,kw OR "Labyrinthine dysfunction":ti,ab,kw OR "Noise effects on inner ear":ti,ab,kw OR "Congenital hearing impairment":ti,ab,kw OR "Acquired hearing impairment":ti,ab,kw OR "Deafness":ti,ab,kw OR "Ototoxic":ti,ab,kw OR "Presbycusis":ti,ab,kw OR "Sudden idiopathic hearing loss":ti,ab,kw OR "Hereditary hearing loss":ti,ab,kw OR

"Auditory synaptopathy":ti,ab,kw OR "Auditory neuropathy":ti,ab,kw OR "Otalgia":ti,ab,kw OR "effusion of ear":ti,ab,kw OR "Disorders of acoustic nerve":ti,ab,kw OR "Atrophy ear":ti,ab,kw OR "postmastoidectomy cavity":ti,ab,kw OR "eustachian apparatus":ti,ab,kw OR "pinnae":ti,ab,kw OR "Otocephaly":ti,ab,kw OR "Accessory auricle":ti,ab,kw OR "Foreign body in ear":ti,ab,kw

#4 [mh "Saudi Arabia"]

Notes: Coverage refers to the **publication years targeted** (1978–2024). The databases were searched from **inception** to the last run date. Exports were captured on the same day as the final run. Duplicates were removed using the native duplicate-detection workflow (Title/DOI/PMID) in **Zotero v6** with manual verification.

Methods (S1): ICD-11 mapping decision rules

- Primary assignment: Each record is mapped to one ICD-11 focus chapter that best reflects the stated primary aim.
- Ambiguity rule: If multiple chapters are targeted, the first author-provided keyword is used alongside the study’s focus, as reported in the Methods section (population/intervention/outcome), to determine the dominant chapter.
- Edge cases: If no keywords are provided, this review relies on the title + abstract focus; if the situation is still ambiguous, the more specific chapter is used for classification to avoid excessively broad categories.
- Verification: The verifier confirms the chapter → block → category path in the ICD browser/coding tool; disagreements are resolved through consensus.

Methods (S2): Affiliation cleaning and institutional harmonisation rules

All publications originally listed as “King Faisal University (KFU)” in Dammam prior to 2001 were reassigned to “Imam Abdulrahman Bin Faisal University” to reflect the institutional split. In addition, publications listed as “Dammam University” prior to 2016 were standardized to “Imam Abdulrahman Bin Faisal University” to account for the subsequent renaming.

The following universities are associated with their respective teaching hospital

- Tabuk University—Tabuk University Hospital

- King Saud bin Abdulaziz University for Health Sciences—King Abdulaziz Medical City
- King Saud University—King AbdulAziz University Hospital (KAUH) (Riyadh) and King Khalid University Hospital (KKUH)
- Princess Nourah Bint Abdulrahman University—King Abdullah bin Abdulaziz University Hospital (KAUH)
- Najran University—Najran University Hospital (NUH)
- King Abdulaziz University—King Abdulaziz University Hospital (KAUH) (Jeddah)
- Jazan University—Jazan University Hospital
- Imam Abdulrahman Bin Faisal University—King Fahd Hospital of the University (KFHU)
- Qassim University—Qassim University Medical City
- Prince Sattam bin Abdulaziz University—Prince Sattam University Hospital
- King Faisal University—King Fahd Hospital of the University (KFHU)

Table S2. ICD-11 top-k concentration of focus categories (k=2,3,5)

K	Top-k categories	Top-k share (%)
**2**	Presence of device, implants or grafts, Disorders with hearing impairment	44.7
**3**	Presence of device, implants or grafts, Disorders with hearing impairment, Diseases of middle ear or mastoid	54.7
**5**	Presence of device, implants or grafts, Disorders with hearing impairment, Diseases of middle ear or mastoid, Contact with health services for specific surgical interventions, Developmental anomalies	70.2

Top-k concentration of ICD-11 categories (k = 2, 3, 5) with shares (%).

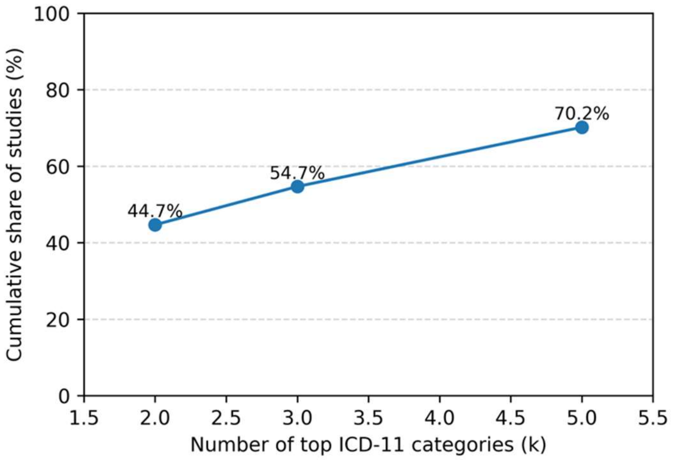

Figure S1. ICD-11 top-k concentration of focus categories (k=2,3,5)

Table S3. HAC maxlags sensitivity for single-slope NB2 APC (MLE vs HAC(1–3))

Method	Beta	SE	APC	CI_lower	CI_upper	CI_width	SE_inflation_%
MLE	0.088753426201840 98	0.007135227151629355	9.281116450338223	7.763479825845643	10.820126001240782	3.056646175395139	0.0
HAC(1)	0.088753426201840 98	0.008684299390305763	9.281116450338223	7.43679252756313	11.157101135242353	3.7203086076792236	21.710202152746767
HAC(2)	0.088753426201840 98	0.009717569470157823	9.281116450338223	7.21943469602373	11.38244149943526	4.1630068034115295	36.19145212411045
HAC(3)	0.088753426201840 98	0.010503727434064572	9.281116450338223	7.054353777781985	11.554196454464005	4.49984267668202	47.20943301245858

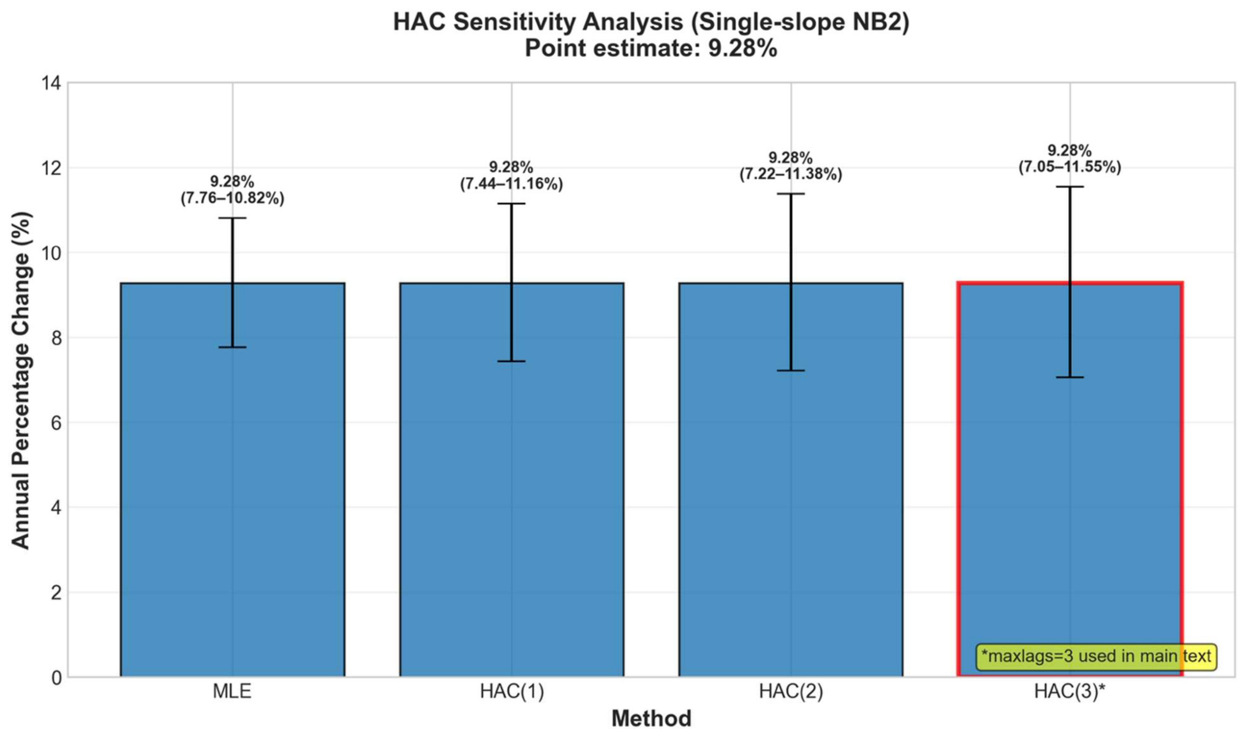

Figure S2. HAC maxlags sensitivity for single-slope NB2 APC (MLE vs HAC(1–3))

Table S4. Leave-one-year-out (LOYO) APC stability summary and per-iteration values

left_out_year	APC_pct
1978	9.32400700657443
1979	9.167270344065393
1980	9.162391352978805
1981	9.15759525795725
1982	9.152902896336368
1983	9.266168990567735
1984	9.14415193560778
1985	9.24446654023592
1986	9.234233145686922
1987	9.318769710155284
1988	9.303036594410564
1989	9.128420497003953
1990	9.126870301994373
1991	9.402281466871054
1992	9.378058524310573

1993	9.29679020425378
1994	9.499523107882691
1995	9.316104851240015
1996	9.794700115413658
1997	9.479918080746286
1998	9.272933728779664
1999	9.29090574044289
2000	9.376804900288892
2001	9.250671815796952
2002	9.364218338488417
2003	9.285869120535416
2004	9.288364804704518
2005	9.246736970411895
2006	9.255992169761829
2007	9.277183747939732
2008	9.281631617548825
2009	9.294007415824979
2010	9.301796453065503
2011	9.267478333737444
2012	9.2932810248616
2013	9.350759891272409
2014	9.295637540253754
2015	9.33107988413109
2016	9.382072759544325
2017	9.376227690522532
2018	9.403185709512552
2019	9.24100743076497
2020	9.258402804389142
2021	9.147622726138849
2022	9.135123178623772
2023	9.176083142846858
2024	8.912787032789593

LOYO APC% mean=9.28, min=8.91, max=9.79; problematic fits=0

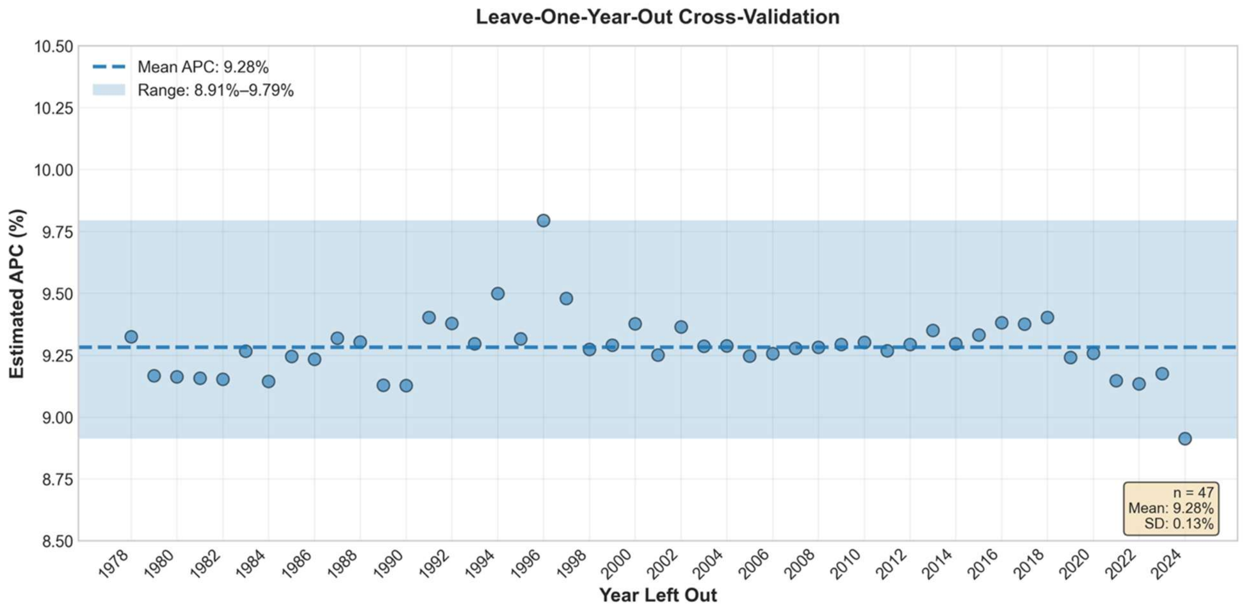

Figure S3. Leave-one-year-out (LOYO) APC stability summary and per-iteration values

Table S5. Breakpoint AIC scan grid and ΔAIC≤2 confidence set

bp	AIC	converged
1985	249.70400592302425	True
1986	249.0553975052055	True
1987	248.49095021980207	True
1988	248.24594274414252	True
1989	248.1724196480199	True
1990	247.74128627403772	True
1991	246.99453450437304	True
1992	246.68130939200742	True
1993	246.66558364639104	True
1994	246.68911260528512	True
1995	247.17308100761846	True
1996	247.6306666335631	True
1997	248.83314139952753	True
1998	250.05418647904247	True
1999	250.9406255289252	True
2000	251.59915966476748	True
2001	252.15244392240967	True
2002	252.47134656293983	True

2003	252.64335560226672	True
2004	252.57551951299732	True
2005	252.23103773039404	True
2006	251.69766801158738	True
2007	251.08962521196003	True
2008	250.3488573302782	True
2009	249.67422074228523	True
2010	249.14447507184028	True
2011	248.75844938825182	True
2012	247.99080006949526	True
2013	246.9620269528774	True
2014	246.26694473046618	True
2015	245.21324975180673	True
2016	244.0454015851836	True
2017	243.62528944279734	True

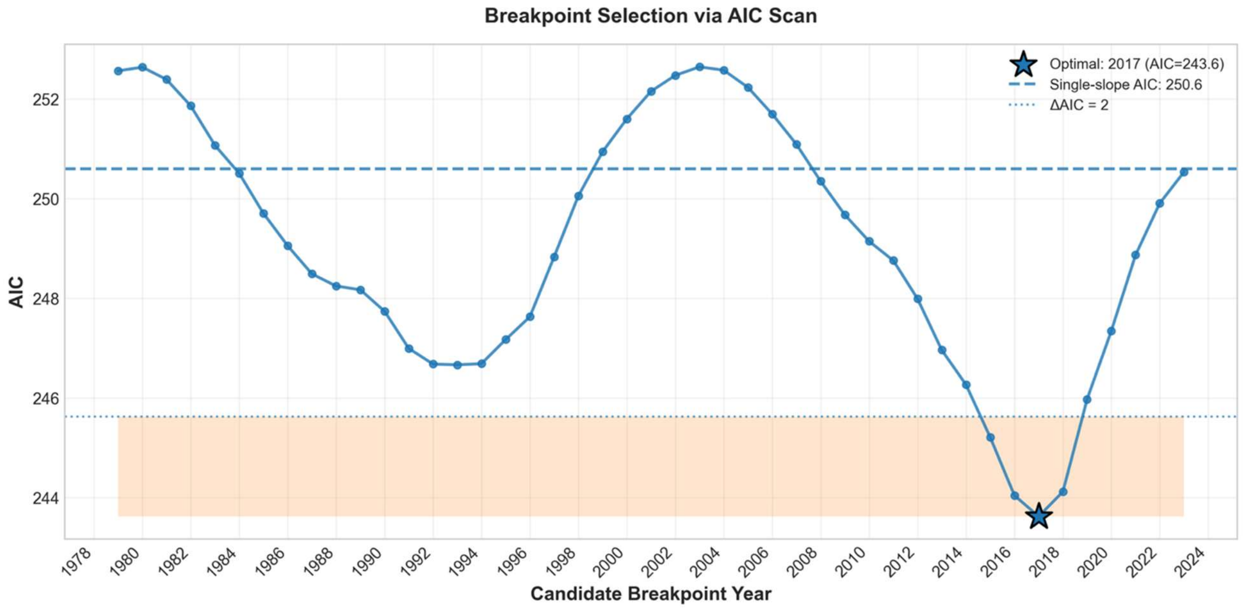

Figure S4. Breakpoint AIC scan grid and ΔAIC≤2 confidence set

Table S6A. Piecewise NB2 (2017 hinge) coefficients and HAC(1) inference

Best breakpoint year (hinge)	2017
AIC (piecewise NB2)	243.6
AIC (single-slope)	250.6

ΔAIC	-7.0
alpha	0.046402

Table S6B. Piecewise NB2 (2017 hinge) coefficients and HAC(1) inference

	Beta	SE HAC (1)	APC (%)	95% CI	*p*-value
Pre-2017	0.069369	0.009004	7.2	5.3–9.1	$p < 0.001$
Hinge	0.145104	0.027695	-	-	$p < 0.001$
Post-2017	0.214473	0.022251	23.9	18.6–29.4	$p < 0.001$

Table S7. Residual autocorrelation diagnostics

Model	Lag 1	Lag 2	Lag 3	Significance threshold (±2/√n)
Single-slope NB2	0.415	0.352	0.284	0.292
Piecewise NB2	0.324	0.261	0.218	0.292

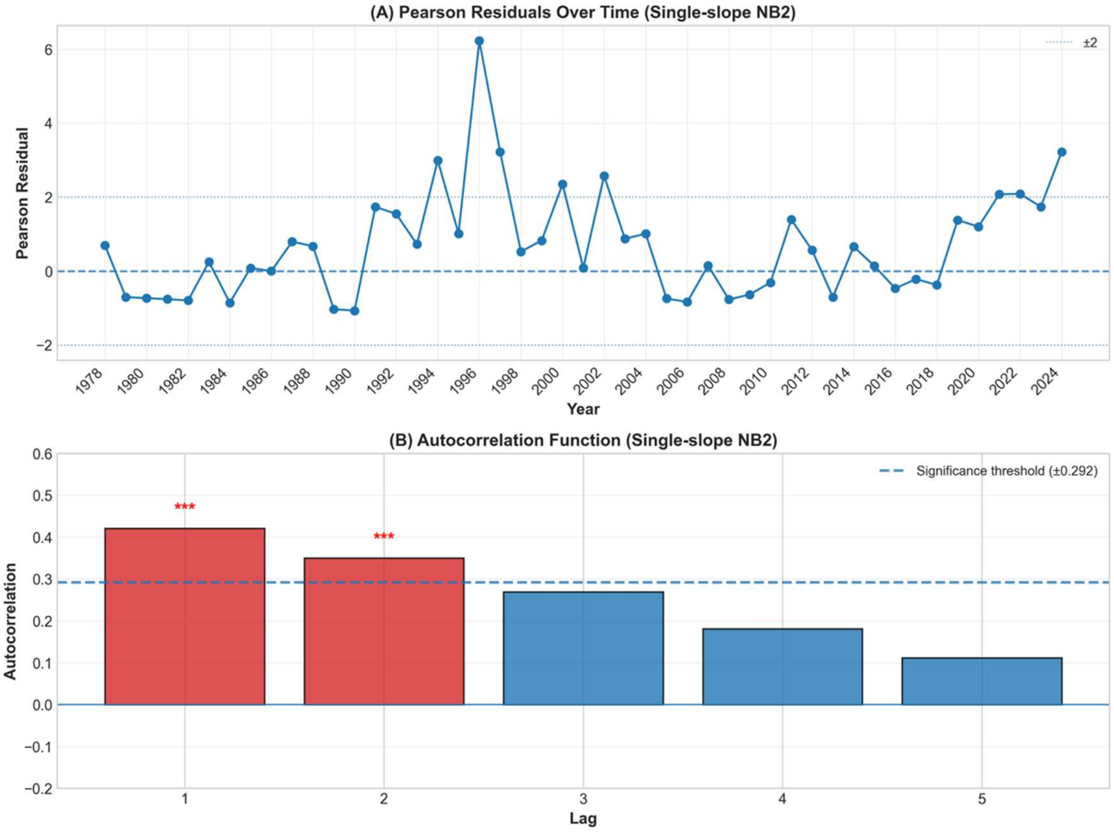

Figure S5. Residual autocorrelation diagnostics (Single-slope NB2)

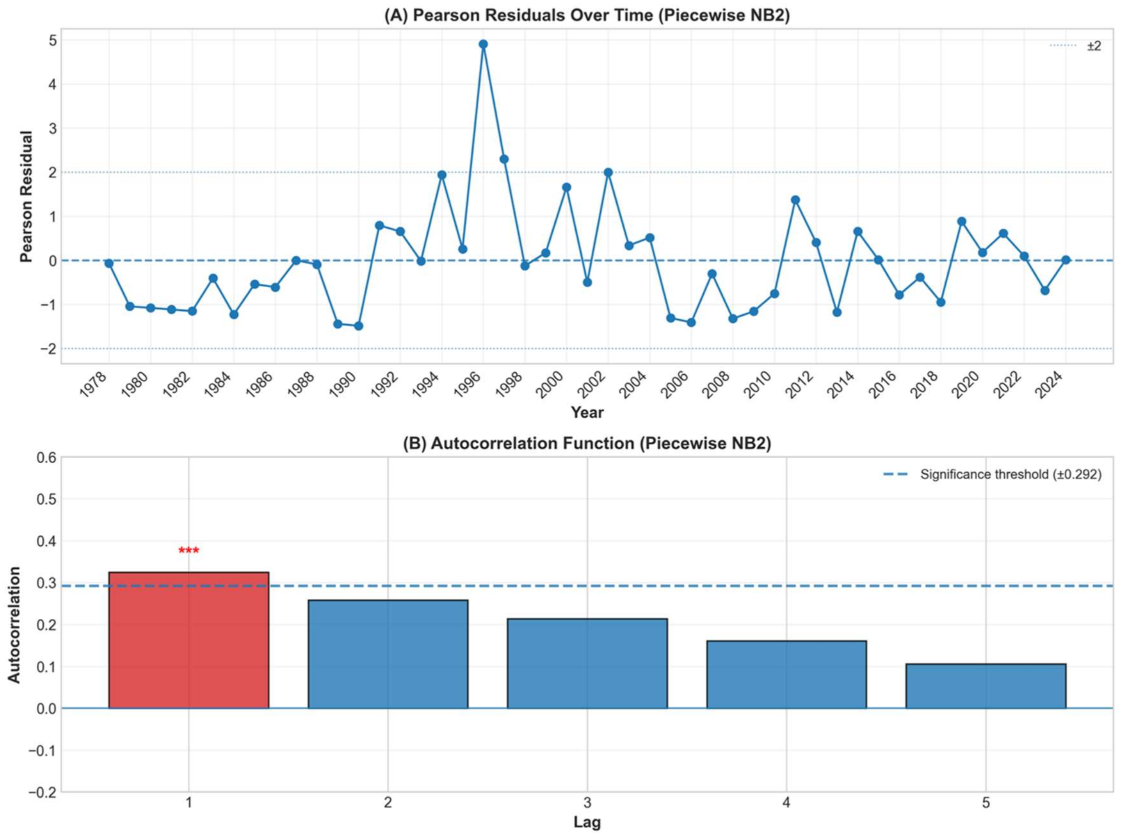

Figure S6. Residual autocorrelation diagnostics (Piecewise NB2)

Table S8: Global counts dataset

Comparator query (PubMed; publication date; 1978–2024; Saudi-affiliated records excluded): ("ear"[tiab] OR "Malignant otitis externa"[tiab] OR "Otomycosis"[tiab] OR "Cholesteatoma"[tiab] OR "myringitis"[tiab] OR "Tympanosclerosis"[tiab] OR "Adhesive middle ear disease"[tiab] OR "dislocation of ear ossicles"[tiab] OR "Discontinuity of ear ossicles"[tiab] OR "Polyp of middle ear"[tiab] OR "Middle ear cicatrix"[tiab] OR "hearing loss"[tiab] OR "Otosclerosis"[tiab] OR "vestibular syndrome"[tiab] OR "Disorders of vestibular function"[tiab] OR "Labyrinthine fistula"[tiab] OR "Labyrinthine dysfunction"[tiab] OR "Noise effects on inner ear"[tiab] OR "Congenital hearing impairment"[tiab] OR "Acquired hearing impairment"[tiab] OR "Deafness"[tiab] OR "Ototoxic"[tiab] OR "Presbycusis"[tiab] OR "Sudden idiopathic hearing loss"[tiab] OR "Hereditary hearing loss"[tiab] OR "Auditory synaptopathy"[tiab] OR "Auditory neuropathy"[tiab] OR "Otalgia"[tiab] OR "effusion of ear"[tiab] OR "Disorders of acoustic nerve"[tiab] OR "Atrophy ear"[tiab] OR "postmastoidectomy cavity"[tiab] OR "eustachian apparatus"[tiab] OR "pinnae"[tiab] OR "Otocephaly"[tiab] OR "Accessory auricle"[tiab] OR "Foreign body in ear"[tiab] OR "Facial nerve decompression" OR "Otoplasty" OR "Tympanoplasty" OR "Ossiculoplasty" OR "Mastoidectomy" OR "Stapedectomy" OR "Stapedotomy" OR "Cholesteatoma Surgery" OR "Cochlear Implant" OR "Labyrinthectomy" OR

"Endolymphatic Sac Surgery" OR "Translabyrinthine Approach" OR "Middle Fossa Approach" OR "Retrosigmoid/Suboccipital Approach" OR "Glomus Tumor Surgery" OR "Eustachian Tube Balloon Dilation" OR "Particle Repositioning Maneuver" OR "Intratympanic Injections" OR "Tympanomastoidectomy" OR "Hearing loss" OR "Hearing aid" OR "Bone conduction" OR "Ear"[Mesh] OR "Ear Diseases"[Mesh] OR "Labyrinth Diseases"[Mesh] OR "Ear Auricle"[Mesh] OR "Acupuncture, Ear"[Mesh] OR "Cholesteatoma, Middle Ear"[Mesh] OR "Round Window, Ear"[Mesh] OR "Oval Window, Ear"[Mesh] OR "Middle Ear Ventilation"[Mesh] OR "Ear, Inner"[Mesh] OR "Ear, Middle"[Mesh] OR "Ear, External"[Mesh] OR "Ear Protective Devices"[Mesh] OR "Ear Ossicles"[Mesh] OR "Ear Neoplasms"[Mesh] OR "Ear Deformities, Acquired"[Mesh] OR "Ear Cartilage"[Mesh] OR "Ear Canal"[Mesh] OR "Otitis"[Mesh] OR "Otitis Media with Effusion"[Mesh] OR "Ossicular Prosthesis"[Mesh] OR "Neurotology"[Mesh] OR "Skull Base"[Mesh]) NOT ("Saudi Arabia"[Affiliation])

Note: the comparator uses the ear- and otology-specific terms of the primary search. The whole-specialty heading "Otolaryngology"[Mesh] and the abbreviation "SNHL" (present in the primary search) were not included, because—searched globally and on unscreened counts—"Otolaryngology"[Mesh] would broaden the corpus well beyond otology.

Overdispersion φ=32.16. Negative binomial estimated RR/year=1.035 (95% CI 1.034–1.037; p=0.00e+00); annual increase ~3.5%.

NB2 alpha (dispersion)=0.005.

AIC=705.3

Breakpoint search (same hinge procedure as the primary analysis): the best-supported change in slope was at 1996 (piecewise NB2 AIC = 675.9 vs single-slope 705.3; ΔAIC = −29.4), with pre-1996 ≈ 2.5%/year and post-1996 ≈ 4.1%/year. A hinge at 2017 did not improve fit (AIC above the single-slope model). The overall single-slope rate above is reported as the comparator summary.

year	n
1978	2956
1979	3094
1980	3101
1981	3081
1982	3031
1983	3405
1984	3353
1985	3480
1986	3387
1987	3395
1988	3762

1989	3751
1990	4009
1991	4331
1992	3924
1993	4313
1994	4161
1995	4605
1996	4267
1997	4907
1998	4888
1999	5074
2000	5407
2001	5237
2002	5366
2003	5523
2004	5790
2005	6436
2006	6584
2007	7059
2008	7262
2009	7572
2010	8145
2011	8794
2012	9144
2013	9689
2014	9937
2015	10168
2016	10313
2017	10150
2018	10500
2019	11037
2020	12274
2021	13307
2022	12648
2023	11810
2024	12043

Data File S1

PMID	Title	Authors	Journal/Book	Month	Year	First author institution	Type of Publication	Region	Funding	COI	ICD11 Chapter	ICD11 Block	ICD11 Category
712219	Otomycosis: a survey in the eastern province of Saudi Arabia	Yassin A, Maher A, Moawad MK,	J Laryngol Otol	10	1978	Dr. Fakhry Hospital	Observational Studies	Eastern Province	NA	NA	10 Ear & mastoid	Diseases of External Ear	Infectious diseases of external ear
1304673	Description and evaluation of a simple method for microbiological studies on otitis externa	Dibb WL,	Undersea Biomed Res	11	1992	King Abdulaziz Medical City	Observational Studies	Makkah	None	None	10 Ear & mastoid	Diseases of External Ear	Infectious diseases of external ear
1541892	Malignant external otitis: management policy	el-Silimy O, Sharnuby M,	J Laryngol Otol	1	1992	Riyadh National Hospital	Observational Studies	Riyadh	None	None	10 Ear & mastoid	Diseases of External Ear	Infectious diseases of external ear
1613342	Otological manifestations of thalassaemia intermedia: evidence of temporal bone involvement and report of a unique cholesteatoma-like lesion	Sheikha A, Kameswaran M, Okafor BC, al-Saigh AA,	J Laryngol Otol	4	1992	King Khalid University	Observational Studies	Asir	None	None	10 Ear & mastoid	Disorders with hearing impairment	Hearing impairment (unspecified)
1892575	Cerebello-pontine angle paraganglioma simulating an acoustic neurinoma	Jamjoom ZA, Sadiq S, Naim-Ur-Rahman, Malabary T,	Br J Neurosurg	NA	1991	King Saud University	Observational Studies	Riyadh	None	None	02 Neoplasms	Malignant neoplasms, except primary neoplasms of lymphoid, haematopoietic, central nervous system or related tissues	Malignant neoplasms of endocrine glands
3352436	Sensorineural hearing loss in sickle cell crisis	Elwany S, Kamel T,	Laryngoscope	4	1988	Dr. Fakhry Hospital	Observational Studies	Eastern Province	None	None	10 Ear & mastoid	Disorders with hearing impairment	Acquired hearing impairment

PMID	Title	Authors	Journal/Book	Month	Year	First author institution	Type of Publication	Region	Funding	COI	ICD11 Chapter	ICD11 Block	ICD11 Category
3502086	Acoustic neuroma presenting as a cyst with fluid level	Patel PJ, Kolawole TM,	J Comput Tomogr	10	1987	King Saud University	Observational Studies	Riyadh	None	None	02 Neoplasms	Neoplasms of central nervous system or related structures	Primary neoplasm of spinal cord, cranial nerves, paraspinal nerves or remaining parts of central nervous system
3694029	The ageing ear. A clinico-pathological classification	Belal A Jr, Glorig A,	J Laryngol Otol	11	1987	El-Maghraby Eye and Ear Hospital	Observational Studies	Makkah	None	None	10 Ear & mastoid	Disorders with hearing impairment	Presbycusis
3755571	Cochlear implantation in developing countries	Belal A,	Am J Otol	7	1986	El-Maghraby Eye and Ear Hospital	Observational Studies	Makkah	None	None	24 Factors influencing health status or contact with health services	Reasons for contact with the health services	Presence of device, implants or grafts
4095769	Sensorineural hearing loss in sickle cell disease patients in Saudi Arabia	Ashoor A, Al-Awamy B,	Trop Geogr Med	12	1985	Imam Abdulrahman Bin Faisal University	Observational Studies	Eastern Province	NA	NA	10 Ear & mastoid	Disorders with hearing impairment	Acquired hearing impairment
7499943	Hearing impairment in association with distal renal tubular acidosis among Saudi children	Zakzouk SM, Sobki SH, Mansour F, al Anazy FH,	J Laryngol Otol	10	1995	King Saud University	Observational Studies	Riyadh	None	None	10 Ear & mastoid	Disorders with hearing impairment	Acquired hearing impairment
7665272	Familial hereditary progressive sensorineural hearing loss among Saudi population	Zakzouk SM, Fadle KA, al Anazy FH,	Int J Pediatr Otorhinolaryngol	7	1995	King Saud University	Observational Studies	Riyadh	None	None	10 Ear & mastoid	Disorders with hearing impairment	Hereditary hearing loss

PMID	Title	Authors	Journal/Book	Month	Year	First author institution	Type of Publication	Region	Funding	COI	ICD11 Chapter	ICD11 Block	ICD11 Category
7808750	Tympanoplasty in children--our experience in Riyadh, Saudi Arabia	Attallah MS,	Otolaryngol Pol	NA	1994	King Saud University	Observational Studies	Riyadh	None	None	24 Factors influencing health status or contact with health services	Reasons for contact with the health services	Contact with health services for specific surgical interventions
8056493	Hearing impairment among children in Saudi Arabia: familial incidence and potential risk factors	Zakzouk SM, Hossain A,	Int J Pediatr Otorhinolaryngol	4	1994	King Saud University	Observational Studies	Riyadh	None	None	10 Ear & mastoid	Disorders with hearing impairment	Congenital hearing impairment
8108156	Tympanic membrane perforation in survivors of a SCUD missile explosion	Patow CA, Bartels J, Dodd KT,	Otolaryngol Head Neck Surg	2	1994	85th Evacuation Hospital	Observational Studies	Eastern Province	None	None	10 Ear & mastoid	Diseases of middle ear or mastoid	Perforation of tympanic membrane
8182312	Relevant demographic factors and hearing impairment in Saudi children: epidemiological study	Bafaqeeh SA, Zakzouk SM, al Muhaimeid H, Essa A,	J Laryngol Otol	4	1994	King Saud University	Observational Studies	Riyadh	None	None	10 Ear & mastoid	Disorders with hearing impairment	Acquired hearing impairment
8288975	Sensorineural hearing loss in neurobrucellosis	Thomas R, Kameswaran M, Murugan V, Okafor BC,	J Laryngol Otol	11	1993	Aseer Central Hospital	Observational Studies	Asir	None	None	10 Ear & mastoid	Disorders with hearing impairment	Acquired hearing impairment
8444552	Epidemiology of chronic suppurative otitis media in Saudi children	Muhaimeid H, Zakzouk S, Bafaqeeh S,	Int J Pediatr Otorhinolaryngol	3	1993	King Saud University	Observational Studies	Riyadh	None	None	10 Ear & mastoid	Diseases of middle ear or mastoid	Suppurative otitis media

PMID	Title	Authors	Journal/Book	Month	Year	First author institution	Type of Publication	Region	Funding	COI	ICD11 Chapter	ICD11 Block	ICD11 Category
8718536	Revision tympanoplasty: surgical findings and results in Riyadh	Attallah MS,	ORL J Otorhinolaryngol Relat Spec	1	1996	King Saud University	Observational Studies	Riyadh	None	None	24 Factors influencing health status or contact with health services	Reasons for contact with the health services	Contact with health services for specific surgical interventions
8723953	Vein graft in stapes surgery	Kamal SA,	Am J Otol	3	1996	King Abdulaziz Medical City	Observational Studies	Riyadh	None	None	24 Factors influencing health status or contact with health services	Reasons for contact with the health services	Contact with health services for specific surgical interventions
8736050	Prevalence of sensorineural hearing loss due to rubella in Saudi children	Zakzouk SM, al-Muhaimeed H,	ORL J Otorhinolaryngol Relat Spec	4	1996	King Saud University	Observational Studies	Riyadh	King Abdulaziz City for science and Technology	None	10 Ear & mastoid	Disorders with hearing impairment	Acquired hearing impairment
8770668	Prevalence of sensorineural hearing loss due to toxoplasmosis in Saudi children: a hospital based study	al Muhaimeed H,	Int J Pediatr Otorhinolaryngol	1	1996	King Saud University	Observational Studies	Riyadh	None	None	10 Ear & mastoid	Disorders with hearing impairment	Acquired hearing impairment
8770675	Hearing impairment among "at risk' children	Zakzouk SM, al-Muhaimeed HS,	Int J Pediatr Otorhinolaryngol	1	1996	King Saud University	Observational Studies	Riyadh	None	None	10 Ear & mastoid	Disorders with hearing impairment	Acquired hearing impairment
8874912	A comparison of ondansetron and prochlorperazine for the prevention of nausea and vomiting after	van den Berg AA,	Can J Anaesth	9	1996	Prince Sultan Military Medical City	Experimental studies	Riyadh	None	None	24 Factors influencing health status or contact with health services	Reasons for contact with the health services	Contact with health services for specific surgical interventions

PMID	Title	Authors	Journal/Book	Month	Year	First author institution	Type of Publication	Region	Funding	COI	ICD11 Chapter	ICD11 Block	ICD11 Category
	tympanoplasty												
8892562	Mastoid abscess: underlying disease and management	al-Serhani AM,	Am J Otol	9	1996	King Saud University	Observational Studies	Riyadh	None	None	10 Ear & mastoid	Diseases of middle ear or mastoid	Mastoiditis or related conditions
8916864	Prevalence of severe to profound sensorineural hearing loss in children having family members with hearing impairment	Zakzouk SM, Bafaqeeh SA,	Ann Otol Rhinol Laryngol	11	1996	King Saud University	Observational Studies	Riyadh	None	None	10 Ear & mastoid	Disorders with hearing impairment	hearing impairment (unspecified)
8972435	Merkel cell carcinoma of the auricle	al-Dousary S, Maqbool S, Zaid MA, al Rikabi A,	J Otolaryngol	12	1996	King Saud University	Observational Studies	Riyadh	None	None	02 Neoplasms	Malignant neoplasms, stated or presumed to be primary, of specified sites, except of lymphoid, haematopoietic, central nervous system or related tissues	Malignant neoplasms of skin
9009673	Hearing results in tympanoplasty in Riyadh	Attallah MS, al-Essa A,	Otolaryngol Pol	1	1996	King Saud University	Systematic review and Meta-Analysis	Riyadh	None	None	24 Factors influencing health status or contact with health services	Reasons for contact with the health services	Contact with health services for specific surgical interventions

PMID	Title	Authors	Journal/Book	Month	Year	First author institution	Type of Publication	Region	Funding	COI	ICD11 Chapter	ICD11 Block	ICD11 Category
9042514	Cochlear implants in Saudi Arabia: experience in King Fahd Hospital	al-Shaikh AH, Metwalli AA, al-Torary N,	Adv Otorhinolaryngol	NA	1997	King Fahad Hospital	Observational Studies	Makkah	None	None	24 Factors influencing health status or contact with health services	Reasons for contact with the health services	Presence of device, implants or grafts
9078824	Hearing loss and herpes simplex	al Muhaimeed H, Zakzouk SM,	J Trop Pediatr	2	1997	King Saud University	Observational Studies	Riyadh	None	None	10 Ear & mastoid	Disorders with hearing impairment	Acquired hearing impairment
9222635	Otological manifestations of primary ciliary dyskinesia	el-Sayed Y, al-Sarhani A, al-Essa AR,	Clin Otolaryngol Allied Sci	6	1997	King Saud University	Observational Studies	Riyadh	None	None	10 Ear & mastoid	Diseases of middle ear or mastoid	Otitis media
9270427	Anaerobes and fungi in chronic suppurative otitis media	Ibekwe AO, al Shareef Z, Benayam A,	Ann Otol Rhinol Laryngol	8	1997	North West Armed Forces Hospital	Observational Studies	Tabuk	None	None	10 Ear & mastoid	Diseases of middle ear or mastoid	Suppurative otitis media
9343776	Epidemiology and etiology of hearing impairment among infants and children in a developing country. Part I	Zakzouk SM,	J Otolaryngol	10	1997	King Saud University	Observational Studies	Riyadh	None	None	10 Ear & mastoid	Disorders with hearing impairment	Acquired hearing impairment
9425477	Surgery of tympanosclerosis	Kamal SA,	J Laryngol Otol	10	1997	King Abdulaziz Medical City	Observational Studies	Riyadh	None	None	24 Factors influencing health status or contact with health services	Reasons for contact with the health services	Contact with health services for specific surgical interventions
9438943	Epidemiology and etiology of hearing impairment among infants and children in a developing	Zakzouk SM,	J Otolaryngol	10	1997	King Saud University	Observational Studies	Riyadh	None	None	10 Ear & mastoid	Disorders with hearing impairment	Acquired hearing impairment

PMID	Title	Authors	Journal/Book	Month	Year	First author institution	Type of Publication	Region	Funding	COI	ICD11 Chapter	ICD11 Block	ICD11 Category
	country: Part II												
9536913	CT and MRI features of cavernous haemangioma of internal auditory canal	Omojola MF, al Hawashim NS, Zuwayed MA, al Ferayan A,	Br J Radiol	11	1997	King Abdulaziz Medical City	Observational Studies	Riyadh	None	None	02 Neoplasms	Benign neoplasms, except of lymphoid, haematopoietic, central nervous system or related tissues	Benign mesenchymal neoplasms
9578872	Acquired medial canal fibrosis	el-Sayed Y,	J Laryngol Otol	2	1998	King Saud University	Systematic review and Meta-Analysis	Riyadh	None	None	10 Ear & mastoid	Disorders of ear, not elsewhere classified	Diseases of the ear or mastoid process, unspecified
9617924	Bone conduction impairment in uncomplicated chronic suppurative otitis media	El-Sayed Y,	Am J Otolaryngol	6	1998	King Saud University	Observational Studies	Riyadh	None	None	10 Ear & mastoid	Diseases of middle ear or mastoid	Suppurative otitis media
9692639	Coital vertigo after ear surgery: when is sex safe?	Dawlatly EE,	Am J Otolaryngol	8	1998	Imam Abdulrahman Bin Faisal University	Observational Studies	Eastern Province	None	None	21 Symptoms, signs or clinical findings, not elsewhere classified	Symptoms, signs or clinical findings of the nervous system	Symptoms or signs involving the nervous system
9703081	Cosman ear: congenital auricular cleft between the fifth and sixth hillocks	Al-Qattan MM,	Plast Reconstr Surg	8	1998	King Saud University	Observational Studies	Riyadh	None	None	20 Developmental anomalies	Structural developmental anomalies primarily affecting one body system	Structural developmental anomalies of the ear

PMID	Title	Authors	Journal/Book	Month	Year	First author institution	Type of Publication	Region	Funding	COI	ICD11 Chapter	ICD11 Block	ICD11 Category
9949369	Merkel cell carcinoma of the pinna	Gangopadhyay K, Abuzeid MO,	Otolaryngol Head Neck Surg	2	1999	King Faisal Specialist Hospital and Research Centre	Observational Studies	Riyadh	None	None	02 Neoplasms	Malignant neoplasms, stated or presumed to be primary, of specified sites, except of lymphoid, haematopoietic, central nervous system or related tissues	Malignant neoplasms of skin
10442770	Iatrogenic incudostapedial joint dislocation in transcanal tympanoplasty	Attallah MS, Zakzouk SM,	Am J Otolaryngol	8	1999	King Saud University	Observational Studies	Riyadh	None	None	24 Factors influencing health status or contact with health services	Reasons for contact with the health services	Contact with health services for specific surgical interventions
10767919	Tympanosclerosis: review of literature and incidence among patients with middle-ear infection	Asiri S, Hasham A, al Anazy F, Zakzouk S, Banjar A,	J Laryngol Otol	12	1999	Prince Sultan Armed Forces Hospital	Systematic review and Meta-Analysis	Madinah	None	None	24 Factors influencing health status or contact with health services	Reasons for contact with the health services	Contact with health services for specific surgical interventions
10954517	Endolymphatic sac tumor and von Hippel-Lindau disease: imaging features	Ayadi K, Mahfoudh KB, Khannous M, Mnif J,	AJR Am J Roentgenol	9	2000	King Khalid Military City Hospital	Observational Studies	Eastern Province	None	None	02 Neoplasms	Malignant neoplasms, stated or presumed to be primary, of specified sites, except of lymphoid, haematopoietic, central nervous system or related tissues	Malignant neoplasms, stated or presumed to be primary, of specified sites, except of lymphoid, haematopoietic, central nervous system or related tissues

PMID	Title	Authors	Journal/Book	Month	Year	First author institution	Type of Publication	Region	Funding	COI	ICD11 Chapter	ICD11 Block	ICD11 Category
11360094	Allograftic and alloplastic auricular reconstruction	Habiballah JA, Bamousa A,	Saudi Med J	12	2000	Prince Sultan Military Medical City	Observational Studies	Riyadh	None	None	24 Factors influencing health status or contact with health services	Reasons for contact with the health services	Contact with health services for specific surgical interventions
11369953	Microbiology of chronic suppurative otitis media with cholesteatoma	Attallah MS,	Saudi Med J	10	2000	King Saud University	Observational Studies	Riyadh	None	None	10 Ear & mastoid	Diseases of middle ear or mastoid	Cholesteatoma of middle ear
11369955	A pilot study of the relationship between Down's syndrome and hearing loss	Kattan HA, Jarrar RF, Mahasin ZZ,	Saudi Med J	10	2000	King Faisal Specialist Hospital and Research Centre	Observational Studies	Riyadh	None	None	10 Ear & mastoid	Disorders with hearing impairment	Congenital hearing impairment
11376367	The pattern of hearing impairment among schoolboys in an Institute for deaf subjects	Abolfotouh MA, Al-Ghamdi SA,	Saudi Med J	9	2000	King Khalid University	Observational Studies	Asir	None	None	10 Ear & mastoid	Disorders with hearing impairment	Congenital hearing impairment
11418087	Occupational noise exposure and hearing loss of workers in two plants in eastern Saudi Arabia	Ahmed HO, Dennis JH, Badran O, Ismail M, Ballal SG, Ashoor A, Jerwood D,	Ann Occup Hyg	7	2001	Imam Abdulrahman Bin Faisal University	Observational Studies	Eastern Province	None	None	10 Ear & mastoid	Diseases of inner ear	Noise effects on inner ear
11463869	High-frequency (10-18 kHz) hearing thresholds: reliability, and effects of age and occupational	Ahmed HO, Dennis JH, Badran O, Ismail M, Ballal SG, Ashoor A, Jerwood D,	Occup Med (Lond)	6	2001	Imam Abdulrahman Bin Faisal University	Observational Studies	Eastern Province	None	None	10 Ear & mastoid	Diseases of inner ear	Noise effects on inner ear

PMID	Title	Authors	Journal/Book	Month	Year	First author institution	Type of Publication	Region	Funding	COI	ICD11 Chapter	ICD11 Block	ICD11 Category
	noise exposure												
11500698	Occupational hearing loss	Al-Otaibi ST,	Saudi Med J	6	2000	Dhahran Health Center	Systematic review and Meta-Analysis	Eastern Province	None	None	10 Ear & mastoid	Diseases of inner ear	Noise effects on inner ear
11702883	Extraosseous endolymphatic sac low-grade adenocarcinoma mimicking posterior fossa meningioma	Al-Anazi AR, Holliday W, Sheikh B, Gentili F,	J Neurosurg	11	2001	Imam Abdulrahman Bin Faisal University	Observational Studies	Eastern Province	None	None	02 Neoplasms	Malignant neoplasms, stated or presumed to be primary, of specified sites, except of lymphoid, haematopoietic, central nervous system or related tissues	Malignant neoplasms, stated or presumed to be primary, of specified sites, except of lymphoid, haematopoietic, central nervous system or related tissues
11794420	Sudden irreversible sensory-neural hearing loss in a patient with diabetes receiving amikacin as an antibiotic-heparin lock	Saxena AK, Panhotra BR, Naguib M,	Pharmacotherapy	1	2002	King Fahad Hospital	Observational Studies	Eastern Province	None	None	10 Ear & mastoid	Disorders with hearing impairment	Ototoxic hearing loss
11852123	Epidemiology of chronic suppurative otitis media among Saudi children--a comparative study of two decades	Zakzouk SM, Hajjaj MF,	Int J Pediatr Otorhinolaryngol	2	2002	King Abdulaziz University	Observational Studies	Makkah	None	None	10 Ear & mastoid	Diseases of middle ear or mastoid	Suppurative otitis media

PMID	Title	Authors	Journal/Book	Month	Year	First author institution	Type of Publication	Region	Funding	COI	ICD11 Chapter	ICD11 Block	ICD11 Category
11852124	Epidemiology of acute otitis media among Saudi children	Zakzouk SM, Jamal TS, Daghistani KJ,	Int J Pediatr Otorhinolaryngol	2	2002	King Abdulaziz University	Observational Studies	Makkah	King Abdulaziz City for science and Technology	None	10 Ear & mastoid	Diseases of middle ear or mastoid	Otitis media
11892124	Cochlear reimplantation	Handoussa A,	Adv Otorhinolaryngol	NA	2000	El-Maghraby Eye and Ear Hospital	Technical report	Makkah	None	None	24 Factors influencing health status or contact with health services	Reasons for contact with the health services	Presence of device, implants or grafts
11892157	Experience with cochlear implants in postmeningitic cases	al-Shaikh AH, Metwalli AA,	Adv Otorhinolaryngol	NA	2000	King Fahad Hospital	Observational Studies	Makkah	None	None	24 Factors influencing health status or contact with health services	Reasons for contact with the health services	Presence of device, implants or grafts
11892222	Fixation of intracochlear electrode at the cochleostomy	Handoussa A,	Adv Otorhinolaryngol	NA	2000	El-Maghraby Eye and Ear Hospital	Technical report	Makkah	None	None	24 Factors influencing health status or contact with health services	Reasons for contact with the health services	Presence of device, implants or grafts
11938423	Hearing impairment in low birth weight children	Daghistani KJ, Jamal TS, Zakzouk SM,	Saudi Med J	3	2002	King Abdulaziz University	Observational Studies	Makkah	King Abdulaziz City for science and Technology	None	10 Ear & mastoid	Disorders with hearing impairment	Hearing impairment (unspecified)

PMID	Title	Authors	Journal/Book	Month	Year	First author institution	Type of Publication	Region	Funding	COI	ICD11 Chapter	ICD11 Block	ICD11 Category
11953772	Cochlear implants in deaf children	Al-Shaikh AH, Zakzouk SM, Metwalli AA, Dasugi AA,	Saudi Med J	2	2002	King Fahad Hospital	Observational Studies	Makkah	None	None	24 Factors influencing health status or contact with health services	Reasons for contact with the health services	Presence of device, implants or grafts
12020909	Sensorineural hearing impaired children with NA causes: a comprehensive etiological study	Zakzouk SM, Al-Anazy F,	Int J Pediatr Otorhinolaryngol	2	2002	Security Forces Hospital	Observational Studies	Riyadh	None	None	10 Ear & mastoid	Disorders with hearing impairment	hearing impairment (unspecified)
12070553	Point prevalence of type B tympanogram in children	Zakzouk SM, AbdulJawad KA,	Saudi Med J	6	2002	King Saud University	Observational Studies	Riyadh	None	None	10 Ear & mastoid	Diseases of middle ear or mastoid	Otitis media
12437836	Consanguinity and hearing impairment in developing countries: a custom to be discouraged	Zakzouk S,	J Laryngol Otol	10	2002	Security Forces Hospital	Observational Studies	Riyadh	None	None	10 Ear & mastoid	Disorders with hearing impairment	Congenital hearing impairment
12487675	Conductive hearing loss: investigation of possible inner ear origin in three cases studies	Al Muhaimeed H, El Sayed Y, Rabah A, Al-Essa A,	J Laryngol Otol	11	2002	King Saud University	Observational Studies	Riyadh	None	None	10 Ear & mastoid	Disorders with hearing impairment	Acquired hearing impairment
12562108	Hearing loss in amphetamine users	Iqbal N,	J Psychoactive Drugs	10	2002	Al Amal Hospital	Observational Studies	Makkah	None	None	10 Ear & mastoid	Disorders with hearing impairment	Acquired hearing impairment
12590276	Hearing screening of neonates at risk	Maisoun AM, Zakzouk SM,	Saudi Med J	9	2003	Security Forces Hospital	Observational Studies	Riyadh	None	None	24 Factors influencing health status or contact with health services	Reasons for contact with the health services	Contact with health services for purposes of examination or investigation

PMID	Title	Authors	Journal/Book	Month	Year	First author institution	Type of Publication	Region	Funding	COI	ICD11 Chapter	ICD11 Block	ICD11 Category
12590277	Outcome of type-1 tympanoplasty	Fadl FA,	Saudi Med J	10	2003	King Fahad Specialist Hospital (Buraydah)	Observational Studies	Qassim	None	None	24 Factors influencing health status or contact with health services	Reasons for contact with the health services	Contact with health services for specific surgical interventions
12704497	Universal newborn hearing screening	Zakzouk SM,	Saudi Med J	12	2003	Security Forces Hospital	Systematic review and Meta-Analysis	Riyadh	None	None	24 Factors influencing health status or contact with health services	Reasons for contact with the health services	Contact with health services for purposes of examination or investigation
12883619	Angiolymphoid hyperplasia with eosinphilia presenting with postauricular swelling	Akram IS, Raza SS, Asrar L, Faizuddin M.	Saudi Med J	7	2003	Buraidah Central Hospital	Observational Studies	Qassim	None	None	02 Neoplasms	Benign neoplasms, except of lymphoid, haematopoietic, central nervous system or related tissues	Benign cutaneous neoplasms
14676710	An alternative approach for correction of Stahl's ear	Al-Qattan MM, Hashem FK,	Ann Plast Surg	1	2004	King Saud University	Observational Studies	Riyadh	None	None	24 Factors influencing health status or contact with health services	Reasons for contact with the health services	Contact with health services for specific surgical interventions
14758395	Ramsay Hunt syndrome in focal segmental glomerulosclerosis	Cheema KM, Kanu MK, Kutaiba M, Sohail M,	Saudi Med J	1	2004	Huraymala general hospital	Observational Studies	Riyadh	None	None	08 Diseases of the nervous system	Disorders of nerve root, plexus or peripheral nerves	Disorders of cranial nerves
15008297	Prospective study of hearing loss in schools for deaf children in Assir region, Saudi Arabia	Fageeh NA,	West Afr J Med	12	2003	King Khalid University	Observational Studies	Asir	None	None	10 Ear & mastoid	Disorders with hearing impairment	Congenital hearing impairment

PMID	Title	Authors	Journal/Book	Month	Year	First author institution	Type of Publication	Region	Funding	COI	ICD11 Chapter	ICD11 Block	ICD11 Category
15008312	Mastoid cells myiasis in a Saudi man: a case report	Al-Abidi AA, Bello C, Al-Ahmari M, Fawehinmi Y,	West Afr J Med	12	2003	Aseer Central Hospital	Observational Studies	Asir	None	None	01 Certain infectious or parasitic diseases	Parasitic diseases	Infestations by ectoparasites
15330390	The accuracy of self-reported high noise exposure level and hearing loss in a working population in Eastern Saudi Arabia	Ahmed HO, Dennis JH, Ballal SG,	Int J Hyg Environ Health	8	2004	United Arab Emirates University	Observational Studies	Eastern Province	None	None	10 Ear & mastoid	Diseases of inner ear	Noise effects on inner ear
15369211	Recoverable hearing loss with amphetamines and other drugs	Iqbal N,	J Psychoactive Drugs	6	2004	Al Amal Hospital	Observational Studies	Makkah	None	None	10 Ear & mastoid	Disorders with hearing impairment	Acquired hearing impairment
15646162	Middle ear cholesteatoma: characteristic CT findings in 64 patients	Gaurano JL, Joharjy IA,	Ann Saudi Med	12	2004	King Saud University	Observational Studies	Riyadh	None	None	10 Ear & mastoid	Diseases of middle ear or mastoid	Cholesteatoma of middle ear
15780236	An alternative approach for correction of constricted ears of moderate severity	Al-Qattan MM,	J Plast Reconstr Aesthet Surg	4	2005	King Saud University	Observational Studies	Riyadh	None	None	24 Factors influencing health status or contact with health services	Reasons for contact with the health services	Contact with health services for specific surgical interventions
15885338	Neonatal hearing screening with transient evoked otoacoustic emissions in Western Saudi Arabia	Habib HS, Abdelgaffar H,	Int J Pediatr Otorhinolaryngol	6	2005	King Abdulaziz University	Observational Studies	Makkah	None	None	24 Factors influencing health status or contact with health services	Reasons for contact with the health services	Contact with health services for purposes of examination or investigation

PMID	Title	Authors	Journal/Book	Month	Year	First author institution	Type of Publication	Region	Funding	COI	ICD11 Chapter	ICD11 Block	ICD11 Category
15903215	Ruptured petrous carotid pseudoaneurysm complicating malignant otitis externa	Telmesani LM,	J Otolaryngol	8	2004	Imam Abdulrahman Bin Faisal University	Observational Studies	Eastern Province	None	None	10 Ear & mastoid	Diseases of External Ear	Infectious diseases of external ear
15951891	Do mobile phones cause hearing and vision complaints? A preliminary report	Meo SA, Al-Drees AM,	Saudi Med J	5	2005	King Saud University	Observational Studies	Riyadh	None	None	10 Ear & mastoid	Disorders with hearing impairment	Acquired hearing impairment
16822553	Iatrogenic cholesteatoma in children with OME in a training program	Al Anazy FH,	Int J Pediatr Otorhinolaryngol	10	2006	King Saud University	Observational Studies	Riyadh	None	None	10 Ear & mastoid	Diseases of middle ear or mastoid	Cholesteatoma of middle ear
17143362	Tympanoplasty type-1. Endaural or postaural approach. Should the patient decide?	Ahmed LA, Raza SS,	Saudi Med J	12	2006	Buraidah Central Hospital	Observational Studies	Qassim	None	None	24 Factors influencing health status or contact with health services	Reasons for contact with the health services	Contact with health services for specific surgical interventions
17293301	The prevalence of Stahl's ear deformity in Saudi Arabia	Al-Zahrani K, Al-Humsi T, Hassanain J, Al-Qattan MM,	J Plast Reconstr Aesthet Surg	7	2007	King Saud University	Observational Studies	Riyadh	None	None	20 Developmental anomalies	Structural developmental anomalies primarily affecting one body system	Structural developmental anomalies of the ear
17339784	Cochlear implantation in deaf children	Makhdoum MJ, Snik FM, van de Broek P,	Ann Saudi Med	11	1997	Alnour Specialist Hospital	Systematic review and Meta-Analysis	Makkah	None	None	24 Factors influencing health status or contact with health services	Reasons for contact with the health services	Presence of device, implants or grafts

PMID	Title	Authors	Journal/Book	Month	Year	First author institution	Type of Publication	Region	Funding	COI	ICD11 Chapter	ICD11 Block	ICD11 Category
26890426	The activities-specific balance confidence scale and berg balance scale: Reliability and validity in Arabic-speaking vestibular patients	Alghwiri AA, Alghadir AH, Al-Momani MO, Whitney SL.	J Vestib Res	6	2015	King Saud University	Observational Studies	Riyadh	None	None	21 Symptoms, signs or clinical findings, not elsewhere classified	Symptoms, signs or clinical findings of the nervous system	Symptoms or signs involving the nervous system
17372426	Prevalence and etiology of childhood sensorineural hearing loss in Riyadh	El Sayed Y, Zakzouk S,	Ann Saudi Med	5	1996	King Saud University	Observational Studies	Riyadh	None	None	10 Ear & mastoid	Disorders with hearing impairment	hearing impairment (unspecified)
17372521	Toxoplasmosis and congenital sensorineural hearing loss in Saudi Arabia	Al-Amari O, Kameswaran M,	Ann Saudi Med	7	1996	King Khalid University	Observational Studies	Asir	None	None	10 Ear & mastoid	Disorders with hearing impairment	congenital hearing impairment
17429245	Sensorineural hearing loss in homozygous sickle cell disease in Qatif, Saudi Arabia	Al-Dabbous IA, Al Jam'a AH, Obeja SK, Murugan AN, Hammad HA,	Ann Saudi Med	11	1996	Qatif Central Hospital	Observational Studies	Eastern Province	None	None	10 Ear & mastoid	Disorders with hearing impairment	Acquired hearing impairment
17488924	A human case of otoacariasis involving a histiostomatid mite (Acari: Histiostomatidae)	Al-Arfaj AM, Mullen GR, Rashad R, Abdel-Hameed A, OConnor BM, Alkhalife IS, Dute RR,	Am J Trop Med Hyg	5	2007	King Saud University	Observational Studies	Riyadh	None	None	01 Certain infectious or parasitic diseases	Parasitic diseases	Infestations by ectoparasites
17587028	Bacterial meningitis and hearing impairment:	Zakzouk SM, El-Sayed Y,	Ann Saudi Med	9	1992	King Saud University	Observational Studies	Riyadh	None	None	10 Ear & mastoid	Disorders with hearing impairment	Acquired hearing impairment

PMID	Title	Authors	Journal/Book	Month	Year	First author institution	Type of Publication	Region	Funding	COI	ICD11 Chapter	ICD11 Block	ICD11 Category
	A prospective study												
17590599	Hearing loss and other ear problems among schoolboys in Abha, Saudi Arabia	Abolfotouh MA, Ghieth MM, Badawi IA,	Ann Saudi Med	7	1995	King Khalid University	Observational Studies	Asir	None	None	10 Ear & mastoid	Disorders with hearing impairment	Hearing impairment (unspecified)
17590727	Consanguinity and hereditary hearing impairment among Saudi population	Zakzouk S, El-Sayed Y, Bafaqeeh SA,	Ann Saudi Med	9	1993	King Saud University	Observational Studies	Riyadh	None	None	10 Ear & mastoid	Disorders with hearing impairment	Hereditary hearing loss
17676220	Mobile phone induced sensorineural hearing loss	Al-Dousary SH,	Saudi Med J	8	2007	King Saud University	Observational Studies	Riyadh	None	None	10 Ear & mastoid	Disorders with hearing impairment	Acquired hearing impairment
18454228	Stapedectomy in tympanosclerosis, hearing results	Al-Qahtani M, Hagr A,	Saudi Med J	5	2008	Prince Sultan Military Medical City	Observational Studies	Riyadh	None	None	24 Factors influencing health status or contact with health services	Reasons for contact with the health services	Contact with health services for specific surgical interventions
18521478	Oval window perilymph fistula caused by accidental stapedectomy during ear toilet	Alharethy SE,	Saudi Med J	6	2008	King Saud University	Observational Studies	Riyadh	None	None	24 Factors influencing health status or contact with health services	Reasons for contact with the health services	Contact with health services for specific surgical interventions
18701883	Syndromic congenital sensorineural deafness, microtia and microdontia resulting from a novel homoallelic mutation in fibroblast	Alsmadi O, Meyer BF, Alkuraya F, Wakil S, Alkayal F, Al-Saud H, Ramzan K, Al-Sayed M,	Eur J Hum Genet	1	2009	King Faisal Specialist Hospital and Research Centre	Observational Studies	Riyadh	None	None	10 Ear & mastoid	Disorders with hearing impairment	Congenital hearing impairment

PMID	Title	Authors	Journal/Book	Month	Year	First author institution	Type of Publication	Region	Funding	COI	ICD11 Chapter	ICD11 Block	ICD11 Category
	growth factor 3 (FGF3)												
19370541	The transmeatal approach: a new technique in cochlear and middle ear implants	Taibah K,	Cochlear Implants Int	12	2009	King Faisal Specialist Hospital and Research Centre	Observational Studies	Riyadh	None	None	24 Factors influencing health status or contact with health services	Reasons for contact with the health services	Presence of device, implants or grafts
19692896	Z-plasty for Tanzer type IIb constricted ears	Al-Qattan MM, Al-Omawi M,	Ann Plast Surg	9	2009	King Saud University	Observational Studies	Riyadh	None	None	24 Factors influencing health status or contact with health services	Reasons for contact with the health services	Contact with health services for specific surgical interventions
19775492	Cochlear implantation at King Abdulaziz University Hospital, Riyadh, Saudi Arabia: a 12-year experience	Al-Muhaimeed HS, Al-Anazy F, Attallah MS, Hamed O,	J Laryngol Otol	11	2009	King Saud University	Observational Studies	Riyadh	None	None	24 Factors influencing health status or contact with health services	Reasons for contact with the health services	Presence of device, implants or grafts
19929407	Spectrum of GJB2 mutations in a cohort of nonsyndromic hearing loss cases from the Kingdom of Saudi Arabia	Al-Qahtani MH, Baghlab I, Chaudhary AG, Abuzenadah AM, Bamanie A, Daghistani KJ, Safieh M, Fida L, Dallol A,	Genet Test Mol Biomarkers	2	2010	King Abdulaziz University	Observational Studies	Makkah	King Abdulaziz University | Ministry of Higher Education	None	10 Ear & mastoid	Disorders with hearing impairment	Congenital hearing impairment

PMID	Title	Authors	Journal/Book	Month	Year	First author institution	Type of Publication	Region	Funding	COI	ICD11 Chapter	ICD11 Block	ICD11 Category
20074818	Correlation between NRT measurement level and behavioral levels in pediatrics cochlear implant patients	Muhaimeed HA, Anazy FA, Hamed O, Shubair E,	Int J Pediatr Otorhinolaryngol	4	2010	King Saud University	Observational Studies	Riyadh	None	None	24 Factors influencing health status or contact with health services	Reasons for contact with the health services	Presence of device, implants or grafts
20714689	A preliminary study of endoscopic acoustic stapedial reflex in chronic otitis media	Al-Qahtani MM, Hagr AA,	Saudi Med J	8	2010	Prince Sultan Military Medical City	Observational Studies	Riyadh	King Saud University	None	10 Ear & mastoid	Diseases of middle ear or mastoid	Otitis media
20880417	Auditory and vestibular manifestations of Vogt-Koyanagi-Harada disease	Al Dousary S,	J Laryngol Otol	2	2011	King Saud University	Observational Studies	Riyadh	None	None	14 Diseases of the skin	Skin disorders involving specific cutaneous structures	Disorders of skin colour
21048654	Mixed vestibular schwannoma and meningioma without neurofibromatosis	Al-Anazi AH, Al-Luwimi IM, Shawarby MA, Mertol T,	Neurosciences (Riyadh)	10	2009	King Faisal University rem	Observational Studies	Eastern Province	None	None	02 Neoplasms	Neoplasms of central nervous system or related structures	Primary neoplasm of spinal cord, cranial nerves, paraspinal nerves or remaining parts of central nervous system
21331786	The effect of cochlear implantation and post-operative rehabilitation on acoustic voice analysis in post-lingual	Hassan SM, Malki KH, Mesallam TA, Farahat M, Bukhari M, Murry T,	Eur Arch Otorhinolaryngol	10	2011	King Saud University	Observational Studies	Riyadh	None	None	24 Factors influencing health status or contact with health services	Reasons for contact with the health services	Presence of device, implants or grafts

PMID	Title	Authors	Journal/Book	Month	Year	First author institution	Type of Publication	Region	Funding	COI	ICD11 Chapter	ICD11 Block	ICD11 Category
	hearing impaired adults												
21372348	Effect of interferon treatment on hearing of patients with chronic hepatitis C	Hagr A, Jamjoom D, Sanai FM, Al Hamoudi W, Abdo AA, Al-Arfaj A,	Saudi J Gastroenterol	3	2011	King Saud University	Observational Studies	Riyadh	King Saud University	None	10 Ear & mastoid	Disorders with hearing impairment	Ototoxic hearing loss
21384063	Prevalence of hearing loss among Saudi type 2 diabetic patients	Bamanie AH, Al-Noury KI,	Saudi Med J	1	2011	King Abdulaziz University	Observational Studies	Makkah	None	None	05 Endocrine, nutritional or metabolic diseases	Endocrine diseases	Diabetes mellitus
21400256	Computed tomography and magnetic resonance imaging findings before and after treatment of patients with malignant external otitis	Al-Noury K, Lotfy A,	Eur Arch Otorhinolaryngol	12	2011	King Abdulaziz University	Observational Studies	Makkah	None	None	10 Ear & mastoid	Diseases of External Ear	Infectious diseases of external ear
21475438	BAHA: Bone-Anchored Hearing Aid	Hagr A,	Int J Health Sci (Qassim)	7	2007	King Saud University	Systematic review and Meta-Analysis	Riyadh	None	None	24 Factors influencing health status or contact with health services	Reasons for contact with the health services	Presence of device, implants or grafts
21475458	Intra-parenchyma Pneumocephalus: a Complication of Cochlear Implant	Hagr A, Bance M,	Int J Health Sci (Qassim)	1	2007	King Saud University	Observational Studies	Riyadh	None	None	08 Diseases of the nervous system	Dissociative neurological symptom disorder	Other specified diseases of the nervous system

PMID	Title	Authors	Journal/Book	Month	Year	First author institution	Type of Publication	Region	Funding	COI	ICD11 Chapter	ICD11 Block	ICD11 Category
21495043	Distortion product otoacoustic emission for the screening of cochlear damage in children treated with cisplatin	Al-Noury K,	Laryngoscope	5	2011	King Abdulaziz University	Observational Studies	Makkah	None	None	24 Factors influencing health status or contact with health services	Reasons for contact with the health services	Presence of device, implants or grafts
21509237	Hearing Improvement after Partial Labyrinthectomy: Resection of petrous apex cholesterol granuloma	Hagr AA,	Sultan Qaboos Univ Med J	7	2010	King Saud University	Observational Studies	Riyadh	None	None	10 Ear & mastoid	Disorders with hearing impairment	Hearing impairment (unspecified)
21726435	A comprehensive introduction to the genetic basis of non-syndromic hearing loss in the Saudi Arabian population	Imtiaz F, Taibah K, Ramzan K, Bin-Khamis G, Kennedy S, Al-Mubarak B, Trabzuni D, Allam R, Al-Mostafa A, Sogaty S, Al-Shaikh AH, Bamukhayyar SS, Meyer BF, Al-Owain M,	BMC Med Genet	7	2011	King Faisal Specialist Hospital and Research Centre	Observational Studies	Riyadh	King Faisal Specialist Hospital and Research Centre | King Salman Centre for Disability Research | Dubai Harvard Foundation for medical	None	10 Ear & mastoid	Disorders with hearing impairment	Congenital hearing impairment

PMID	Title	Authors	Journal/Book	Month	Year	First author institution	Type of Publication	Region	Funding<br><br>research for a research fellowship	COI	ICD11 Chapter	ICD11 Block	ICD11 Category
21756623	Assessment of auditory performance in young children with cochlear implants	Al-Muhaimeed H,	Cochlear Implants Int	6	2010	King Saud University	Observational Studies	Riyadh	None	None	24 Factors influencing health status or contact with health services	Reasons for contact with the health services	Presence of device, implants or grafts
21777530	Brainstem auditory evoked potentials and electrocochleographic findings in patients with idiopathic sudden sensorineural hearing loss	Habib SS, Husain A, Omar SA, Al Drees AM,	J Coll Physicians Surg Pak	7	2011	King Saud University	Observational Studies	Riyadh	None	None	10 Ear & mastoid	Disorders with hearing impairment	Sudden idiopathic hearing loss
21777545	Cochlear implantation in fractured inner ears	Hagr A,	J Otolaryngol Head Neck Surg	8	2011	King Saud University	Observational Studies	Riyadh	None	None	24 Factors influencing health status or contact with health services	Reasons for contact with the health services	Presence of device, implants or grafts

PMID	Title	Authors	Journal/Book	Month	Year	First author institution	Type of Publication	Region	Funding	COI	ICD11 Chapter	ICD11 Block	ICD11 Category
22018730	Otorhinolaryngological manifestations of sickle cell disease	Abou-Elhamd KE,	Int J Pediatr Otorhinolaryngol	1	2012	King Faisal University	Systematic review and Meta-Analysis	Eastern Province	None	None	10 Ear & mastoid	Disorders with hearing impairment	Acquired hearing impairment
22082866	The effect of cochlear implantation on nasalance of speech in postlingually hearing-impaired adults	Hassan SM, Malki KH, Mesallam TA, Farahat M, Bukhari M, Murry T,	J Voice	9	2012	King Saud University	Observational Studies	Riyadh	None	None	24 Factors influencing health status or contact with health services	Reasons for contact with the health services	Presence of device, implants or grafts
22087379	Intra-Operative Neural Response Telemetry and Acoustic Reflex Assessment using an Advance-In-Stylet Technique and Modiolus-Hugging: A prospective cohort study	Hagr A,	Sultan Qaboos Univ Med J	8	2011	King Saud University	Observational Studies	Riyadh	Prince Sultan Research Chair for hearing disability	None	24 Factors influencing health status or contact with health services	Reasons for contact with the health services	Presence of device, implants or grafts
22159383	Impact of tinnitus on the quality of life among Saudi patients	Alsanosi AA,	Saudi Med J	10	2011	King Saud University	Observational Studies	Riyadh	None	None	21 Symptoms, signs or clinical findings, not elsewhere classified	Symptoms, signs or clinical findings of ear or mastoid process	Symptoms or signs involving the ear or mastoid process
22266447	Chronic suppurative otitis media with intracranial complication	Aldakhail AA,	Neurosciences (Riyadh)	10	2006	King Saud Medical City	Observational Studies	Riyadh	None	None	10 Ear & mastoid	Diseases of middle ear or mastoid	Suppurative otitis media

PMID	Title	Authors	Journal/Book	Month	Year	First author institution	Type of Publication	Region	Funding	COI	ICD11 Chapter	ICD11 Block	ICD11 Category
22420390	An otalgia pain syndrome, the anterolateral tip of the mastoid syndrome (ATOMS): diagnosis and response to treatment	Hagr A, Bance M,	J Otolaryngol Head Neck Surg	10	2011	King Saud University	Observational Studies	Riyadh	None	None	10 Ear & mastoid	Disorders of ear, not elsewhere classified	Otalgia or effusion of ear
22465888	Adaptation of the dizziness handicap inventory for use in the Arab population	Alsanosi AA,	Neurosciences (Riyadh)	4	2012	King Saud University	Observational Studies	Riyadh	None	None	21 Symptoms, signs or clinical findings, not elsewhere classified	Symptoms, signs or clinical findings of the nervous system	Symptoms or signs involving the nervous system
22499343	A recessive form of Marshall syndrome is caused by a mutation in the COL11A1 gene	Khalifa O, Imtiaz F, Allam R, Al-Hassnan Z, Al-Hemidan A, Al-Mane K, Abuharb G, Balobaid A, Sakati N, Hyland J, Al-Owain M,	J Med Genet	4	2012	King Faisal Specialist Hospital and Research Centre	Observational Studies	Riyadh	None	None	20 Developmental anomalies	Multiple developmental anomalies or syndromes	Syndromes with skin or mucosal anomalies as a major feature
22664903	Fungal malignant otitis externa: pitfalls, diagnosis, and treatment	Tarazi AE, Al-Tawfiq JA, Abdi RF,	Otol Neurotol	7	2012	Saudi Aramco Medical Services	Observational Studies	Eastern Province	None	None	10 Ear & mastoid	Diseases of External Ear	Infectious diseases of external ear
22686506	Microcornea with myopic chorioretinal atrophy, telecanthus and posteriorly-rotated ears: a distinct clinical syndrome	Khan AO,	Ophthalmic Genet	12	2012	King Khaled Eye Specialist Hospital	Observational Studies	Riyadh	None	None	10 Ear & mastoid	Disorders with hearing impairment	Hereditary hearing loss

PMID	Title	Authors	Journal/Book	Month	Year	First author institution	Type of Publication	Region	Funding	COI	ICD11 Chapter	ICD11 Block	ICD11 Category
22772935	Ramsay Hunt syndrome with multiple cranial neuropathies in a liver transplant recipient	Babtain FA, Bhatia HS, Assiri AH,	Neurosciences (Riyadh)	7	2012	King Khalid University	Observational Studies	Asir	None	None	08 Diseases of the nervous system	Disorders of nerve root, plexus or peripheral nerves	Disorders of cranial nerves
22876113	USH1G with unique retinal findings caused by a novel truncating mutation identified by genome-wide linkage analysis	Imtiaz F, Taibah K, Bin-Khamis G, Kennedy S, Hemidan A, Al-Qahtani F, Tabbara K, Al Mubarak B, Ramzan K, Meyer BF, Al-Owain M,	Mol Vis	7	2012	King Faisal Specialist Hospital and Research Centre	Observational Studies	Riyadh	King Faisal Specialist Hospital and Research Centre | King Salman Centre for Disability Research | Dubai Harvard Foundation for medical research for a research fellowship	None	20 Developmental anomalies	Multiple developmental anomalies or syndromes	Syndromic genetic deafness

PMID	Title	Authors	Journal/Book	Month	Year	First author institution	Type of Publication	Region	Funding	COI	ICD11 Chapter	ICD11 Block	ICD11 Category
22921777	Hearing impairments among Saudi preschool children	Al-Rowaily MA, AlFayez AI, AlJomiey MS, AlBadr AM, Abolfotouh MA,	Int J Pediatr Otorhinolaryngol	11	2012	King Saud bin Abdulaziz University for Health Sciences	Observational Studies	Riyadh	None	None	10 Ear & mastoid	Disorders with hearing impairment	Acquired hearing impairment
22950449	Partial chromosome 7 duplication with a phenotype mimicking the HOXA1 spectrum disorder	Abu-Amero KK, Kondkar AA, Salih MA, Alorainy IA, Khan AO, Oystreck DT, Bosley TM,	Ophthalmic Genet	3	2013	King Saud University	Observational Studies	Riyadh	None	None	09 Diseases of the visual system	Strabismus or ocular motility disorders	Disorders of extraocular muscles
23008530	Middle ear effusion in children: review of recent literature	Ashoor A,	J Family Community Med	1	1994	King Faisal University	Systematic review and Meta-Analysis	Eastern Province	None	None	10 Ear & mastoid	Disorders of ear, not elsewhere classified	Otalgia or effusion of ear
23012034	Hearing impairment and hypertension among long distance bus drivers	Abdelmoneim I,	J Family Community Med	9	2003	King Khalid University	Observational Studies	Asir	None	None	10 Ear & mastoid	Disorders with hearing impairment	Acquired hearing impairment
23020865	Influenza A (H1N1): a rare cause of deafness in two children	Alsanosi AA,	J Laryngol Otol	12	2012	King Saud University	Observational Studies	Riyadh	None	None	10 Ear & mastoid	Disorders with hearing impairment	Acquired hearing impairment
23150283	Heterogeneity in phenotype of usher-congenital hyperinsulinism syndrome: hearing loss, retinitis pigmentosa, and hyperinsulinemic hypoglycemia	Al Mutair AN, Brusgaard K, Bin-Abbas B, Hussain K, Felimban N, Al Shaikh A, Christesen HT,	Diabetes Care	3	2013	King Abdulaziz Medical City	Observational Studies	Riyadh	King Abdullah International Medical Research Center	None	20 Developmental anomalies	Multiple developmental anomalies or syndromes	Syndromic genetic deafness

PMID	Title	Authors	Journal/Book	Month	Year	First author institution	Type of Publication	Region	Funding	COI	ICD11 Chapter	ICD11 Block	ICD11 Category
	ranging from severe to mild with conversion to diabetes												
23295314	Labyrinthotomy or vestibulotomy in anatomic and congenital variations of the oval window and facial nerve	Al-Mazrou KA, Bayazit YA,	ORL J Otorhinolaryngol Relat Spec	1	2012	King Saud University	Observational Studies	Riyadh	None	None	24 Factors influencing health status or contact with health services	Reasons for contact with the health services	Contact with health services for specific surgical interventions
23331774	An update on vestibular physical therapy	Alghadir AH, Iqbal ZA, Whitney SL,	J Chin Med Assoc	1	2013	King Saud University	Systematic review and Meta-Analysis	Riyadh	None	None	21 Symptoms, signs or clinical findings, not elsewhere classified	Symptoms, signs or clinical findings of the nervous system	Symptoms or signs involving the nervous system
23396459	The acute auditory effects of exposure for 60 minutes to mobile`s electromagnetic field	Alsanosi AA, Al-Momani MO, Hagr AA, Almomani FM, Shami IM, Al-Habeeb SF.	Saudi Med J	2	2013	King Saud University	Experimental studies	Riyadh	King Saud University	None	10 Ear & mastoid	Disorders with hearing impairment	Hearing impairment (unspecified)
23510777	Homozygosity mapping identifies a novel GIPC3 mutation causing congenital nonsyndromic hearing loss in a Saudi family	Ramzan K, Al-Owain M, Allam R, Berhan A, Abuharb G, Taibah K, Imtiaz F,	Gene	5	2013	King Faisal Specialist Hospital and Research Centre	Observational Studies	Riyadh	None	None	10 Ear & mastoid	Disorders with hearing impairment	Congenital hearing impairment

PMID	Title	Authors	Journal/Book	Month	Year	First author institution	Type of Publication	Region	Funding	COI	ICD11 Chapter	ICD11 Block	ICD11 Category
23632871	Syndromes of hearing loss associated with visual loss	Abou-Elhamd KA, ElToukhy HM, Al-Wadaani FA,	Eur Arch Otorhinolaryngol	4	2014	King Faisal University	Systematic review and Meta-Analysis	Eastern Province	None	None	10 Ear & mastoid	Disorders with hearing impairment	Congenital hearing impairment
23730574	Trans-aditus approach: an alternative technique for cochlear implantation	Al Sanosi A,	Indian J Otolaryngol Head Neck Surg	6	2012	King Saud University	Observational Studies	Riyadh	None	None	24 Factors influencing health status or contact with health services	Reasons for contact with the health services	Contact with health services for specific surgical interventions
23730578	Virtual reality simulation in ear microsurgery: a pilot study	Al-Noury K,	Indian J Otolaryngol Head Neck Surg	8	2011	King Abdulaziz University	Observational Studies	Makkah	King Abdulaziz University	None	24 Factors influencing health status or contact with health services	Reasons for contact with the health services	Contact with health services for specific surgical interventions
24359979	The effect of age at cochlear implantation outcomes in Saudi children	AlSanosi A, Hassan SM,	Int J Pediatr Otorhinolaryngol	2	2014	King Saud University	Observational Studies	Riyadh	None	None	24 Factors influencing health status or contact with health services	Reasons for contact with the health services	Presence of device, implants or grafts
24622029	Cochlear implant device activation and programming: 5 days postimplantation	Alsabellha RM, Hagr A, Al-Momani MO, Garadat SN,	Otol Neurotol	4	2014	King Saud University	Experimental studies	Riyadh	None	None	24 Factors influencing health status or contact with health services	Reasons for contact with the health services	Presence of device, implants or grafts
24627078	Is type 1 diabetes mellitus a cause for subtle hearing loss in pediatric patients?	ALDajani N, ALkurdi A, ALMutair A, ALdraiwesh A, ALMazrou KA,	Eur Arch Otorhinolaryngol	3	2015	King Abdulaziz Medical City	Observational Studies	Riyadh	None	None	10 Ear & mastoid	Disorders with hearing impairment	hearing impairment (unspecified)

PMID	Title	Authors	Journal/Book	Month	Year	First author institution	Type of Publication	Region	Funding	COI	ICD11 Chapter	ICD11 Block	ICD11 Category
24658560	Cochlear implant and thiamine-responsive megaloblastic anemia syndrome	Hagr AA,	Ann Saudi Med	1	2014	King Saud University	Observational Studies	Riyadh	None	None	24 Factors influencing health status or contact with health services	Reasons for contact with the health services	Presence of device, implants or grafts
24741331	Keratitis-ichthyosis-deafness syndrome: first affected family reported in the Middle East	Al Fahaad H,	Int Med Case Rep J	3	2014	Najran University	Observational Studies	Najran	None	None	20 Developmental anomalies	Multiple developmental anomalies or syndromes	Syndromes with skin or mucosal anomalies as a major feature
24768815	ILDR1: Novel mutation and a rare cause of congenital deafness in the Saudi Arabian population	Ramzan K, Taibah K, Tahir AI, Al-Tassan N, Berhan A, Khater AM, Al-Hazzaa SA, Al-Owain M, Imtiaz F,	Eur J Med Genet	5	2014	King Faisal Specialist Hospital and Research Centre	Observational Studies	Riyadh	King Faisal Specialist Hospital and Research Centre | King Abdulaziz City for Science and Technology	None	10 Ear & mastoid	Disorders with hearing impairment	Congenital hearing impairment
24806347	Attitudes of Saudi parents with a deaf child towards prenatal diagnosis and termination of pregnancy	Alsulaiman A, Mousa A, Kondkar AA, Abu-Amero KK,	Prenat Diagn	2	2014	King Faisal Specialist Hospital and Research Centre	Observational Studies	Riyadh	King Faisal Specialist Hospital and Research	None	10 Ear & mastoid	Disorders with hearing impairment	Congenital hearing impairment

PMID	Title	Authors	Journal/Book	Month	Year	First author institution	Type of Publication	Region	Funding Center	COI	ICD11 Chapter	ICD11 Block	ICD11 Category
24894787	Cochlear implant for bilateral profound sensorineural hearing loss in an adolescent with sickle cell anemia	Almuhawas FA, Hagr AA,	Ann Saudi Med	3	2014	King Saud University	Observational Studies	Riyadh	None	None	24 Factors influencing health status or contact with health services	Reasons for contact with the health services	Presence of device, implants or grafts
25073711	Marshall syndrome: further evidence of a distinct phenotypic entity and report of new findings	Khalifa O, Imtiaz F, Ramzan K, Allam R, Hemidan AA, Faqeih E, Abuharb G, Balobaid A, Sakati N, Owain MA,	Am J Med Genet A	10	2014	King Faisal Specialist Hospital and Research Centre	Observational Studies	Riyadh	None	None	20 Developmental anomalies	Multiple developmental anomalies or syndromes	Syndromes with skin or mucosal anomalies as a major feature
25107659	A prospective, observational, epidemiological evaluation of the aetiology and antimicrobial susceptibility of acute otitis media in Saudi children younger than 5years of age	Al-Mazrou KA, Shibl AM, Kandeil W, Pir√ßon JY, Marano C,	J Epidemiol Glob Health	4	2014	King Saud bin Abdulaziz University for Health Sciences	Observational Studies	Riyadh	Glaxo Smith Kline Biologicals SA	K.A. M. has received an institutional grant from Glaxo Smith Kline group of companies.	10 Ear & mastoid	Diseases of middle ear or mastoid	Otitis media

PMID	Title	Authors	Journal/Book	Month	Year	First author institution	Type of Publication	Region	Funding	COI	ICD11 Chapter	ICD11 Block	ICD11 Category
25123111	"Neuroendocrine adenoma of the middle ear with the history of otitis media and carcinoma of the cheek: a case report"	Almuhanna K,	BMC Res Notes	8	2014	Prince Sultan Military Medical City	Observational Studies	Riyadh	None	None	02 Neoplasms	Benign neoplasms, except of lymphoid, haematopoietic, central nervous system or related tissues	Benign neoplasm of middle ear, respiratory or intrathoracic organs
25312238	Pediatric hearing loss: common causes, diagnosis and therapeutic approach	Alzahrani M, Tabet P, Saliba I,	Minerva Pediatr	2	2015	King Fahad Specialist Hospital	Observational Studies	Eastern Province	None	None	10 Ear & mastoid	Disorders with hearing impairment	Congenital hearing impairment
25500318	Subscales of the vestibular activities and participation questionnaire could be applied across cultures	Mueller M, Whitney SL, Alghwiri A, Alshebber K, Strobl R, Alghadir A, Al-momani MO, Furman JM, Grill E,	J Clin Epidemiol	2	2015	King Saud University	Observational Studies	Riyadh	German Federal Ministry of Education and Research	None	21 Symptoms, signs or clinical findings, not elsewhere classified	Symptoms, signs or clinical findings of the nervous system	Symptoms or signs involving the nervous system
25634774	Feasibility of one-day activation in cochlear implant recipients	Hagr A, Garadat SN, Al-Momani M, Alsabellha RM, Almuhawas FA,	Int J Audiol	5	2015	King Saud University	Observational Studies	Riyadh	King Saud University	None	24 Factors influencing health status or contact with health services	Reasons for contact with the health services	Presence of device, implants or grafts
25682901	Variable expression pattern in Donnai-Barrow syndrome: Report of two novel LRP2 mutations and review of the literature	Khalifa O, Al-Sahlawi Z, Imtiaz F, Ramzan K, Allam R, Al-Mostafa A, Abdel-Fattah M, Abuharb G, Nester M, Verloes A, Al-Zaidan H,	Eur J Med Genet	2	2015	King Faisal Specialist Hospital and Research Centre	Observational Studies	Riyadh	None	None	20 Developmental anomalies	Multiple developmental anomalies or syndromes	Syndromes with multiple structural anomalies, without predominant body system involvement

PMID	Title	Authors	Journal/Book	Month	Year	First author institution	Type of Publication	Region	Funding	COI	ICD11 Chapter	ICD11 Block	ICD11 Category
25746517	Effect of cochlear implant electrode array design on auditory nerve and behavioral response in children	Telmesani LM, Said NM,	Int J Pediatr Otorhinolaryngol	5	2015	Imam Abdulrahman Bin Faisal University	Observational Studies	Eastern Province	None	None	24 Factors influencing health status or contact with health services	Reasons for contact with the health services	Presence of device, implants or grafts
25780352	Prevalence and risk factors of Otitis Media with effusion in school children in Qassim Region of Saudi Arabia	Humaid AH, Ashraf AH, Masood KA, Nuha AH, Saleh AD, Awadh AM,	Int J Health Sci (Qassim)	10	2014	Qassim university	Observational Studies	Qassim	None	None	10 Ear & mastoid	Diseases of middle ear or mastoid	Otitis media
25852797	Severe Klippel-Feil syndrome with Mondini malformation of inner ear	Alaqeel AA,	Pan Afr Med J	11	2014	King Fahad Medical City	Observational Studies	Riyadh	None	None	20 Developmental anomalies	Structural developmental anomalies primarily affecting one body system	Structural developmental anomalies of the skeleton
25956234	Exome analysis identified a novel missense mutation in the CLPP gene in a consanguineous Saudi family expanding the clinical spectrum of Perrault Syndrome type-3	Ahmed S, Jelani M, Alrayes N, Mohamoud HS, Almramhi MM, Anshasi W, Ahmed NA, Wang J, Nasir J, Al-Aama JY,	J Neurol Sci	5	2015	King Abdulaziz University	Observational Studies	Makkah	None	None	20 Developmental anomalies	Multiple developmental anomalies or syndromes	Syndromic genetic deafness
26177750	Simultaneous bilateral cochlear implantation	Alsanosi AA,	J Laryngol Otol	7	2015	King Saud University	Observational Studies	Riyadh	None	None	20 Developmental anomalies	Multiple developmental anomalies or syndromes	Syndromic genetic deafness

PMID	Title	Authors	Journal/Book	Month	Year	First author institution	Type of Publication	Region	Funding	COI	ICD11 Chapter	ICD11 Block	ICD11 Category
	in a five-month-old child with Usher syndrome												
26527196	Evaluation of hearing among kindergarten children in Jazan (Kingdom of Saudi Arabia)	Alharbi FA, Ahmed MR,	Interv Med Appl Sci	9	2015	Jazan University	Observational Studies	Jizan	None	None	10 Ear & mastoid	Disorders with hearing impairment	Acquired hearing impairment
26857311	Electrically evoked compound action potential (ECAP) in cochlear implant children: Changes in auditory nerve response in first year of cochlear implant use	Telmesani LM, Said NM,	Int J Pediatr Otorhinolaryngol	1	2016	Imam Abdulrahman Bin Faisal University	Observational Studies	Eastern Province	None	None	24 Factors influencing health status or contact with health services	Reasons for contact with the health services	Presence of device, implants or grafts
27090029	Hearing loss among adults with sickle cell disease in an endemic region: a prospective case-control study	Al Jabr I,	Ann Saudi Med	3	2016	King Faisal University	Observational Studies	Eastern Province	None	None	10 Ear & mastoid	Disorders with hearing impairment	Acquired hearing impairment

PMID	Title	Authors	Journal/Book	Month	Year	First author institution	Type of Publication	Region	Funding	COI	ICD11 Chapter	ICD11 Block	ICD11 Category
27185713	Dexmedetomidine suppresses intractable hiccup during anesthesia for cochlear implantation	El-Tahan MR, Doyle DJ, Telmesani L, Al'Ghamdi A, Khidr AM, Abdeen MM,	J Clin Anesth	6	2016	Imam Abdulrahman Bin Faisal University	Observational Studies	Eastern Province	None	Dr El Tahan received free airway device samples from Ambu in April 2014 for use in another study	24 Factors influencing health status or contact with health services	Reasons for contact with the health services	Presence of device, implants or grafts
27260597	Deaf or hard of hearing children in Saudi Arabia: Status of early intervention services	Alyami H, Soer M, Swanepoel A, Pottas L,	Int J Pediatr Otorhinolaryngol	4	2016	University of Pretoria	Observational Studies	Riyadh	None	None	24 Factors influencing health status or contact with health services	Reasons for contact with the health services	Presence of device, implants or grafts
27392833	The development of the ICF vestibular environmental scale	Whitney SL, Alghadir A, Alghwiri A, Alshebber KM, Alshehri M, Furman JM, Mueller M, Grill E,	J Vestib Res	7	2016	King Saud University	Observational Studies	Riyadh	None	None	21 Symptoms, signs or clinical findings, not elsewhere classified	Symptoms, signs or clinical findings of the nervous system	Symptoms or signs involving the nervous system
27652360	Prevalence of inner ear anomalies among cochlear implant candidates	Aldhafeeri AM, Alsanosi AA,	Saudi Med J	10	2016	King Saud University	Observational Studies	Riyadh	None	None	24 Factors influencing health status or contact with health services	Reasons for contact with the health services	Presence of device, implants or grafts
27729145	Postoperative diffusion weighted MRI and preoperative CT scan	Alzahrani M, Alhazmi R, B√©lair M, Saliba I,	Int J Pediatr Otorhinolaryngol	10	2016	King Fahad Specialist Hospital	Observational Studies	Eastern Province	None	None	10 Ear & mastoid	Diseases of middle ear or mastoid	Cholesteatoma of middle ear

PMID	Title	Authors	Journal/Book	Month	Year	First author institution	Type of Publication	Region	Funding	COI	ICD11 Chapter	ICD11 Block	ICD11 Category
	fusion for residual cholesteatoma localization												
27766948	Utilization of amplicon-based targeted sequencing panel for the massively parallel sequencing of sporadic hearing impairment patients from Saudi Arabia	Dallol A, Daghistani K, Elaimi A, Al-Wazani WA, Bamanie A, Safiah M, Sagaty S, Taha L, Zahed R, Bajouh O, Chaudhary AG, Gari MA, Turki R, Al-Qahtani MH, Abuzenadah AM,	BMC Med Genet	10	2016	King Abdulaziz University	Observational Studies	Makkah	None	None	10 Ear & mastoid	Disorders with hearing impairment	Congenital hearing impairment
27918383	Etiology of Acute Otitis Media in Children Less Than 5 Years of Age: A Pooled Analysis of 10 Similarly Designed Observational Studies	Van Dyke MK, Pir√ßon JY, Cohen R, Madhi SA, Rosenbl√ºt A, Macias Parra M, Al-Mazrou K, Grevers G, Lopez P, Naranjo L, Pumarola F, Sonsuwan N, Hausdorff WP,	Pediatr Infect Dis J	3	2017	King Saud University	Observational Studies	Riyadh	None	None	10 Ear & mastoid	Diseases of middle ear or mastoid	Otitis media
28012532	Management of surgical difficulties during cochlear implant with inner ear anomalies	Aldhafeeri AM, Alsanosi AA,	Int J Pediatr Otorhinolaryngol	11	2017	King Saud University	Observational Studies	Riyadh	None	None	24 Factors influencing health status or contact with health services	Reasons for contact with the health services	Presence of device, implants or grafts

PMID	Title	Authors	Journal/Book	Month	Year	First author institution	Type of Publication	Region	Funding	COI	ICD11 Chapter	ICD11 Block	ICD11 Category
28188119	Incidence and economic burden of acute otitis media in children aged up to 5years in three Middle Eastern countries and Pakistan: A multinational, retrospective, observational study	Mustafa G, Al Aidaroos AY, Al Abaidani IS, Meszaros K, Gopala K, Ceyhan M, Al-Tannir M, DeAntonio R, Bawikar S, Schmidt JE,	J Epidemiol Glob Health	2	2017	Prince Sultan Military Medical City	Observational Studies	Riyadh	None	None	10 Ear & mastoid	Diseases of middle ear or mastoid	Otitis media
28302169	Two novel LHX3 mutations in patients with combined pituitary hormone deficiency including cervical rigidity and sensorineural hearing loss	Ramzan K, Bin-Abbas B, Al-Jomaa L, Allam R, Al-Owain M, Imtiaz F,	BMC Endocr Disord	3	2017	King Faisal Specialist Hospital and Research Centre	Observational Studies	Riyadh	None	None	10 Ear & mastoid	Disorders with hearing impairment	Congenital hearing impairment
28377547	The use of an illuminated retractor in cochlear implantation: a comparative retrospective study	Alrashidi E, Almuhawas FA, Hagr A, Garadat S,	Ann Saudi Med	3	2017	King Saud University	Observational Studies	Riyadh	King Saud University	None	24 Factors influencing health status or contact with health services	Reasons for contact with the health services	Presence of device, implants or grafts
28469144	A deep intronic CLRN1 (USH3A) founder mutation generates an aberrant exon and underlies severe Usher	Khan AO, Becirovic E, Betz C, Neuhaus C, Altm√ºller J, Maria Riedmayr L, Motameny S, N√ºrnberg G,	Sci Rep	5	2017	King Khaled Eye Specialist Hospital	Observational Studies	Riyadh	None	None	20 Developmental anomalies	Multiple developmental anomalies or syndromes	Syndromic genetic deafness

PMID	Title	Authors	Journal/Book	Month	Year	First author institution	Type of Publication	Region	Funding	COI	ICD11 Chapter	ICD11 Block	ICD11 Category
	syndrome on the Arabian Peninsula	N√ºrnberg P, Bolz HJ,											
28566977	Cochlear implants in children: A cross-sectional investigation on the influence of geographic location in Saudi Arabia	Al-Sayed AA, AlSanosi A,	J Family Community Med	8	2017	King Saud University	Observational Studies	Riyadh	King Saud University	None	24 Factors influencing health status or contact with health services	Reasons for contact with the health services	Presence of device, implants or grafts
28644112	Susac syndrome misdiagnosed as multiple sclerosis with exacerbation by interferon beta therapy	Algahtani H, Shirah B, Amin M, Altarazi E, Almarzouki H,	Neuroradiol J	6	2018	King Saud bin Abdulaziz University for Health Sciences	Observational Studies	Makkah	None	None	08 Diseases of the nervous system	Multiple sclerosis or other white matter disorders	Secondary white matter disorders
28820756	Telmesani Radiological Classification of the Location of the Vertical Segment of the Facial Nerve: Impact on Surgical Approach in Cochlear Implant Surgery	Telmesani LM, Alrammah MK,	Otol Neurotol	10	2017	Imam Abdulrahman Bin Faisal University	Observational Studies	Eastern Province	None	None	24 Factors influencing health status or contact with health services	Reasons for contact with the health services	Contact with health services for specific surgical interventions

PMID	Title	Authors	Journal/Book	Month	Year	First author institution	Type of Publication	Region	Funding	COI	ICD11 Chapter	ICD11 Block	ICD11 Category
28913967	The variation in quality and content of patient-focused health information on the Internet for otitis media	Joury A, Joraid A, Alqahtani F, Alghamdi A, Batwa A, Pines JM,	Child Care Health Dev	9	2018	King Fahad Medical City	Systematic review and Meta-Analysis	Riyadh	None	None	10 Ear & mastoid	Diseases of middle ear or mastoid	Otitis media
28964313	The risk ratio for development of hereditary sensorineural hearing loss in consanguineous marriage offspring	Sanyelbhaa H, Kabel A, Abo El-Naga HAE, Sanyelbhaa A, Salem H,	Int J Pediatr Otorhinolaryngol	7	2017	Menoufia University, Egypt	Observational Studies	Makkah	None	None	10 Ear & mastoid	Disorders with hearing impairment	Hereditary hearing loss
29048421	Recurrent variants in OTOF are significant contributors to prelingual nonsydromic hearing loss in Saudi patients	Almontashiri NAM, Alswaid A, Oza A, Al-Mazrou KA, Elrehim O, Tayoun AA, Rehm HL, Amr SS,	Genet Med	4	2018	Taibah University	Observational Studies	Madinah	Partners HealthCare Personalized Medicine	None	10 Ear & mastoid	Disorders with hearing impairment	Congenital hearing impairment
29172846	Use of beta blockers is associated with hearing loss	Al-Ghamdi BS, Rohra DK, Abuharb GAI, Alkofide HA, AlRuwaili NS, Shoukri MM, Cahusac PMB,	Int J Audiol	11	2018	King Faisal Specialist Hospital and Research Centre	Observational Studies	Riyadh	None	None	10 Ear & mastoid	Disorders with hearing impairment	Ototoxic hearing loss
29204917	The outcome of cochlear implantation among children with genetic syndromes	Alzhrani F, Alhussini R, Hudeib R, Alkaff T, Islam T, Alsanosi A,	Eur Arch Otorhinolaryngol	12	2018	King Saud University	Observational Studies	Riyadh	None	None	24 Factors influencing health status or contact with health services	Reasons for contact with the health services	Presence of device, implants or grafts

PMID	Title	Authors	Journal/Book	Month	Year	First author institution	Type of Publication	Region	Funding	COI	ICD11 Chapter	ICD11 Block	ICD11 Category
29288105	The Top 50 Most-Cited Articles on Acoustic Neuroma	Alfaifi A, AlMutairi O, Allhaidan M, Alsaleh S, Ajlan A,	World Neurosurg	3	2018	King Saud University	Systematic review and Meta-Analysis	Riyadh	None	None	02 Neoplasms	Neoplasms of central nervous system or related structures	Primary neoplasm of spinal cord, cranial nerves, paraspinal nerves or remaining parts of central nervous system
29605349	Utility of whole exome sequencing in the diagnosis of Usher syndrome: Report of novel compound heterozygous MYO7A mutations	Ramzan K, Al-Owain M, Huma R, Al-Hazzaa SAF, Al-Ageel S, Imtiaz F, Al-Sayed M,	Int J Pediatr Otorhinolaryngol	5	2018	King Faisal Specialist Hospital and Research Centre	Observational Studies	Riyadh	King Faisal Specialist Hospital and Research Centre | King Salman Centre for Disability Research | King Abdulaziz City for Science and Technology	None	20 Developmental anomalies	Multiple developmental anomalies or syndromes	Syndromic genetic deafness

PMID	Title	Authors	Journal/Book	Month	Year	First author institution	Type of Publication	Region	Funding	COI	ICD11 Chapter	ICD11 Block	ICD11 Category
29607067	Hearing loss and its association with occupational noise exposure among Saudi dentists: a cross-sectional study	Alabdulwahhab BM, Alduraiby RI, Ahmed MA, Albatli LI, Alhumain MS, Softah NA, Saleh S,	BDJ Open	11	2016	Riyadh Elm University	Observational Studies	Riyadh	Albir Charity Center	None	10 Ear & mastoid	Diseases of inner ear	Noise effects on inner ear
29620546	Comparison of cochlear duct length between the Saudi and non-Saudi populations	Alanazi A, Alzhrani F,	Ann Saudi Med	3	2018	King Saud University	Observational Studies	Riyadh	None	None	X Extension Codes	Anatomy and topography	Auditory system
29720579	Congenital Esophageal Atresia and Microtia in a Newborn Secondary to Mycophenolate Mofetil Exposure During Pregnancy: A Case Report and Review of the Literature	Alsebayel MM, Abaalkhail FA, Alsebayel FM, Alissa DA, Al-Jedai AH, Elsiesy H,	Am J Case Rep	5	2018	King Saud bin Abdulaziz University for Health Sciences	Observational Studies	Riyadh	None	None	20 Developmental anomalies	Structural developmental anomalies primarily affecting one body system	Structural developmental anomalies of the ear
30302575	Association of benign paroxysmal positional vertigo with vitamin D deficiency: a systematic review and meta-analysis	AlGarni MA, Mirza AA, Althobaiti AA, Al-Nemari HH, Bakhsh LS,	Eur Arch Otorhinolaryngol	11	2018	King Saud bin Abdulaziz University for Health Sciences	Systematic review and Meta-Analysis	Makkah	None	None	10 Ear & mastoid	Diseases of inner ear	Episodic vestibular syndrome

PMID	Title	Authors	Journal/Book	Month	Year	First author institution	Type of Publication	Region	Funding	COI	ICD11 Chapter	ICD11 Block	ICD11 Category
30368552	Auditory and language skills development after cochlear implantation in children with multiple disabilities	Mesallam TA, Yousef M, Almasaad A,	Eur Arch Otorhinolaryngol	1	2019	King Saud University	Observational Studies	Riyadh	King Saud University	None	24 Factors influencing health status or contact with health services	Reasons for contact with the health services	Presence of device, implants or grafts
30465632	Knowledge about cochlear implantation: A parental perspective	Aloqaili Y, Arafat AS, Almarzoug A, Alalula LS, Hakami A, Almalki M, Alhuwaimel L,	Cochlear Implants Int	3	2019	King Abdulaziz Medical City	Observational Studies	Riyadh	None	None	24 Factors influencing health status or contact with health services	Reasons for contact with the health services	Presence of device, implants or grafts
30514999	Actinomycosis of the Middle Ear and Mastoid	Sheikh MA, Alenzi S, Ahmadi AA, Mashragi A, Alsayid H, Abdullah A,	Med Arch	10	2018	King Fahad General Hospital	Observational Studies	Makkah	None	None	01 Certain infectious or parasitic diseases	Other bacterial diseases	Actinomycosis
30602348	Ambiguous skin ulcer on the ear pinna	Abdalla N,	Trop Doct	4	2019	King Khalid University	Observational Studies	Asir	None	None	21 Symptoms, signs or clinical findings, not elsewhere classified	Symptoms, signs or clinical findings involving the skin	Symptoms or signs involving the skin
30723867	Myringoplasty outcome. Five-year experience at a tertiary teaching hospital	Alharbi SM, Mohammed WY, Al Ardi T, Al-Qahtani AS,	Saudi Med J	2	2019	King Khalid University	Observational Studies	Asir	None	None	24 Factors influencing health status or contact with health services	Reasons for contact with the health services	Contact with health services for specific surgical interventions
30740202	A tale of 1 year: a case of bilateral conductive hearing loss due to bilateral ossicular	Aljehani M, Alshamani M,	J Surg Case Rep	1	2019	Ohud Hospital	Observational Studies	Madinah	None	None	10 Ear & mastoid	Diseases of middle ear or mastoid	Discontinuity or dislocation of ear ossicles

PMID	Title	Authors	Journal/Book	Month	Year	First author institution	Type of Publication	Region	Funding	COI	ICD11 Chapter	ICD11 Block	ICD11 Category
	chain disruption post head trauma												
30787843	Association of Flying Time with Hearing Loss in Military Pilots	Al-Omari AS, Al-Khalaf HM, Hussien NFM,	Saudi J Med Med Sci	8	2018	Armed Forces Aeromedical Centre	Observational Studies	Eastern Province	None	None	10 Ear & mastoid	Diseases of inner ear	Noise effects on inner ear
30933841	Age of identification of sensorineural hearing loss and Characteristics,ÄØof affected children: Findings from two cross-sectional studies in Saudi Arabia	Alkahtani R, Rowan D, Kattan N, Alwan NA,	Int J Pediatr Otorhinolaryngol	7	2019	Princess Nourah Bint Abdulrahman University	Observational Studies	Riyadh	Princess Nourah bint Abdulrahman University	None	10 Ear & mastoid	Disorders with hearing impairment	Hereditary hearing loss
30938557	Categories of Auditory Performance and Speech Intelligibility Ratings in Prelingually Deaf Children With Bilateral Implantation	Albalawi Y, Nidami M, Almohawas F, Hagr A, Garadat SN,	Am J Audiol	3	2019	Military Hospital rem	Observational Studies	Riyadh	None	None	24 Factors influencing health status or contact with health services	Reasons for contact with the health services	Presence of device, implants or grafts
30947614	Auditory and speech performance in cochlear implanted ANSD children	Alzhrani F, Yousef M, Almuhawas F, Almutawa H,	Acta Otolaryngol	3	2019	King Saud University	Observational Studies	Riyadh	None	None	24 Factors influencing health status or contact with health services	Reasons for contact with the health services	Presence of device, implants or grafts

PMID	Title	Authors	Journal/Book	Month	Year	First author institution	Type of Publication	Region	Funding	COI	ICD11 Chapter	ICD11 Block	ICD11 Category
30955015	Complications of post-cochlear implantation in 1027 adults and children	Halawani R, Aldhafeeri A, Alajlan S, Alzhrani F,	Ann Saudi Med	4	2019	King Saud University	Observational Studies	Riyadh	None	None	24 Factors influencing health status or contact with health services	Reasons for contact with the health services	Presence of device, implants or grafts
31051407	Endolymphatic sac tumor at the cerebellopontine angle: A case report and review of literature	Alkhotani A, Butt B, Khalid M, Binmahfoodh M, Al-Said Y,	Int J Surg Case Rep	4	2019	King Faisal Specialist Hospital and Research Centre	Observational Studies	Makkah	None	None	02 Neoplasms	Malignant neoplasms, stated or presumed to be primary, of specified sites, except of lymphoid, haematopoietic, central nervous system or related tissues	Malignant neoplasms, stated or presumed to be primary, of specified sites, except of lymphoid, haematopoietic, central nervous system or related tissues
31083081	The Effect of Cochlear Coverage on Auditory and Speech Performance in Cochlear Implant Patients	Doubi A, Almuhawas F, Alzhrani F, Doubi M, Aljutaili H, Hagr A,	Otol Neurotol	6	2019	King Saud University	Observational Studies	Riyadh	None	None	24 Factors influencing health status or contact with health services	Reasons for contact with the health services	Presence of device, implants or grafts
31095403	Audiology and Speech-Language Pathology Simulation Training on the 1-3-6 Early Hearing Detection and Intervention Timeline	Alanazi AA, Nicholson N,	Am J Audiol	6	2019	King Saud bin Abdulaziz University for Health Sciences	Observational Studies	Riyadh	None	None	24 Factors influencing health status or contact with health services	Reasons for contact with the health services	Contact with health services for nonsurgical interventions not involving devices

PMID	Title	Authors	Journal/Book	Month	Year	First author institution	Type of Publication	Region	Funding	COI	ICD11 Chapter	ICD11 Block	ICD11 Category
31126928	Limited auricular relapsing polychondritis in a child treated successfully with infliximab	Alqanatish JT, Alfarhan BA, Qubaiban SM,	BMJ Case Rep	6	2019	King Saud Bin Abdulaziz University for Health Sciences	Observational Studies	Riyadh	None	None	15 Diseases of the musculoskeletal system or connective tissue	Osteopathies or chondropathies	Chondropathies
31221953	Allergic Fungal Otomastoiditis in a Patient without Allergic Fungal Rhinosinusitis: A Case Report	Salamah MA, Al-Shamani M,	Am J Case Rep	6	2019	Ohud Hospital	Observational Studies	Madinah	None	None	10 Ear & mastoid	Diseases of middle ear or mastoid	Mastoiditis or related conditions
31295705	Acute profound sensorineural hearing loss as the initial manifestation of Hairy Cell Leukemia, Case Report and literature review	AlEnazi A, Alhedaithy R, Alfayez A, Alghonaim Y,	Int J Surg Case Rep	7	2019	Imam Abdulrahman Bin Faisal University	Observational Studies	Eastern Province	None	None	10 Ear & mastoid	Disorders with hearing impairment	Acquired hearing impairment
31423516	Objective and subjective results of the Bonebridge transcutaneous active direct-drive bone conduction hearing implant	Alzhrani F,	Saudi Med J	8	2019	King Saud University	Observational Studies	Riyadh	None	None	24 Factors influencing health status or contact with health services	Reasons for contact with the health services	Presence of device, implants or grafts
31478749	Public awareness of ear health and hearing loss in Jeddah, Saudi Arabia	Alshehri KA, Alqulayti WM, Yaghmoor BE, Alem H,	S Afr J Commun Disord	8	2019	King Abdulaziz University	Observational Studies	Makkah	None	None	10 Ear & mastoid	Disorders with hearing impairment	Hearing impairment (unspecified)

PMID	Title	Authors	Journal/Book	Month	Year	First author institution	Type of Publication	Region	Funding	COI	ICD11 Chapter	ICD11 Block	ICD11 Category
31538009	Parental Knowledge, Attitudes and Practices Towards Paediatric Ear Infections in Riyadh, Saudi Arabia: A quantitative study	Alharbi MM, Almasri MS, Aldayel AY, Alkhonezan SM,	Sultan Qaboos Univ Med J	5	2019	Imam Mohammad Ibn Saud Islamic University	Observational Studies	Riyadh	None	None	10 Ear & mastoid	Diseases of middle ear or mastoid	Otitis media
31580711	After a first prelingually deaf child, does the family learn a lesson?	Al-Shawi Y, Aldhwaihy LS, Bin Zuair AM, Alfallaj RM, Almuhawas F,	Ann Saudi Med	10	2019	King Saud University	Observational Studies	Riyadh	None	None	10 Ear & mastoid	Disorders with hearing impairment	Congenital hearing impairment
31700752	A Comparison of Surgical Auditory Nerve Response and Speech Outcomes in Patients with Post-meningitic Deafness and Without Cochlear Osteogenesis Who Underwent Cochlear Implantation	Alshaikh M, Alahmadi A, Albedry M, Alharbi A, Alenzi S, Almahyawi R, Mansouri N, Albaqeyah M, Alamri A, Alharbi AA, Aldajani A,	Cureus	9	2019	Royal Commission Hospital	Observational Studies	Eastern Province	None	None	24 Factors influencing health status or contact with health services	Reasons for contact with the health services	Presence of device, implants or grafts
31707409	Surgical considerations and speech outcomes in infants who undergo cochlear implantation. Experience of the King	Hajr EA, Alotaibi T, Alobida NW, Alsanosi AA,	Saudi Med J	11	2019	King Saud University	Observational Studies	Riyadh	None	None	24 Factors influencing health status or contact with health services	Reasons for contact with the health services	Presence of device, implants or grafts

PMID	Title	Authors	Journal/Book	Month	Year	First author institution	Type of Publication	Region	Funding	COI	ICD11 Chapter	ICD11 Block	ICD11 Category
	Abdullah Ear Specialist Center												
31750162	Cochlear Implantation in Children with Otitis Media	Alzhrani F, Alahmari MS, Al Jabr IK, Garadat SN, Hagr AA,	Indian J Otolaryngol Head Neck Surg	11	2019	King Saud University	Observational Studies	Riyadh	King Saud University	None	24 Factors influencing health status or contact with health services	Reasons for contact with the health services	Presence of device, implants or grafts
31804144	Rare otologic presentation of cat eye syndrome	Alamer L, Bassant S, Alhazmi R, Alzahrani M,	Ann Saudi Med	11	2019	King Fahad Specialist Hospital	Observational Studies	Eastern Province	None	None	20 Developmental anomalies	Chromosomal anomalies, excluding gene mutations	Duplications of the autosomes
31807378	Value of Newborn Hearing Screening on Early Intervention in the Saudi Population and Review of International Records	Alshawi YA, Al-Gazlan N, Alrawaf F, Almuhawas F,	Cureus	10	2019	Prince Sultan Military Medical City	Observational Studies	Riyadh	None	None	24 Factors influencing health status or contact with health services	Reasons for contact with the health services	Contact with health services for purposes of examination or investigation
31824167	Assessment Of Ambient-Noise Exposure Among Female Nurses In Surgical Cardiac Intensive Care Unit	Alduais SA, Salama KF,	J Multidiscip Healthc	12	2019	Saud Albabtain Cardiac Center	Observational Studies	Eastern Province	None	None	10 Ear & mastoid	Diseases of inner ear	Noise effects on inner ear

PMID	Title	Authors	Journal/Book	Month	Year	First author institution	Type of Publication	Region	Funding	COI	ICD11 Chapter	ICD11 Block	ICD11 Category
31854501	Identification of TMC1 as a relatively common cause for nonsyndromic hearing loss in the Saudi population	Ramzan K, Al-Owain M, Al-Numair NS, Afzal S, Al-Ageel S, Al-Amer S, Al-Baik L, Al-Otaibi GF, Hashem A, Al-Mashharawi E, Basit S, Al-Mazroea AH, Softah A, Sogaty S, Imtiaz F,	Am J Med Genet B Neuropsychiatr Genet	4	2020	King Faisal Specialist Hospital and Research Centre	Observational Studies	Riyadh	King Faisal Specialist Hospital and Research Centre | King Salman Centre for Disability Research | King Abdulaziz City for Science and Technology	None	10 Ear & mastoid	Disorders with hearing impairment	Congenital hearing impairment
32032300	Inter-rater Reliability and Validity of the Arabic Version of Categories of Auditory Performance-II (CAP-II) Among Children With Cochlear Implant	Al-Shawi Y, Mesallam TA, Alfallaj R, Aldrees T, Albakheet N, Alshawi M, Alotaibi T, Algahtani A,	Otol Neurotol	6	2020	King Saud University	Observational Studies	Riyadh	None	None	24 Factors influencing health status or contact with health services	Reasons for contact with the health services	Presence of device, implants or grafts

PMID	Title	Authors	Journal/Book	Month	Year	First author institution	Type of Publication	Region	Funding	COI	ICD11 Chapter	ICD11 Block	ICD11 Category
32050777	Diagnostic Value of 3D Segmentation in Understanding the Anatomy of Human Inner Ear Including Malformation Types	Alenzi S, Dhanasingh A, Alanazi H, Alsanosi A, Hagr A,	Ear Nose Throat J	9	2021	King Saud University	Observational Studies	Riyadh	None	Anandhan Dhanasingh is employed by MED-EL as the Head of CI Electrodes Research which is purely a scientific role and this study did not contain any marketing message.	X Extension Codes	Anatomy and topography	Auditory system
32066114	Middle ear odontoma: A case report and review of the literature	Aljbli G, Albakheet N, Alshawi A, Alshawi Y, Aljadhai Y, Shami I,	Int J Surg Case Rep	2	2020	King Saud bin Abdulaziz University for Health Sciences	Observational Studies	Riyadh	None	None	02 Neoplasms	Benign neoplasms, except of lymphoid, haematopoietic, central nervous system or related tissues	Benign mesenchymal neoplasms

PMID	Title	Authors	Journal/Book	Month	Year	First author institution	Type of Publication	Region	Funding	COI	ICD11 Chapter	ICD11 Block	ICD11 Category
32110744	A Novel TBX1 Variant Causing Hypoparathyroidism and Deafness	Alghamdi M, Al Khalifah R, Al Homyani DK, Alkhamis WH, Arold ST, Ekhzaimy A, El-Wetidy M, Kashour T, Halwani R,	J Endocr Soc	11	2019	King Saud University	Observational Studies	Riyadh	King Saud University	None	10 Ear & mastoid	Disorders with hearing impairment	Congenital hearing impairment
32114606	Relationship between proximity to a cochlear implant center and early presentation in children with congenital hearing loss	Al-Shawi YA, Alrawaf FK, Al-Gazlan NS, Al-Qahtani MM, Almuhawas FA,	Saudi Med J	3	2020	King Saud University	Observational Studies	Riyadh	None	None	24 Factors influencing health status or contact with health services	Reasons for contact with the health services	Presence of device, implants or grafts
32172694	Safety of tympanoplasty and ossiculoplasty performed by otorhinolaryngology trainees	Alzhrani F, Aldueb R, Alosaimi K, Islam T, Almuhawas F, Alsanosi A,	J Laryngol Otol	3	2020	King Saud University	Observational Studies	Riyadh	None	None	24 Factors influencing health status or contact with health services	Reasons for contact with the health services	Contact with health services for specific surgical interventions
32191386	Type 1 diabetes and hearing loss: Audiometric assessment and measurement of circulating levels of soluble receptor for advanced glycation end products	Al-Sofiani M, MacLeod S, Ghanim H, Stecker N, Hall J, Lippes H,	Diabetes Metab Res Rev	3	2020	King Saud University	Observational Studies	Riyadh	King Saud University	None	10 Ear & mastoid	Disorders with hearing impairment	hearing impairment (unspecified)

PMID	Title	Authors	Journal/Book	Month	Year	First author institution	Type of Publication	Region	Funding	COI	ICD11 Chapter	ICD11 Block	ICD11 Category
32213420	Impact of pediatric obesity on the prevalence and outcome of otitis media with effusion	Alaraifi AK, Alosfoor MA, Alsaab F,	Int J Pediatr Otorhinolaryngol	6	2020	King Saud Bin Abdulaziz University for Health Sciences	Observational Studies	Riyadh	None	None	10 Ear & mastoid	Diseases of middle ear or mastoid	Otitis media
32216576	White-matter integrity and hearing acuity decline in healthy subjects: Magnetic resonance tractography	Alhazmi FH,	Neuroradiol J	6	2020	Taibah University	Observational Studies	Madinah	None	None	10 Ear & mastoid	Disorders with hearing impairment	Acquired hearing impairment
32222081	Investigating Facial Nerve Stimulation After Cochlear Implantation in Adult and Pediatric Recipients	Alzhrani F, Halawani R, Basodan S, Hudeib R,	Laryngoscope	2	2021	King Saud University	Observational Studies	Riyadh	None	None	24 Factors influencing health status or contact with health services	Reasons for contact with the health services	Presence of device, implants or grafts
32280311	The Magnitude and Determinants of Tinnitus among Health Science Students at King Khalid University	Musleh A, Saad Alzahrani S, Al Shehri TK, Mohammed Abdullah Alqahtani S, Yahya Ali Yahya S, Oudah Saeed AlShahrani A,	ScientificWorldJournal	3	2020	King Khalid University	Observational Studies	Asir	None	None	21 Symptoms, signs or clinical findings, not elsewhere classified	Symptoms, signs or clinical findings of ear or mastoid process	Symptoms or signs involving the ear or mastoid process
32291431	Automated remote intraoperative cochlear implant device testing (CR220). Is it clinically efficient?	Hajr EA, Almuhawas FA,	Saudi Med J	4	2020	Imam Mohammad Ibn Saud Islamic University	Observational Studies	Riyadh	None	None	24 Factors influencing health status or contact with health services	Reasons for contact with the health services	Presence of device, implants or grafts

PMID	Title	Authors	Journal/Book	Month	Year	First author institution	Type of Publication	Region	Funding	COI	ICD11 Chapter	ICD11 Block	ICD11 Category
32312244	Parental knowledge and attitudes to childhood hearing loss and hearing services in Qassim, Saudi Arabia	Alsudays AM, Alharbi AA, Althunayyan FS, Alsudays AA, Alanazy SM, Al-Wutay O, Alenezi MM,	BMC Pediatr	4	2020	Prince Sultan Military Medical City	Observational Studies	Riyadh	None	None	10 Ear & mastoid	Disorders with hearing impairment	Hearing impairment (unspecified)
32330070	Shape of the Cochlear Basal Turn: An Indicator for an Optimal Electrode-to-Modiolus Proximity With Precurved Electrode Type	Khurayzi T, Dhanasingh A, Almuhawas F, Alsanosi A,	Ear Nose Throat J	1	2021	King Saud University	Observational Studies	Riyadh	None	The second author is employed by MED-EL GmbH, Austria as the Head of Translational Science Communication, a role which is purely scientific with no marketing activities.	X Extension Codes	Anatomy and topography	Auditory system

PMID	Title	Authors	Journal/Book	Month	Year	First author institution	Type of Publication	Region	Funding	COI	ICD11 Chapter	ICD11 Block	ICD11 Category
32373915	Influence of family environment on the outcomes of cochlear implantation in pediatric recipients	Alenzi SH, Halawani RT, Alshalan AM, Habis SA, Alsanosi AA,	Saudi Med J	5	2020	King Saud University	Observational Studies	Riyadh	None	None	24 Factors influencing health status or contact with health services	Reasons for contact with the health services	Presence of device, implants or grafts
32442819	Speech perception with simultaneous bilateral cochlear implants: Is there a unilateral predominance?	Hajr E, AlFayez M, Alzhrani F,	Int J Pediatr Otorhinolaryngol	8	2020	Imam Mohammad Ibn Saud Islamic University	Observational Studies	Riyadh	None	None	24 Factors influencing health status or contact with health services	Reasons for contact with the health services	Presence of device, implants or grafts
32493102	Direct measurement of cochlear parameters for automatic calculation of the cochlear duct length	Khurayzi T, Almuhawas F, Sanosi A,	Ann Saudi Med	5	2020	King Saud University	Observational Studies	Riyadh	None	None	X Extension Codes	Anatomy and topography	Auditory system
32543228	Otogenic Pneumocephalus After Tegmen Bone Reconstruction: A Case Report	Khurayzi T, Jan B, Bedaiwi R, Ajlan A, Elwatidy S, Alhabib S, Alsanosi A,	Ear Nose Throat J	1	2022	King Saud University	Observational Studies	Riyadh	None	None	08 Diseases of the nervous system	Dissociative neurological symptom disorder	Other specified diseases of the nervous system
32544908	Auditory Performance and Subjective Satisfaction with the ADHEAR System	Almuhawas F, Alzhrani F, Saleh S, Alsanosi A, Yousef M,	Audiol Neurootol	1	2021	King Saud University	Observational Studies	Riyadh	None	None	24 Factors influencing health status or contact with health services	Reasons for contact with the health services	Presence of device, implants or grafts

PMID	Title	Authors	Journal/Book	Month	Year	First author institution	Type of Publication	Region	Funding	COI	ICD11 Chapter	ICD11 Block	ICD11 Category
32784151	New Classification of Cochlear Hypoplasia Type Malformation: Relevance in Cochlear Implantation	Halawani RT, Dhanasingh A,	J Int Adv Otol	8	2020	Ohud Hospital	Observational Studies	Madinah	None	None	24 Factors influencing health status or contact with health services	Reasons for contact with the health services	Presence of device, implants or grafts
32979117	Performance of cochlear implant recipients fitted with triphasic pulse patterns	Alhabib SF, Abdelsamad Y, Yousef M, Alzhrani F,	Eur Arch Otorhinolaryngol	9	2021	King Saud University	Observational Studies	Riyadh	None	Yassin Abdelsamad is employed by MED-EL GmbH and he has scientific roles only without any marketing or sales activities	24 Factors influencing health status or contact with health services	Reasons for contact with the health services	Presence of device, implants or grafts
32986504	Middle Ear Implant in a Patient With Fibrous Dysplasia: An Alternative for Hearing Restoration	Al-Shawi Y, Alsughayer L, Alradhi A, Alzhrani F,	Ear Nose Throat J	6	2021	King Saud University	Observational Studies	Riyadh	None	Athair Alradhi is employed by MED-EL GmbH and had clinical support role only, witho	24 Factors influencing health status or contact with health services	Reasons for contact with the health services	Presence of device, implants or grafts

PMID	Title	Authors	Journal/Book	Month	Year	First author institution	Type of Publication	Region	Funding	COI	ICD11 Chapter	ICD11 Block	ICD11 Category
										ut any marketing activities or any role in manuscript writing.			
33026057	Validation and inter-rater reliability testing of the Arabic version of speech intelligibility rating among children with cochlear implant	Al-Shawi YA, Mesallam TA, Albakheet NM, Alshawi MA, Alfallaj RM, Aldrees TM, Algahtani AA, Alotaibi TO,	Saudi Med J	10	2020	Prince Sultan Military Medical City	Observational Studies	Riyadh	None	None	24 Factors influencing health status or contact with health services	Reasons for contact with the health services	Presence of device, implants or grafts
33113482	Cochlear electrode array tip fold-over in incomplete partition-I - A case report	Alsughayer L, Al-Shawi Y, Yousef M, Hagr A,	Int J Pediatr Otorhinolaryngol	12	2020	King Saud University	Observational Studies	Riyadh	None	None	24 Factors influencing health status or contact with health services	Reasons for contact with the health services	Presence of device, implants or grafts
33123632	Referral and Lost to System Rates of Two Newborn Hearing Screening Programs in Saudi Arabia	Alanazi AA,	Int J Neonatal Screen	6	2020	King Saud bin Abdulaziz University for Health Sciences	Observational Studies	Riyadh	None	None	24 Factors influencing health status or contact with health services	Reasons for contact with the health services	Contact with health services for purposes of examination or investigation

PMID	Title	Authors	Journal/Book	Month	Year	First author institution	Type of Publication	Region	Funding	COI	ICD11 Chapter	ICD11 Block	ICD11 Category
33173627	The Effectiveness of Hyperbaric Oxygen Therapy as Salvage Treatment for Sudden Sensorineural Hearing Loss: A Retrospective Study	Almutairi N, Alnofal E, Algouhi A, Bamajboor AS, Alzaher N,	Cureus	10	2020	King Faisal Specialist Hospital and Research Centre	Observational Studies	Riyadh	None	None	10 Ear & mastoid	Disorders with hearing impairment	Sudden idiopathic hearing loss
33194462	Descriptive Patterns of Deafness Among Pre-School Saudi Children Aged Two to Five Years Visiting Neurology Clinic From 2012 to 2017	Elmalik S, Alshawi S, AlQahtani AM, AlShammasi HS, Alruwaili A, Aldughaim A, Alkhalifa SA,	Cureus	10	2020	King Saud Medical City	Observational Studies	Riyadh	None	None	10 Ear & mastoid	Disorders with hearing impairment	Hearing impairment (unspecified)
33211016	Prevalence of and Factors Associated With Eustachian Tube Dysfunction Among the Public in Jeddah, Saudi Arabia: Cross-Sectional Survey-Based Study	Alshehri KA, Saggaf OM, Alshamrani HM, Alnefaie AM, Alghamdi KB,	Interact J Med Res	11	2020	King Abdulaziz University	Observational Studies	Makkah	None	None	10 Ear & mastoid	Diseases of middle ear or mastoid	Disorders of Eustachian tube

PMID	Title	Authors	Journal/Book	Month	Year	First author institution	Type of Publication	Region	Funding	COI	ICD11 Chapter	ICD11 Block	ICD11 Category
33242112	Evaluation of computed tomography parameters in patients with facial nerve stimulation post-cochlear implantation	Aljazeeri IA, Khurayzi T, Al-Amro M, Alzhrani F, Alsanosi A,	Eur Arch Otorhinolaryngol	10	2021	King Saud University	Observational Studies	Riyadh	None	None	24 Factors influencing health status or contact with health services	Reasons for contact with the health services	Presence of device, implants or grafts
33278246	Systematic Review of Postcochlear Implant Electrode Migration: What Is Known?	Alenzi S, Khurayzi T, Alshalan A, Almuhawas F, Alsanosi A,	Otol Neurotol	2	2021	King Saud University	Systematic review and Meta-Analysis	Riyadh	None	None	24 Factors influencing health status or contact with health services	Reasons for contact with the health services	Presence of device, implants or grafts
33283573	Is gelfoam sealing necessary in stapes surgery?	Bawazeer N, Zaouche S, Tringali S, Fieux M,	Acta Otolaryngol	3	2021	Umm Al-Qura University	Observational Studies	Makkah	None	None	24 Factors influencing health status or contact with health services	Reasons for contact with the health services	Contact with health services for specific surgical interventions
33302020	Effect of early activation of cochlear implant on electrode impedance in pediatric population	Alhabib SF, Abdelsamad Y, Yousef M, Alzhrani F, Hagr A,	Int J Pediatr Otorhinolaryngol	1	2021	King Saud University	Observational Studies	Riyadh	None	None	24 Factors influencing health status or contact with health services	Reasons for contact with the health services	Presence of device, implants or grafts
33307737	FORM24 electrode array and perioperative cerebrospinal fluid leakage in cochlear implant recipients with cochleovestibular	Halawani R, Alzhrani F, Almuhawas F, Hagr AA,	Ann Saudi Med	10	2020	Ohud Hospital	Observational Studies	Madinah	None	None	24 Factors influencing health status or contact with health services	Reasons for contact with the health services	Presence of device, implants or grafts

PMID	Title	Authors	Journal/Book	Month	Year	First author institution	Type of Publication	Region	Funding	COI	ICD11 Chapter	ICD11 Block	ICD11 Category
	malformations												
33307738	The association between consanguineous marriage and offspring with congenital hearing loss	Almazroua AM, Alsughayer L, Ababtain R, Al-Shawi Y, Hagr AA,	Ann Saudi Med	11	2020	King Saud University	Observational Studies	Riyadh	None	None	10 Ear & mastoid	Disorders with hearing impairment	Congenital hearing impairment
33316915	Identification of Novel CDH23 Variants Causing Moderate to Profound Progressive Nonsyndromic Hearing Loss	Ramzan K, Al-Numair NS, Al-Ageel S, Elbaik L, Sakati N, Al-Hazzaa SAF, Al-Owain M, Imtiaz F,	Genes (Basel)	12	2020	King Faisal Specialist Hospital and Research Centre	Observational Studies	Riyadh	King Faisal Specialist Hospital and Research Centre | King Salman Centre for Disability Research | King Abdulaziz City for Science and Technology	None	10 Ear & mastoid	Disorders with hearing impairment	Congenital hearing impairment

PMID	Title	Authors	Journal/Book	Month	Year	First author institution	Type of Publication	Region	Funding	COI	ICD11 Chapter	ICD11 Block	ICD11 Category
33335390	Multiple Dural Arteriovenous Fistulas Manifesting as Progressive Otalgia and Tinnitus and Treated Using a Single Session of Endovascular Embolization	Al-Abdulwahhab AH, Al-Suhibani S, Al-Sharydah AM, Al-Jubran SA, Al-Thuneyyan MA,	Clin Interv Aging	12	2020	Imam Abdulrahman Bin Faisal University	Observational Studies	Eastern Province	None	None	21 Symptoms, signs or clinical findings, not elsewhere classified	Symptoms, signs or clinical findings of ear or mastoid process	Symptoms or signs involving the ear or mastoid process
33520056	Treatment of auricular relapsing polychondritis in a Saudi child using only non-steroidal anti-inflammatory drugs: a case report	AlE'ed AA,	Pan Afr Med J	11	2020	Qassim University	Observational Studies	Qassim	None	None	15 Diseases of the musculoskeletal system or connective tissue	Osteopathies or chondropathies	Chondropathies
33563744	Clinical profile and management of revision cochlear implant surgeries	Aldhafeeri AM, Alzhrani F, Alajlan S, AlSanosi A, Hagr A,	Saudi Med J	2	2021	Hafr Albaten Central Hospital	Observational Studies	Eastern Province	None	None	24 Factors influencing health status or contact with health services	Reasons for contact with the health services	Presence of device, implants or grafts
33674548	Development of Arabic Version of Vestibular Disorders Activities of Daily Living Scale: Cross-Cultural Adaptation and Assessment of	Alshehri S, Tedla JS, Reddy RS, Silvian PS, Rengaramanujam K, Kakaraparthi VN, Ahmad I, Alahmari KA,	Med Sci Monit	3	2021	King Khalid University	Observational Studies	Asir	None	None	10 Ear & mastoid	Diseases of inner ear	Disorders of vestibular function

PMID	Title	Authors	Journal/Book	Month	Year	First author institution	Type of Publication	Region	Funding	COI	ICD11 Chapter	ICD11 Block	ICD11 Category
	Psychometric Properties												
33678717	Parents' Perspectives on Cochlear Implantation Results for Deaf Children or Children With Hearing Loss in Saudi Arabia	Alkhatani B,	Am Ann Deaf	12	2021	Prince Sattam bin Abdulaziz University	Observational Studies	Riyadh	None	None	24 Factors influencing health status or contact with health services	Reasons for contact with the health services	Presence of device, implants or grafts

PMID	Title	Authors	Journal/Book	Month	Year	First author institution	Type of Publication	Region	Funding	COI	ICD11 Chapter	ICD11 Block	ICD11 Category
33788034	Two-phase survey on the frequency of use and safety of MRI for hearing implant recipients	van de Heyning P, Mertens G, Topsakal V, de Brito R, Wimmer W, Caversaccio MD, Dazert S, Volkenstein S, Zernotti M, Parnes LS, Staecker H, Bruce IA, Rajan G, Atlas M, Friedland P, Skarzynski PH, Sugarova S, Kuzovkov V, Hagr A, Mlynski R, Schmutzhard J, Usami SI, Lassaletta L, Gavil√°n J, Godey B, Raine CH, Hagen R, Sprinzl GM, Brown K, Baumgartner WD, Karltorp E,	Eur Arch Otorhinolaryngol	3	2021	King Saud University	Observational Studies	Riyadh	None	All authors are members of the HEARRING Group, which is supported by MED-EL GmbH	24 Factors influencing health status or contact with health services	Reasons for contact with the health services	Presence of device, implants or grafts
33795738	A novel cochlear measurement that predicts inner-ear malformation	Khurayzi T, Almuhawas F, Alsanosi A, Abdelsamad Y, Doyle √ö, Dhanasingh A,	Sci Rep	4	2021	King Saud University	Systematic review and Meta-Analysis	Riyadh	King Saud University	None	X Extension Codes	Anatomy and topography	Auditory system

PMID	Title	Authors	Journal/Book	Month	Year	First author institution	Type of Publication	Region	Funding	COI	ICD11 Chapter	ICD11 Block	ICD11 Category
33866399	Cochlear implantation versus auditory brainstem implantation in children with auditory nerve deficiencies	Yousef M, Mesallam TA, Almasaad A, Alhabib S, Hagr A, Alzhrani F,	Eur Arch Otorhinolaryngol	3	2022	King Saud University	Observational Studies	Riyadh	None	None	24 Factors influencing health status or contact with health services	Reasons for contact with the health services	Presence of device, implants or grafts
33868854	Patterns and Correlations of Hearing Loss Among Adolescents, Adults, and Elderly in Saudi Arabia: A Retrospective Study	ALqarny M, Assiri AM, Alshehri A, Alharbi SM, Alshahrani EH, Alessa H, Alghubishi SA,	Cureus	3	2021	University of Bisha	Observational Studies	Asir	None	None	10 Ear & mastoid	Disorders with hearing impairment	Acquired hearing impairment
33954001	A Case Report of Complete Resolution of Auricular Mucormycosis in an 18-Month-Old Diabetic Child	Aljehani M, Alahmadi H, Alshamani M,	Case Rep Otolaryngol	2	2021	Ohud hospital	Observational Studies	Madinah	None	None	01 Certain infectious or parasitic diseases	Mycoses	Mucormycosis
34052873	Cochlear implantation in common cavity deformity: a systematic review	Al-Mahboob A, Alhabib SF, Abdelsamad Y, Alzhrani F,	Eur Arch Otorhinolaryngol	1	2022	King Saud University	Systematic review and Meta-Analysis	Riyadh	None	None	24 Factors influencing health status or contact with health services	Reasons for contact with the health services	Presence of device, implants or grafts
34077143	Empowering deaf and hard hearing females toward premarital counseling and genetic	Zaien SZ, El Sayed HA, Ibrahim HA, Elgzar WT, Aboraiah MIH, Abdel-Mordy MA,	Afr J Reprod Health	3	2021	Tabuk University	Observational Studies	Tabuk	None	None	10 Ear & mastoid	Disorders with hearing impairment	Hearing impairment (unspecified)

PMID	Title	Authors	Journal/Book	Month	Year	First author institution	Type of Publication	Region	Funding	COI	ICD11 Chapter	ICD11 Block	ICD11 Category
	screening: An educational intervention based on empowerment model												
34078733	The role of malleostapedotomy in intra-operative incus injury: A review of 2 cases	Alabdulqader AA, Hajr EA,	Saudi Med J	6	2021	Imam Mohammad Ibn Saud Islamic University	Observational Studies	Riyadh	None	None	24 Factors influencing health status or contact with health services	Reasons for contact with the health services	Contact with health services for specific surgical interventions
34085541	Hearing loss among patients with type 2 diabetes mellitus: a cross-sectional study	Al-Rubeaan K, AlMomani M, AlGethami AK, Darandari J, Alsalhi A, AlNaqeeb D, Almogbel E, Almasaari FH, Youssef AM,	Ann Saudi Med	5	2021	Sultan Bin Abdulaziz Humanitarian City	Observational Studies	Riyadh	Strategic Center for Diabetes Research KSU	None	10 Ear & mastoid	Disorders with hearing impairment	hearing impairment (unspecified)
34154450	Otolaryngology Manifestations of Primary Ciliary Dyskinesia: A Multicenter Study	Zawawi F, Shapiro AJ, Dell S, Wolter NE, Marchica CL, Knowles MR, Zariwala MA, Leigh MW, Smith M, Gajardo P, Daniel SJ,	Otolaryngol Head Neck Surg	3	2022	King Abdulaziz University	Observational Studies	Makkah	NCATS Rare Diseases Clinical Research Network (RDCRN)	None	20 Developmental anomalies	Structural developmental anomalies primarily affecting one body system	Structural developmental anomalies of the respiratory system

PMID	Title	Authors	Journal/Book	Month	Year	First author institution	Type of Publication	Region	Funding	COI	ICD11 Chapter	ICD11 Block	ICD11 Category
34194275	Hearing impairment in military personnel in Eastern Saudi Arabia	Alsaab FA, Alaraifi AK, Alhomaydan WA, Ahmed AZ, Elzubair AG,	J Family Community Med	5	2021	King Saud Bin Abdulaziz University for Health Sciences	Observational Studies	Eastern Province	King Abdullah International Medical Research Center	None	10 Ear & mastoid	Diseases of inner ear	Noise effects on inner ear
34341039	Is the outcome of fitting hearing aids to adults affected by whether an audiogram-based prescription formula is individually applied? A systematic review protocol	Almufarrij I, Dillon H, Munro KJ,	BMJ Open	7	2021	King Saud University	Systematic review and Meta-Analysis	Riyadh	NIHR Manchester Biomedical Research Centre	None	24 Factors influencing health status or contact with health services	Reasons for contact with the health services	Presence of device, implants or grafts
34344804	Magnet and receiver-stimulator displacement after cochlear implantation: Clinical characters and management approaches	Alahmadi A, Alenzi S, Alsheikh M, Alghamdi S, Morra ME, Badr KM,	Saudi Med J	8	2021	King Saud University	Systematic review and Meta-Analysis	Riyadh	None	None	24 Factors influencing health status or contact with health services	Reasons for contact with the health services	Presence of device, implants or grafts
34370603	Is COVID-19 associated with self-reported audio-vestibular symptoms?	AlJasser A, Alkeridy W, Munro KJ, Plack CJ,	Int J Audiol	8	2021	King Saud University	Observational Studies	Riyadh	King Saud University	None	21 Symptoms, signs or clinical findings, not elsewhere classified	Symptoms, signs or clinical findings of the nervous system	Symptoms or signs involving the nervous system

PMID	Title	Authors	Journal/Book	Month	Year	First author institution	Type of Publication	Region	Funding	COI	ICD11 Chapter	ICD11 Block	ICD11 Category
34388577	Distance Education for d/Deaf and Hard of Hearing Students during the COVID-19 Pandemic in Saudi Arabia: Challenges and Support	Alqraini FM, Alasim KN,	Res Dev Disabil	10	2021	Prince Sattam bin Abdulaziz University	Observational Studies	Riyadh	None	None	10 Ear & mastoid	Disorders with hearing impairment	Hearing impairment (unspecified)
34402951	Cochlear implantation among patients with otosclerosis: a systematic review of clinical characteristics and outcomes	Assiri M, Khurayzi T, Alshalan A, Alsanosi A,	Eur Arch Otorhinolaryngol	7	2022	King Saud University	Systematic review and Meta-Analysis	Riyadh	None	None	24 Factors influencing health status or contact with health services	Reasons for contact with the health services	Presence of device, implants or grafts
34425356	Correlation of quality of life with speech and hearing performance after pediatric cochlear implantation	Aldriweesh B, Alharbi M, Alzahrani M,	Int J Pediatr Otorhinolaryngol	11	2021	King Fahad Specialist Hospital	Observational Studies	Eastern Province	None	None	24 Factors influencing health status or contact with health services	Reasons for contact with the health services	Presence of device, implants or grafts
34427123	Clinical Approaches To External Auditory Canal Hemangiomas: A Systematic Review	Alshalan A, Khurayzi T, Assiri M, Alsanosi A,	Ear Nose Throat J	8	2021	King Saud University	Systematic review and Meta-Analysis	Riyadh	None	None	02 Neoplasms	Benign neoplasms, except of lymphoid, haematopoietic, central nervous system or related tissues	Benign mesenchymal neoplasms
34454193	Cochlear implant: More hearing better speech performance	Alhabib SF, Abdelsamad Y, Badghaish RS, Alzhrani F, Hagr A, Almuhawas F,	Int J Pediatr Otorhinolaryngol	11	2021	King Saud University	Observational Studies	Riyadh	None	None	24 Factors influencing health status or contact with health services	Reasons for contact with the health services	Presence of device, implants or grafts

PMID	Title	Authors	Journal/Book	Month	Year	First author institution	Type of Publication	Region	Funding	COI	ICD11 Chapter	ICD11 Block	ICD11 Category
34464417	Knowledge, attitude and management of hearing screening in children among family physicians in the Kingdom of Saudi Arabia	Alqudah O, Alqudah S, Al-Bashaireh AM, Alharbi N, Alqudah AM,	PLoS One	8	2021	King Fahad Medical City	Observational Studies	Riyadh	None	None	24 Factors influencing health status or contact with health services	Reasons for contact with the health services	Contact with health services for purposes of examination or investigation
34515055	The Difficulties Encountered by Pediatric Cochlear Implant Patients and Their Parents during the COVID-19 Pandemic	Telmesani LM, Said NM, Mahrous MM, Alrusayyis DF,	Audiol Neurootol	8	2022	Imam Abdulrahman Bin Faisal University	Observational Studies	Eastern Province	None	None	24 Factors influencing health status or contact with health services	Reasons for contact with the health services	Presence of device, implants or grafts
34729289	Salvaging Exposed Cochlear Implants	Arab K, Altamimi L, Al-Otaibi H, Kattan A, Gelidan AG,	Plast Reconstr Surg Glob Open	10	2021	King Saud University	Observational Studies	Riyadh	None	None	24 Factors influencing health status or contact with health services	Reasons for contact with the health services	Presence of device, implants or grafts
34736387	Changes in the Area Adjacent to the Internal Receiver-Stimulator of Cochlear Implant: A Retrospective Study	Aljazeeri I, Khurayzi T, Abdelsamad Y, Almuhawas F, Hagr A,	Curr Med Imaging	12	2021	King Saud University	Observational Studies	Riyadh	None	None	24 Factors influencing health status or contact with health services	Reasons for contact with the health services	Presence of device, implants or grafts
34853130	Guidelines for cochlear implantation in Saudi Arabia	Alzahrani MA, Aldajani NF, Alghamdi SA,	Saudi Med J	12	2021	King Fahad Specialist Hospital	Consensus	Eastern Province	NA	None	24 Factors influencing health status or contact with health services	Reasons for contact with the health services	Presence of device, implants or grafts

PMID	Title	Authors	Journal/Book	Month	Year	First author institution	Type of Publication	Region	Funding	COI	ICD11 Chapter	ICD11 Block	ICD11 Category
34926038	Association Between Acute Otitis Media and Inner Ear Disorders Among Adults in Aseer Region	Al-Shehri AM Sr, Al-Zomia AS, Alayash AF, M Al Hunaif A, A Mansour A, Alqahtani M, A Asiri O, A Alserhan S,	Cureus	11	2021	King Khalid University	Observational Studies	Asir	None	None	10 Ear & mastoid	Diseases of middle ear or mastoid	Otitis media
34928752	Prevalence, risk factors, and audiological characteristics of auditory neuropathy	Almishaal AA, Saleh S, Alferaih H, Alhelo O,	Int J Audiol	12	2022	University of Hail	Observational Studies	Hail	University of Ha,Äôil	None	10 Ear & mastoid	Disorders with hearing impairment	Auditory synaptopathy or neuropathy
34932226	Bilateral Cochlear Implantations in Temporal Bone Fracture: A Viable Treatment Option	Yousef MF, Theyab R, Garadat SN, Hagr A,	Laryngoscope	12	2021	King Saud University	Observational Studies	Riyadh	None	None	24 Factors influencing health status or contact with health services	Reasons for contact with the health services	Presence of device, implants or grafts
34967457	Anatomy-Based Frequency Allocation in Cochlear Implantation: The Importance of Cochlear Coverage	Aljazeeri I, Hamed N, Abdelsamad Y, Sharif T, Al-Momani M, Hagr A,	Laryngoscope	11	2022	King Saud University	Observational Studies	Riyadh	None	None	24 Factors influencing health status or contact with health services	Reasons for contact with the health services	Presence of device, implants or grafts
34976378	Awareness about the relation of noise induced hearing loss and use of headphones at Hail region	AlQahtani AS, Alshammari AN, Khalifah EM, Alnabri AA, Aldarwish HA, Alshammari KF, Alshammari	Ann Med Surg (Lond)	11	2021	University of Hail	Observational Studies	Hail	None	None	10 Ear & mastoid	Diseases of inner ear	Noise effects on inner ear

PMID	Title	Authors	Journal/Book	Month	Year	First author institution	Type of Publication	Region	Funding	COI	ICD11 Chapter	ICD11 Block	ICD11 Category
		HF, Almudayni AM,											
34976552	Cochlear Implant Complications in Children and Adults: Retrospective Analysis of 148 Cases	Garrada M, Alsulami MK, Almutairi SN, Alessa SM, Alselami AF, Alharbi NA, Alsulami RA, Talbi RY, Al-Nouri KI,	Cureus	12	2021	King Abdulaziz University	Observational Studies	Makkah	None	None	24 Factors influencing health status or contact with health services	Reasons for contact with the health services	Presence of device, implants or grafts
35013069	Complication Rates From Otoplasty at a Tertiary Facial Plastic Surgery Center: A Retrospective Analysis	AlAwadh I, Alassiry H, Bogari A, Alabduljabbar Z, AlDosari B,	J Craniofac Surg	9	2022	King Saud University	Observational Studies	Riyadh	None	None	24 Factors influencing health status or contact with health services	Reasons for contact with the health services	Contact with health services for specific surgical interventions
35061636	Vestibular and Balance Impairment Is Common in Children With Primary Ciliary Dyskinesia	Zawawi F, Papsin BC, Dell S, Cushing SL,	Otol Neurotol	3	2022	King Abdulaziz University	Observational Studies	Makkah	King Abdulaziz University	B.C.P.: Speaker's Bureau for Cochlear Corporation.	20 Developmental anomalies	Structural developmental anomalies primarily affecting one body system	Structural developmental anomalies of the respiratory system
35088612	Mucosal melanocytic lesion in the middle ear extending to the inner ear and nasopharynx	Alomar KS, Al-Shawi YA, Alzhrani F,	Ear Nose Throat J	9	2024	King Saud University	Observational Studies	Riyadh	None	None	21 Symptoms, signs or clinical findings, not elsewhere classified	Symptoms, signs or clinical findings involving the skin	Symptoms or signs involving the skin

PMID	Title	Authors	Journal/Book	Month	Year	First author institution	Type of Publication	Region	Funding	COI	ICD11 Chapter	ICD11 Block	ICD11 Category
35240596	Short- and Long-Term Self-Reported Audiovestibular Symptoms of SARS-CoV-2 Infection in Hospitalized and Nonhospitalized Patients	Almishaal AA, Alrushaidan AA,	Audiol Neurootol	3	2022	University of Hail	Observational Studies	Hail	None	None	21 Symptoms, signs or clinical findings, not elsewhere classified	Symptoms, signs or clinical findings of the nervous system	Symptoms or signs involving the nervous system
35290945	Management outcomes of otitis media with effusion in children with down syndrome: A systematic review	Sait S, Alamoudi S, Zawawi F,	Int J Pediatr Otorhinolaryngol	5	2022	King Abdulaziz University	Systematic review and Meta-Analysis	Makkah	None	None	10 Ear & mastoid	Diseases of middle ear or mastoid	Otitis media
35354340	Hemangioma of the External Auditory Canal and Temporal Bone: A Case Report and Comprehensive Literature Review	Marzouqi S, Roa H,	Ear Nose Throat J	12	2024	King Saud University	Observational Studies	Riyadh	None	None	02 Neoplasms	Benign neoplasms, except of lymphoid, haematopoietic, central nervous system or related tissues	Benign mesenchymal neoplasms
35360802	Knowledge and attitude of the general population regarding infant hearing loss in Saudi Arabia	Almutairi AN, Altuaysi AM, Alwhaid MS, Alhasson MA, Alharbi MA, Alsalam HA, Almazyadi HA, Almuqbil AA,	J Family Med Prim Care	2	2022	Qassim University	Observational Studies	Qassim	None	None	10 Ear & mastoid	Disorders with hearing impairment	Hearing impairment (unspecified)

PMID	Title	Authors	Journal/Book	Month	Year	First author institution	Type of Publication	Region	Funding	COI	ICD11 Chapter	ICD11 Block	ICD11 Category
35450002	Predictors of premarital screening and genetic counseling knowledge and attitude among deaf and hard hearing females in Tabuk, Saudi Arabia	Zaien SZ, El-Houfey AA, Alqahtani H, El Sayed HAE, Elgzar WT, Essa RM, Bayomy H, Ibrahim HA,	J Med Life	3	2022	Tabuk University	Observational Studies	Tabuk	University of Tabuk	None	10 Ear & mastoid	Disorders with hearing impairment	Hearing impairment (unspecified)
35499947	Transcutaneous Bone Conduction Implants in Patients With Single-Sided Deafness: Objective and Subjective Evaluation	AlFarraj A, AlIbrahim M, AlHajjaj H, Khater F, AlGhamdi A, Fayad J,	Ear Nose Throat J	5	2022	Johns Hopkins Aramco Healthcare	Observational Studies	Eastern Province	None	None	24 Factors influencing health status or contact with health services	Reasons for contact with the health services	Presence of device, implants or grafts
35537722	Osseointegrated device placement with minimally invasive surgery: Experience and audiological outcome	Aldhafeeri AM, Yousef M, Alzhrani F,	Saudi Med J	5	2022	Hafr Albaten Central Hospital	Observational Studies	Eastern Province	None	None	24 Factors influencing health status or contact with health services	Reasons for contact with the health services	Presence of device, implants or grafts
35594789	De novo sensorineural hearing loss sequelae of narrow, duplicated internal auditory canal: Case series and literature review	AlEnazi AS, Alshaiji A, Alenezi M, Al-Sharydah A, Alsuhibani S, Alhaidey A, Samarah A, AlQahtani M,	Int J Surg Case Rep	4	2022	Imam Abdulrahman Bin Faisal University	Observational Studies	Eastern Province	None	None	20 Developmental anomalies	Structural developmental anomalies primarily affecting one body system	Structural developmental anomalies of the ear

PMID	Title	Authors	Journal/Book	Month	Year	First author institution	Type of Publication	Region	Funding	COI	ICD11 Chapter	ICD11 Block	ICD11 Category
35612511	Speech performance and subjective satisfaction of middle ear implant in congenital aural atresia	Alzhrani F, Alhabib SF, Yousef M,	Acta Otorhinolaryngol Ital	4	2022	King Saud University	Observational Studies	Riyadh	None	None	20 Developmental anomalies	Structural developmental anomalies primarily affecting one body system	Structural developmental anomalies of the ear
35729690	Various approaches to the round window for cochlear implantation: a systematic review	Aljazeeri I, Alturaiki S, Abdelsamad Y, Alzhrani F, Hagr A,	J Laryngol Otol	10	2023	Aljaber Ophthalmology and Otolaryngology Specialized Hospital	Systematic review and Meta-Analysis	Eastern Province	None	None	24 Factors influencing health status or contact with health services	Reasons for contact with the health services	Presence of device, implants or grafts
35771280	Cochlear implantation in adults and pediatrics with enlarged vestibular aqueduct: a systematic review on the surgical findings and patients' performance	Alahmadi A, Abdelsamad Y, Salamah M, Alenzi S, Badr KM, Alghamdi S, Alsanosi A,	Eur Arch Otorhinolaryngol	12	2022	King Saud University	Systematic review and Meta-Analysis	Riyadh	None	None	24 Factors influencing health status or contact with health services	Reasons for contact with the health services	Presence of device, implants or grafts
35805610	Noise Mapping, Prevalence and Risk Factors of Noise-Induced Hearing Loss among Workers at Muscat International Airport	Al-Harthy NA, Abugad H, Zabeeri N, Alghamdi AA, Al Yousif GF, Darwish MA,	Int J Environ Res Public Health	6	2022	Imam Abdulrahman Bin Faisal University	Observational Studies	Eastern Province	Research Council (TRC) and the Ministry of Higher Education, Research and Innov	None	10 Ear & mastoid	Diseases of inner ear	Noise effects on inner ear

PMID	Title	Authors	Journal/Book	Month	Year	First author institution	Type of Publication	Region	Funding<br><br>ation of the Sultanate of Oman	COI	ICD11 Chapter	ICD11 Block	ICD11 Category
35830986	Predictors of tympanostomy tube extrusion time in otitis media with effusion	Alaraifi AK, Alkhaldi AS, Ababtain IS, Alsaab FA,	Saudi Med J	7	2022	King Saud Bin Abdulaziz University for Health Sciences	Observational Studies	Riyadh	None	None	24 Factors influencing health status or contact with health services	Reasons for contact with the health services	Contact with health services for specific surgical interventions
35859463	Hyperlipidemia and its relation with tinnitus: Cross-sectional approach	Musleh A, Alshehri S, Qobty A,	Niger J Clin Pract	7	2022	King Khalid University	Observational Studies	Asir	None	None	21 Symptoms, signs or clinical findings, not elsewhere classified	Symptoms, signs or clinical findings of ear or mastoid process	Symptoms or signs involving the ear or mastoid process
35861389	Mastoid Growth and the Configuration of Cochlear Implant Electrode Lead	Alhabib SF, Almuhawas F, Hagr A, Alzhrani F, Hamed N, Alenzi S, Abdelsamad Y, Dhanasingh A,	Ear Nose Throat J	7	2022	King Saud University	Observational Studies	Riyadh	None	None	24 Factors influencing health status or contact with health services	Reasons for contact with the health services	Presence of device, implants or grafts
35894536	Hearing Improvement After Pain Related to Cochlear Implant Explantation:	Aljazeeri I, Alrajhi M, Hagr A,	J Int Adv Otol	7	2022	King Saud University	Observational Studies	Riyadh	None	None	24 Factors influencing health status or contact with health services	Reasons for contact with the health services	Presence of device, implants or grafts

PMID	Title	Authors	Journal/Book	Month	Year	First author institution	Type of Publication	Region	Funding	COI	ICD11 Chapter	ICD11 Block	ICD11 Category
	A Case Report												
35910327	Epilepsy and Hearing Loss in a Patient with a Rare Heterozygous Variant in the CACNA1H Gene	Algahtani HA, Shirah BH, Samman A, Alhazmi A,	J Epilepsy Res	6	2022	King Abdulaziz Medical City	Observational Studies	Makkah	None	None	10 Ear & mastoid	Disorders with hearing impairment	Congenital hearing impairment
35952536	Pediatricians' knowledge and attitude toward hearing loss and newborn hearing screening programs	Malas M, Aboalfaraj A, Alamoudi H, Kurdi A, Alahmadi T, Zawawi F,	Int J Pediatr Otorhinolaryngol	10	2022	King Abdulaziz University	Observational Studies	Makkah	None	None	10 Ear & mastoid	Disorders with hearing impairment	Congenital hearing impairment
36060369	Prevalence of and Factors Associated With Eustachian Tube Dysfunction Among the Public in Taif, Saudi Arabia	Altalhi WA, Alsulaimani AI, Alhossaini ZA, Alharthi RM, Almalki ZA, Altalhi WA, Alswat SH, Alnefaie GO,	Cureus	7	2022	Taif University	Observational Studies	Makkah	None	None	10 Ear & mastoid	Diseases of middle ear or mastoid	Disorders of Eustachian tube
36088240	Positive and negative post stapedotomy effects on cervical VEMP recordings; a STROBE analysis	Riga M, Korres G, Tramontani O,	Eur Ann Otorhinolaryngol Head Neck Dis	5	2023	Dammam Medical Complex	Observational Studies	Eastern Province	None	None	24 Factors influencing health status or contact with health services	Reasons for contact with the health services	Contact with health services for specific surgical interventions

PMID	Title	Authors	Journal/Book	Month	Year	First author institution	Type of Publication	Region	Funding	COI	ICD11 Chapter	ICD11 Block	ICD11 Category
36105634	Intelligent Control Techniques for the Detection of Biomedical Ear Infections	Abdulaal MJ, Mehedi IM, Aljohani AJ, Milyani AH, Mahmoud M, Sahu MK, Abusorrah AM, Meem RJ,	Comput Intell Neurosci	9	2022	King Abdulaziz University	Observational Studies	Makkah	Ministry of Education	None	10 Ear & mastoid	Diseases of middle ear or mastoid	Otitis media
36122419	Brain MRI findings of prelingually deaf children and cochlear implant outcome: Preliminary results	Assiri M, Alshalan A, Alqahtani R, Abdelsamad Y, Alsanosi A,	Int J Pediatr Otorhinolaryngol	11	2022	King Saud University	Observational Studies	Riyadh	None	None	24 Factors influencing health status or contact with health services	Reasons for contact with the health services	Presence of device, implants or grafts
36123807	Musical Ear Syndrome in a Patient with Unilateral Hearing Loss: A Case Report	Aldhafeeri FM,	Am J Case Rep	9	2022	University of Hafr Al-Batin	Observational Studies	Eastern Province	None	None	10 Ear & mastoid	Disorders with hearing impairment	Acquired hearing impairment
36147053	Prosthesis extrusion post total ossicular replacement ossiculoplasty (TORP) following isotretinoin use: A case report and literature review of peri-operative isotretinoin safety	Alwabili M, Alotaibi N, Alamry S,	Ann Med Surg (Lond)	8	2022	Prince Sultan Military Medical City	Observational Studies	Riyadh	None	None	24 Factors influencing health status or contact with health services	Reasons for contact with the health services	Contact with health services for specific surgical interventions

PMID	Title	Authors	Journal/Book	Month	Year	First author institution	Type of Publication	Region	Funding	COI	ICD11 Chapter	ICD11 Block	ICD11 Category
36163238	Radiographers' awareness level of MRI-induced vertigo and their perspectives on the post-examination care provided to patients in Saudi Arabia	Alyami AS, Majrashi NA, Shubayr NA, Alatwah SM, Alyami J,	J Med Imaging Radiat Sci	12	2022	Jazan University	Observational Studies	Jizan	None	None	21 Symptoms, signs or clinical findings, not elsewhere classified	Symptoms, signs or clinical findings of the nervous system	Symptoms or signs involving the nervous system
36251258	Cochlear Implantation: The Variation in Cochlear Height	Alshalan A, Abdelsamad Y, Assiri M, Alsanosi A,	Ear Nose Throat J	10	2022	King Saud University	Observational Studies	Riyadh	None	None	24 Factors influencing health status or contact with health services	Reasons for contact with the health services	Presence of device, implants or grafts
36311152	The Effect of Various Patterns of Personal Listening Devices on Hearing Among University Students in Saudi Arabia	Alshamrani R, Altheeb F, Almasaoud H, Alghamdi A, Latif R, Rafique N, Sulaiman AA,	Acta Inform Med	9	2022	Imam Abdulrahman bin Faisal University	Observational Studies	Eastern Province	None	None	10 Ear & mastoid	Diseases of inner ear	Noise effects on inner ear
36482779	Pediatric Patient with Rhabdomyosarcoma Involving Temporal Bone: Case Report and Overview of Recent Cases	Alomar KS, Alhajress R, Alsheikh AS, Aljurayyad R, Alballaa A, Arafah M,	Am J Case Rep	12	2022	King Saud University	Observational Studies	Riyadh	None	None	02 Neoplasms	Malignant neoplasms, except primary neoplasms of lymphoid, haematopoietic, central nervous system or related tissues	Malignant mesenchymal neoplasms

PMID	Title	Authors	Journal/Book	Month	Year	First author institution	Type of Publication	Region	Funding	COI	ICD11 Chapter	ICD11 Block	ICD11 Category
36517062	Meningitis post-cochlear implant and role of vaccination	Alanazi GA, Alrashidi AS, Alqarni KS, Khozym SAA, Alenzi S,	Saudi Med J	12	2022	Tabuk University	Systematic review and Meta-Analysis	Tabuk	None	None	24 Factors influencing health status or contact with health services	Reasons for contact with the health services	Presence of device, implants or grafts
36533481	Assessing the Reading Instruction Knowledge of Teachers of the Deaf and Hard of Hearing: A Mixed-Methods Study	Alqraini FM,	Am Ann Deaf	9	2022	Prince Sattam bin Abdulaziz University	Observational Studies	Riyadh	None	None	10 Ear & mastoid	Disorders with hearing impairment	Hearing impairment (unspecified)
36544941	A novel three-step process for the identification of inner ear malformation types	Dhanasingh AE, Weiss NM, Erhard V, Altamimi F, Roland P, Hagr A, Van Rompaey V, Van de Heyning P,	Laryngoscope Investig Otolaryngol	NA	2022	MED,ÄêEL	Observational Studies	Riyadh	MED,ÄêEL GmbH	The authors Anandhan E. Dhanasingh and Varachaya Erhard are employed at MED,ÄêEL GmbH. This study did not carry any promotional content. All the	X Extension Codes	Anatomy and topography	Auditory system

PMID	Title	Authors	Journal/Book	Month	Year	First author institution	Type of Publication	Region	Funding	COI	ICD11 Chapter	ICD11 Block	ICD11 Category
										other authors have declared no conflict of interest.			
36546906	Comparative Study of Audiovestibular Symptoms between Early and Late Variants of COVID-19	Almishaal AA,	Audiol Res	NA	2022	University of Hail	Observational Studies	Hail	None	None	21 Symptoms, signs or clinical findings, not elsewhere classified	Symptoms, signs or clinical findings of the nervous system	Symptoms or signs involving the nervous system
36565078	Electrodermal and postural responses in dizzy adults: Diagnostic indicators of vestibular migraine	AlSharif DS, Tucker CA, Coffman DL, Keshner EA,	J Vestib Res	2	2023	King Faisal Medical Complex	Observational Studies	Makkah	None	None	10 Ear & mastoid	Diseases of inner ear	Episodic vestibular syndrome
36600848	Prevalence of Noise-Induced Tinnitus in Adults Aged 15 to 25 Years: A Cross-	Haji AK, Qashar AA, Alqahtani SH, Masarit RM, AlSindi TS, Ali-Eldin EM,	Cureus	11	2022	Umm Al-Qura University	Observational Studies	Makkah	None	None	21 Symptoms, signs or clinical findings, not elsewhere classified	Symptoms, signs or clinical findings of ear or mastoid process	Symptoms or signs involving the ear or mastoid process

PMID	Title	Authors	Journal/Book	Month	Year	First author institution	Type of Publication	Region	Funding	COI	ICD11 Chapter	ICD11 Block	ICD11 Category
	Sectional Study												
36604454	Method to estimate the basal turn length in inner ear malformation types	Alshalan A, Almuhawas F, Alhabib S, Hamed N, Abdelsamad Y, Dhanasingh A,	Sci Rep	1	2023	King Saud University	Observational Studies	Riyadh	None	None	X Extension Codes	Anatomy and topography	Auditory system
36640697	Cochlear implantation: Predicting the scala tympani volume of the pediatric recipients	Salamah M, Abdelsamad Y, Alahmadi A, Alsanosi A,	Int J Pediatr Otorhinolaryngol	2	2023	King Saud University	Observational Studies	Riyadh	None	None	24 Factors influencing health status or contact with health services	Reasons for contact with the health services	Presence of device, implants or grafts
36687225	The Incidence and Risk Factors of Cisplatin and Carboplatin Ototoxicity in Pediatric Oncology Patients at Tertiary Oncology Center	Attar M, Alqarni MS, Alsinnari YM, Bukhari ZM, Alshegifi H, Alzhrani A, Alshaikh K, Alsubaie H, Muqat M, Alhakami H, Algarni M,	Indian J Surg Oncol	12	2022	King Saud bin Abdulaziz University for Health Sciences	Observational Studies	Riyadh	None	None	10 Ear & mastoid	Disorders with hearing impairment	Ototoxic hearing loss
36733950	A Novel Otoplasty Technique Using Dermabrader on Prominent Ear Deformity Patients: A Retrospective Study and a	Gelidan AG, Mortada H, Alarki SMKZ, Arab K, Kattan AE,	Plast Reconstr Surg Glob Open	1	2023	King Saud University	Observational Studies	Riyadh	None	None	24 Factors influencing health status or contact with health services	Reasons for contact with the health services	Contact with health services for specific surgical interventions

PMID	Title<br><br>Description of Technique	Authors	Journal/Book	Month	Year	First author institution	Type of Publication	Region	Funding	COI	ICD11 Chapter	ICD11 Block	ICD11 Category
36742680	Predictors of Otitis Media with Effusion Recurrence Following Myringotomy	Alaraifi AK, Alkhaldi AS, Ababtain IS, Alsaab F,	Indian J Otolaryngol Head Neck Surg	NA	2022	King Abdulaziz Medical City	Observational Studies	Riyadh	None	None	10 Ear & mastoid	Diseases of middle ear or mastoid	Otitis media
36751176	Long-Term Outcomes of COVID-19 Otolaryngology Symptoms in Saudi Arabia	Alzahrani M, Alshathri AH, Alduraibi K, Alshathri AH, Alanazi TF, Alandijani H, Almajed JA, Wajdi KA,	Cureus	1	2023	Imam Mohammad Ibn Saud Islamic University	Observational Studies	Riyadh	NA	None	21 Symptoms, signs or clinical findings, not elsewhere classified	Symptoms, signs or clinical findings of the nervous system	Symptoms or signs involving the nervous system
36788895	Prevalence of Eustachian Tube Dysfunction and Its Associated Factors Among the General Public in Al-Madinah, Saudi Arabia	Alshamani MR, Alandijani HA, Alhussaini OM, Alharbi RA, Almeshaly SS, Alraddadi AF, Zakareya BF, Alrehaili RD, Alkenani FA, Jorob SM,	Cureus	1	2023	Ohud Hospital	Observational Studies	Madinah	None	None	10 Ear & mastoid	Diseases of middle ear or mastoid	Disorders of Eustachian tube
36820154	Long-term complications of the transmeatal approach (Open Transcanal) in cochlear implants: A	Almofada HS, Almutairi NK, Timms MS,	J Otol	1	2023	King Faisal Specialist Hospital and Research Centre	Observational Studies	Riyadh	None	None	24 Factors influencing health status or contact with health services	Reasons for contact with the health services	Presence of device, implants or grafts

PMID	Title	Authors	Journal/Book	Month	Year	First author institution	Type of Publication	Region	Funding	COI	ICD11 Chapter	ICD11 Block	ICD11 Category
	follow-up study												
36880843	The impact of the COVID-19 pandemic on rehabilitation services provided for cochlear implant recipients in Saudi Arabia	Al-Khalil RH, Al-Sowayan BS, Albdah B,	Ann Med	12	2023	King Abdullah Specialized Children's Hospital	Observational Studies	Riyadh	None	None	24 Factors influencing health status or contact with health services	Reasons for contact with the health services	Presence of device, implants or grafts
36939594	Qualitative Assessment of Quality and Readability of Patient-Directed Online Resources for Cochlear Implants in Children	Sanad SA, Mokhtar AM, Alharbi MO, Bukhari AF, Zawawi F,	Otolaryngol Head Neck Surg	7	2023	King Abdulaziz University	Observational Studies	Makkah	None	None	24 Factors influencing health status or contact with health services	Reasons for contact with the health services	Presence of device, implants or grafts
36983743	Cochlear Implantation in Pediatrics: The Effect of Cochlear Coverage	Alothman N, Almuhawas F, Badghaish R, Alotaibi AH, Alhabib SF, Alzhrani F, Hagr A,	J Pers Med	3	2023	Princess Nourah bint Abdulrahman University	Observational Studies	Riyadh	Ministry of Education	None	24 Factors influencing health status or contact with health services	Reasons for contact with the health services	Presence of device, implants or grafts
36996032	In search of language development for students who are hard of hearing: measuring the effectiveness of assistive	Alsalem MA, Alzahrani HA,	Assist Technol	3	2023	King Saud University	Experimental studies	Riyadh	King Saud University	None	10 Ear & mastoid	Disorders with hearing impairment	Hearing impairment (unspecified)

PMID	Title	Authors	Journal/Book	Month	Year	First author institution	Type of Publication	Region	Funding	COI	ICD11 Chapter	ICD11 Block	ICD11 Category
	technologies through teaching practices												
37012459	Perceived benefits of cochlear implants by parents: expectations, decision-making process, and barriers to care	Ibrahim AM, El-Gilany AH, Mohamed EWA, Farrag NS,	J Egypt Public Health Assoc	4	2023	Prince Sattam Bin Abdulaziz University	Observational Studies	Riyadh	Prince Sattam Bin Abdulaziz University	None	24 Factors influencing health status or contact with health services	Reasons for contact with the health services	Presence of device, implants or grafts
37012962	A Brief Review of Demographic and Clinical Correlates of Cholesteatoma Surgery in the Qassim Region	Alhazmi WA, Al Mansour MH, Aljasser RI, Alanazi AM, Alyami SD, Almutairi AB, Al Sulaiman IN,	Cureus	3	2023	Qassim University	Observational Studies	Qassim	None	None	10 Ear & mastoid	Diseases of middle ear or mastoid	Cholesteatoma of middle ear
37062553	Potential barriers to the daily use of hearing aids in children	Yousef MF, Dhayan ZI, Islam T, Alotabi FZ, Hajr EA,	Saudi Med J	4	2023	King Saud University	Observational Studies	Riyadh	Imam Mohammad Ibn Saud Islamic University	None	24 Factors influencing health status or contact with health services	Reasons for contact with the health services	Presence of device, implants or grafts
37077589	Role of Otolaryngologists in the Treatment of Patients With Riboflavin Transporter Deficiency: A Case Report	Alasqah MI, Aldriweesh B, Alshareef WA, Alhashem MH, Alammar A,	Cureus	3	2023	King Saud University	Observational Studies	Riyadh	None	None	10 Ear & mastoid	Disorders with hearing impairment	Congenital hearing impairment

PMID	Title	Authors	Journal/Book	Month	Year	First author institution	Type of Publication	Region	Funding	COI	ICD11 Chapter	ICD11 Block	ICD11 Category
37081199	Efficacy of gelfoam middle ear packing in type-1 tympanoplasty: systematic review and meta-analysis	Albazee E, Abu-Zaid A, Alshammari B, Salamah M, Alolaywi AN, Almobarak AA, Hagr A,	Eur Arch Otorhinolaryngol	4	2023	Alfaisal University	Systematic review and Meta-Analysis	Riyadh	None	None	24 Factors influencing health status or contact with health services	Reasons for contact with the health services	Contact with health services for specific surgical interventions
37090980	Dizziness in Saudi Arabia: An epidemiologic study	Alharbi AA, Alshammari ME, Albalwi AA, Ramadan MM, Alsharif DS, Hafiz AE,	Front Neurol	4	2023	Tabuk University	Observational Studies	Tabuk	None	None	21 Symptoms, signs or clinical findings, not elsewhere classified	Symptoms, signs or clinical findings of the nervous system	Symptoms or signs involving the nervous system
37097468	Early activation after cochlear implantation: a systematic review	Alshalan A, Abdelsamad Y, Yousef M, Alahmadi A, Almuhawas F, Hagr A,	Eur Arch Otorhinolaryngol	8	2023	Jouf University	Systematic review and Meta-Analysis	Al Jawf	MED-EL GmbH	None	24 Factors influencing health status or contact with health services	Reasons for contact with the health services	Presence of device, implants or grafts
37114087	Primary tuberculous otomastoiditis complicated with Bezold's, postauricular, and subdural abscesses: a case report and review of the literature	Aljehani N, Alzailaie A, Nassar J,	J Surg Case Rep	4	2023	King Saud bin Abdulaziz University for Health Sciences	Observational Studies	Riyadh	None	None	10 Ear & mastoid	Diseases of middle ear or mastoid	Mastoiditis or related conditions
37146078	The combined effect of gaze stability and balance exercises using telerehabilitation in individuals with	Aldawsary N, Almarwani M,	PLoS One	5	2023	King Saud University	Experimental studies	Riyadh	None	None	10 Ear & mastoid	Diseases of inner ear	Disorders of vestibular function

PMID	Title	Authors	Journal/Book	Month	Year	First author institution	Type of Publication	Region	Funding	COI	ICD11 Chapter	ICD11 Block	ICD11 Category
	vestibular disorders during the COVID-19 pandemic: A pilot study												
37153247	The Relationship Between Noise-Induced Hearing Loss Awareness and the Use of Personal Listening Devices in Makkah Region, Saudi Arabia	Alzahrani F, Alharthi SM, Alharthi SM, Kabli AF, Baabdullah A, Alzahrani AS, Baatiyyah E, Altowairqi AF, Alshareef S, Jan RM, Khafagy AA, Shatla MM,	Cureus	4	2023	Umm Al-Qura University	Observational Studies	Makkah	None	None	10 Ear & mastoid	Diseases of inner ear	Noise effects on inner ear
37236622	Giant Cell Tumor of the Temporal Bone and Skull Base	Amoodi H, Al-Domaidat D, Danish A, Alshaikh Hasan R,	J Craniofac Surg	10	2023	Dr. Soliman Fakeeh Hospital	Observational Studies	Makkah	None	None	02 Neoplasms	Malignant neoplasms, except primary neoplasms of lymphoid, haematopoietic, central nervous system or related tissues	Malignant neoplasms of ill-defined or unspecified primary sites
37384716	Pharmacists' communication skills with deaf and hard of hearing patients: A needs assessment	Al Aloola N, Alanazi M, Alotaibi N, Alwhaibi M,	PLoS One	6	2023	King Saud University	Observational Studies	Riyadh	None	None	10 Ear & mastoid	Disorders with hearing impairment	Hearing impairment (unspecified)
37398742	Measuring the Effect of Smoking on Hearing and Tinnitus Among the Adult Population in	Alateeq M, Alnizari O, Hafiz TA,	Cureus	5	2023	University of Hail	Observational Studies	Hail	None	None	21 Symptoms, signs or clinical findings, not elsewhere classified	Symptoms, signs or clinical findings of ear or mastoid process	Symptoms or signs involving the ear or mastoid process

PMID	Title	Authors	Journal/Book	Month	Year	First author institution	Type of Publication	Region	Funding	COI	ICD11 Chapter	ICD11 Block	ICD11 Category
	the Kingdom of Saudi Arabia												
37464460	Cochlear Implantation in Radiation-Induced Hearing Loss: A Systematic Review	Alahmadi A, Abdelsamad Y, Al-Zuraiqi B, Alghamdi S, Hagr A, Saleh E,	Otol Neurotol	9	2023	King Saud University	Systematic review and Meta-Analysis	Riyadh	None	NA	24 Factors influencing health status or contact with health services	Reasons for contact with the health services	Presence of device, implants or grafts
37540270	Comparison of temporalis muscle fascia and cartilage grafts for primary type 1 tympanoplasty: a meta-analysis of randomized controlled trials	Lajdam GB, Alahmadi RA, Alhakami M, Ghaddaf AA, Abdulhamid AS, Alahmadi A, Abdelsamad Y, Hagr A,	Eur Arch Otorhinolaryngol	8	2023	King Saud bin Abdulaziz University for Health Sciences	Systematic review and Meta-Analysis	Makkah	None	None	24 Factors influencing health status or contact with health services	Reasons for contact with the health services	Contact with health services for specific surgical interventions
37551795	A Literature Review on Cochlear Implant Activation: From Weeks to Hours	Alahmadi A, Abdelsamad Y, Alothman NI, Alshalan A, Almuhawas F, AlAmari NA, Alyousef MY, Alhabib SF, Hagr A,	Ear Nose Throat J	8	2023	King Saud University	Systematic review and Meta-Analysis	Riyadh	None	None	24 Factors influencing health status or contact with health services	Reasons for contact with the health services	Presence of device, implants or grafts
37606729	Effect of congenital inner ear malformations (IEMs) on electrically evoked compound	Said NM, Telmesani LS, Telmesani LM,	Eur Arch Otorhinolaryngol	12	2023	Imam Abdulrahman Bin Faisal University	Experimental studies	Eastern Province	None	None	24 Factors influencing health status or contact with health services	Reasons for contact with the health services	Presence of device, implants or grafts

PMID	Title	Authors	Journal/Book	Month	Year	First author institution	Type of Publication	Region	Funding	COI	ICD11 Chapter	ICD11 Block	ICD11 Category
	action potential (ECAP) responses in cochlear implant children												
37632735	Minimally Invasive OSIA Bone Conduction Hearing Implant (MOSIA) in Children: How I do it?	Alnoury MK, Daniel SJ,	Laryngoscope	4	2024	King Abdulaziz University	Observational Studies	Makkah	None	None	24 Factors influencing health status or contact with health services	Reasons for contact with the health services	Presence of device, implants or grafts
37654376	Parent Awareness and Perceived Barriers Regarding Hearing Impairment among School Age Children in Taif Region of Saudi Arabia	Fageeh YA, Alghoribi MH, Albishi MM, Alshanbari AA, Alqethami AA, Altowairqi TM, Alosaimi NK,	J Pharm Bioallied Sci	7	2023	Taif University	Observational Studies	Makkah	None	None	10 Ear & mastoid	Disorders with hearing impairment	Hearing impairment (unspecified)
37667917	Effects of Epley procedure on BPPV patients: a systematic review of randomized controlled trails	AlMohiza MA,	Eur Rev Med Pharmacol Sci	8	2023	King Saud University	Systematic review and Meta-Analysis	Riyadh	King Saud University	None	10 Ear & mastoid	Diseases of inner ear	Episodic vestibular syndrome
37775279	External auditory canal sebaceous carcinoma	Assiri SA, Altwairqi RG, Alotaibi R, Ibrahim M,	BMJ Case Rep	9	2023	King Faisal Medical Complex	Observational Studies	Makkah	None	None	02 Neoplasms	Malignant neoplasms, except primary neoplasms of lymphoid, haematopoietic, central nervous	Malignant neoplasms of skin

PMID	Title	Authors	Journal/Book	Month	Year	First author institution	Type of Publication	Region	Funding	COI	ICD11 Chapter	ICD11 Block	ICD11 Category
												system or related tissues	
37814445	Metastatic Malignant Glomus Jugulare Tumor: A Rare Case Report with Clinical Manifestations and Treatment Approach	Alomar KS, Alshammari NH, Alouda NS, AlGhamdi DA, Shami I,	Am J Case Rep	10	2023	King Saud University	Observational Studies	Riyadh	None	None	02 Neoplasms	Neoplasms of NA behaviour, except of lymphoid, haematopoietic, central nervous system or related tissues	Neoplasms of NA behaviour of endocrine glands
37883893	Exploring the role of copper and zinc in chronic otitis media: A novel spectrofluorometric method for precise determination and association study	Alaqel SL, Alzahrani MS, Alharbi A, Almalki AH, Algarni MA, Abdelazim MH, Abdelazim AH,	Spectrochim Acta A Mol Biomol Spectrosc	1	2024	Northern border university Arar	Observational Studies	Northern Borders	None	None	10 Ear & mastoid	Diseases of middle ear or mastoid	Otitis media
37885510	Quality of Life Among Adults With Hearing Loss Who Were Prescribed Hearing Aids in Aseer Province, Saudi Arabia: A Cross-Sectional Tertiary	Alrasheed AM, Junaid M, Ardi KT, Ebraheem FAM, Alaidaroos OZ,	Cureus	9	2023	Riyadh Third Health Cluster	Observational Studies	Asir	None	None	24 Factors influencing health status or contact with health services	Reasons for contact with the health services	Presence of device, implants or grafts

PMID	Title	Authors	Journal/Book	Month	Year	First author institution	Type of Publication	Region	Funding	COI	ICD11 Chapter	ICD11 Block	ICD11 Category
	Center-Based Study												
37888073	Simultaneous Bilateral Cochlear Implantation in Adults	Fatani N, Hamed N, Hagr A,	J Pers Med	10	2023	King Saud University	Observational Studies	Riyadh	None	None	24 Factors influencing health status or contact with health services	Reasons for contact with the health services	Presence of device, implants or grafts
37893135	A Current Landscape on Alport Syndrome Cases: Characterization, Therapy and Management Perspectives	Mahrous NN, Jamous YF, Almatrafi AM, Fallatah DI, Theyab A, Alanati BH, Alsagaby SA, Alenazi MK, Khan MI, Hawsawi YM,	Biomedicines	10	2023	University of Hafr Al-Batin	Systematic review and Meta-Analysis	Eastern Province	None	None	20 Developmental anomalies	Multiple developmental anomalies or syndromes	Syndromic genetic deafness
37899846	Risk factors and management strategies of inadvertent facial nerve stimulation in cochlear implant recipients: A systematic review	Alahmadi A, Abdelsamad Y, Yousef M, Alhabib SF, Alshalan A, Hamed N, Alzhrani F,	Laryngoscope Investig Otolaryngol	9	2023	King Saud University	Systematic review and Meta-Analysis	Riyadh	None	None	24 Factors influencing health status or contact with health services	Reasons for contact with the health services	Presence of device, implants or grafts
37908279	Alstr√∂m Syndrome: A Rare Cause of Severe Insulin Resistance	Radi S, Binmahfooz S, Nawar S, Malaikah H,	JCEM Case Rep	11	2022	King Saud bin Abdulaziz University for Health Sciences	Observational Studies	Makkah	None	None	20 Developmental anomalies	Multiple developmental anomalies or syndromes	Syndromic genetic deafness

PMID	Title	Authors	Journal/Book	Month	Year	First author institution	Type of Publication	Region	Funding	COI	ICD11 Chapter	ICD11 Block	ICD11 Category
38050886	Gradenigo Syndrome in a 6-Year-Old Boy with Acute Otitis Media: A Case Report	Al-Faifi JA,	Int Tinnitus J	12	2023	University of Bisha	Observational Studies	Asir	None	None	09 Diseases of the visual system	Strabismus or ocular motility disorders	Ocular motor nerve palsies
38057331	Enhancing cochlear duct length estimation by incorporating second-turn parameters	Alahmadi A, Abdelsamad Y, Dhanasingh A, Almuhawas F, Alsanosi A,	Sci Rep	12	2023	King Saud University	Observational Studies	Riyadh	none	None	X Extension Codes	Anatomy and topography	Auditory system
38070046	A novel coupling quality¬†index to estimate the coupling efficiency in Vibrant Soundbridge	Alahmadi A, Yousef M, Ibrahim A, Shahadah N, Hafez A, Abdelsamad Y, Alzhrani F,	Eur Arch Otorhinolaryngol	5	2024	King Saud University	Observational Studies	Riyadh	None	None	24 Factors influencing health status or contact with health services	Reasons for contact with the health services	Presence of device, implants or grafts
38088315	Cochlear Implant Single-Unit Audio Processors in Young Children	Saleh S, Almuhawas F, Alradhi A, Alhabib SF, Alzhrani F, Hagr A,	J Int Adv Otol	11	2023	King Saud University	Observational Studies	Riyadh	None	None	24 Factors influencing health status or contact with health services	Reasons for contact with the health services	Presence of device, implants or grafts
38107702	The impact of acute weight loss following bariatric surgery on Eustachian tube function	Alyahya K, Alarfaj A, AlBahr A, AlBahar S, Alsaleh M, Almuhaytib F, Alyahya A,	J Med Life	9	2023	King Faisal University	Observational Studies	Eastern Province	None	None	10 Ear & mastoid	Diseases of middle ear or mastoid	Disorders of Eustachian tube
38171933	Voice acoustic characteristics of children with late-onset cochlear implantation: Correlation to	Mahrous MM, Abdelgoad AA, Said NM, Telmesani LM, Alrusayyis DF,	Cochlear Implants Int	1	2024	Imam Abdulrahman Bin Faisal University	Observational Studies	Eastern Province	None	None	24 Factors influencing health status or contact with health services	Reasons for contact with the health services	Presence of device, implants or grafts

PMID	Title	Authors	Journal/Book	Month	Year	First author institution	Type of Publication	Region	Funding	COI	ICD11 Chapter	ICD11 Block	ICD11 Category
	auditory performance												
38206059	Advancing Cochlear Implant Programming: X-ray Guided Anatomy-Based Fitting	Alahmadi A, Abdelsamad Y, Thabet EM, Hafez A, Alghamdi F, Badr KM, Alghamdi S, Hagr A,	Otol Neurotol	2	2024	King Saud University	Observational Studies	Riyadh	None	None	24 Factors influencing health status or contact with health services	Reasons for contact with the health services	Presence of device, implants or grafts
38219511	Bilateral cochlear implants in a case of spondyloenchondrodysplasia with sensorineural hearing loss: Case report	Al Muslat AA, Alamry SS,	Int J Surg Case Rep	2	2024	Alfaisal University	Observational Studies	Riyadh	None	None	24 Factors influencing health status or contact with health services	Reasons for contact with the health services	Presence of device, implants or grafts
38246005	Role of cytokines and Th17/Tregs imbalance in the pathogenesis of otitis media with effusion. Modulation of Notch1/Hes1/mTORC1/S6k1 signalling pathway underlies the protective effect of astaxanthin	Mohamed NM, Abdelhamid AM, Aref M, Abdelhafeez M, Faris Alotabi H, Mohammed Abdelrahman DS, Elwany NE,	Int Immunopharmacol	2	2024	Princess Nourah bint Abdulrahman University	Experimental studies	Riyadh	Ministry of Education	None	10 Ear & mastoid	Diseases of middle ear or mastoid	Otitis media

PMID	Title	Authors	Journal/Book	Month	Year	First author institution	Type of Publication	Region	Funding	COI	ICD11 Chapter	ICD11 Block	ICD11 Category
38302541	Application of anatomy-based spacing of electrode contacts for achieving a uniform semitonal resolution: A novel concept in cochlear implant electrode design	Aljazeeri IA, Hagr A,	Sci Rep	2	2024	King Saud University	Observational Studies	Riyadh	None	None	24 Factors influencing health status or contact with health services	Reasons for contact with the health services	Presence of device, implants or grafts
38313764	Universal newborn hearing screening program in Saudi Arabia: Current insight	Alothman N, Elbeltagy R, Mulla R,	J Otol	1	2024	Princess Nourah bint Abdulrahman University	Observational Studies	Riyadh	None	None	24 Factors influencing health status or contact with health services	Reasons for contact with the health services	Contact with health services for purposes of examination or investigation
38363310	Complications of Cartilage Sparing Otoplasty: A Systematic Review and Meta-Analysis	Alanazi H,	J Craniofac Surg	2	2024	Majmaah University	Systematic review and Meta-Analysis	Riyadh	None	None	14 Diseases of the skin	Postprocedural disorders of the skin	Complications of cutaneous cosmetic procedures
38463405	Approach to Sudden Hearing Loss Among Primary Care Physicians in Riyadh, Saudi Arabia	Aldajani NF, Aloufi AM, Binhudayb NA, Yahya BJ, Alkarni AF,	Cureus	3	2024	King Fahad Medical City	Observational Studies	Riyadh	None	None	10 Ear & mastoid	Disorders with hearing impairment	Sudden idiopathic hearing loss
38507624	Noise-Induced Hearing Loss and Use of Hearing Protection Awareness among Medical	Alqarny M,	Int Tinnitus J	3	2024	University of Bisha	Observational Studies	Asir	None	None	10 Ear & mastoid	Diseases of inner ear	Noise effects on inner ear

PMID	Title	Authors	Journal/Book	Month	Year	First author institution	Type of Publication	Region	Funding	COI	ICD11 Chapter	ICD11 Block	ICD11 Category
	Students in Saudi Arabia: Mixed Qualitative and Quantitative Study												
38517009	Review and research gap identification in genetics causes of syndromic and nonsyndromic hearing loss in Saudi Arabia	Almalki F,	Ann Hum Genet	9	2024	Taibah University	Systematic review and meta-analysis	Madinah	None	None	10 Ear & mastoid	Disorders with hearing impairment	Congenital hearing impairment
38558666	Degree of Hearing Improvement and Reduction of Air-Bone Gap After Tympanoplasty in a Tertiary Hospital in Saudi Arabia	Al Hamoud M, Alzubaidi A, Al Shahrani K, Alotaibi G, Alkenani FA, Alahmari Y, Ardi TE, Al-Ahmari M, Asiri M,	Cureus	2	2024	Aseer Central Hospital	Observational Studies	Asir	None	None	24 Factors influencing health status or contact with health services	Reasons for contact with the health services	Contact with health services for specific surgical interventions
38588097	Parents' Perspectives on the Outcome of Cochlear Implantation for the Deaf Child and the Family in Saudi Arabia	Alasim K,	Am Ann Deaf	10	2023	Prince Sattam Bin Abdulaziz University	Observational Studies	Riyadh	Prince Sattam Bin Abdulaziz University	None	24 Factors influencing health status or contact with health services	Reasons for contact with the health services	Presence of device, implants or grafts
38647685	Efficacy of vibrant sound bridge in congenital aural atresia: an updated	Alshalan A, Alzhrani F,	Eur Arch Otorhinolaryngol	6	2024	Jouf University	Systematic review and Meta-Analysis	Al Jawf	Jouf University	None	20 Developmental anomalies	Structural developmental anomalies primarily affecting one body system	Structural developmental anomalies of the ear

PMID	Title	Authors	Journal/Book	Month	Year	First author institution	Type of Publication	Region	Funding	COI	ICD11 Chapter	ICD11 Block	ICD11 Category
	systematic review												
38649424	A novel method for evaluating mastoid defect regrowth after cochlear implantation	Hamed N, Alahmadi A, Abdelsamad Y, Alballaa A, Almuhawas F, Allami H, Almousa H, Hagr A,	Sci Rep	4	2024	King Saud University	Observational Studies	Riyadh	Ministry of Education	None	24 Factors influencing health status or contact with health services	Reasons for contact with the health services	Presence of device, implants or grafts
38673610	The Chronological Evolution of Cochlear Implant Contraindications: A Comprehensive Review	Hamed N, Alajmi N, Alkoblan FI, Alghtani YA, Abdelsamad Y, Alhussien A, Alhajress RI, Alhabib SF,	J Clin Med	4	2024	King Saud University	Systematic review and Meta-Analysis	Riyadh	None	Yassin Abdelsamad is employed by MED-EL GmbH with scientific roles only and without any marketing or sales activities.	24 Factors influencing health status or contact with health services	Reasons for contact with the health services	Presence of device, implants or grafts
38721579	Alstrom's Syndrome: An Experience of Tertiary Care Center	Gosadi G, Busehail M, Rahbeeni Z,	J Pediatr Genet	12	2021	Prince Mohammed Bin Nasser Hospital	Observational Studies	Jizan	None	None	20 Developmental anomalies	Multiple developmental anomalies or syndromes	Syndromic genetic deafness

PMID	Title	Authors	Journal/Book	Month	Year	First author institution	Type of Publication	Region	Funding	COI	ICD11 Chapter	ICD11 Block	ICD11 Category
38727252	Octyl-2-cyanoacrylate Tissue Adhesive Closure Versus Subcuticular Suture for Post-Auricular Incisions	Aljehani MJ, Salamah M, Halwani R, Alshamani M,	Laryngoscope	9	2024	Ohud Hospital	Experimental studies	Madinah	None	None	24 Factors influencing health status or contact with health services	Reasons for contact with the health services	Contact with health services for specific surgical interventions
38756130	Johanson-Blizzard syndrome caused by novel UBR1 mutation in four Saudi patients	Noli K, Aleysae N, Alzahrani I, Al-Ghamdi A, Alkazmi M, Almasoudi A,	JPGN Rep	3	2024	King Faisal Specialist Hospital and Research Centre	Observational Studies	Makkah	None	None	20 Developmental anomalies	Multiple developmental anomalies or syndromes	Syndromes with skin or mucosal anomalies as a major feature
38796944	Cochlear implantation in pediatrics: Impact of newborn hearing screening on intervention time	Alothman N, Abdelsamad Y, Almuhawas F, Allami H, Hagr A,	Int J Pediatr Otorhinolaryngol	6	2024	Princess Nourah bint Abdulrahman University	Observational Studies	Riyadh	Ministry of Education	None	24 Factors influencing health status or contact with health services	Reasons for contact with the health services	Presence of device, implants or grafts
38852235	Quality of Life of deaf adolescents in high school: A systematic literature review	Madhesh A,	Res Dev Disabil	8	2024	Shaqra University	Systematic review and Meta-Analysis	Riyadh	None	None	10 Ear & mastoid	Disorders with hearing impairment	Hearing impairment (unspecified)
38883102	Alstr√∂m Syndrome: A Challenging Case Study of a Female Saudi Patient With Type 2 Diabetes Mellitus and Complete Vision Loss	Alamri AS, Mahmoud HA, Abu Alnasr AA, Alahmadi AK, Qari YH,	Cureus	5	2024	Prince Mohammed Bin Abdulaziz National Guard Hospital	Observational Studies	Madinah	None	None	20 Developmental anomalies	Multiple developmental anomalies or syndromes	Syndromic genetic deafness

PMID	Title	Authors	Journal/Book	Month	Year	First author institution	Type of Publication	Region	Funding	COI	ICD11 Chapter	ICD11 Block	ICD11 Category
38893010	Cochlear Implantation: Long-Term Effect of Early Activation on Electrode Impedance	Alahmadi A, Abdelsamad Y, Yousef M, Almuhawas F, Hafez A, Alzhrani F, Hagr A,	J Clin Med	6	2024	King Saud University	Observational Studies	Riyadh	None	None	24 Factors influencing health status or contact with health services	Reasons for contact with the health services	Presence of device, implants or grafts
38907707	The Effect of Using Ofloxacin Ear Drops in Traumatic Tympanic Membrane Healing: A Systematic Review and Meta-Analysis	Kutbi AH, Malas M, Al-Talhi AA, Noori F, Amoodi HA,	Ear Nose Throat J	6	2024	King Abdulaziz Medical City	Systematic review and Meta-Analysis	Makkah	None	None	10 Ear & mastoid	Diseases of middle ear or mastoid	Perforation of tympanic membrane
38919244	Symptoms and Factors Associated With Eustachian Tube Dysfunction Among the Population of Qassim, Saudi Arabia	Almutairi A, Alharbi BA, Alharbi MT, Al-Sowinea AF, Alshammakhi AM, Munhish FA, Al-Harbi AM, Alabdulrahim JM,	Cureus	5	2024	Qassim University	Observational Studies	Qassim	None	None	10 Ear & mastoid	Diseases of middle ear or mastoid	Disorders of Eustachian tube
38941273	Ear care: Knowledge, behavior, and attitudes among healthcare practitioners in Najran City, Saudi Arabia	Almagribi AZM,	PLoS One	6	2024	Najran University	Observational Studies	Najran	None	None	V Supplementary section for functioning assessment	Generic functioning domains	Self-care
38968457	Evaluating the impact of COVID-19 on vertigo and hearing impairment:	Alshehri S, Alahmari KA,	Medicine (Baltimore)	7	2024	King Khalid University	Observational Studies	Asir	King Khalid University	None	10 Ear & mastoid	Disorders with hearing impairment	Acquired hearing impairment

PMID	Title	Authors	Journal/Book	Month	Year	First author institution	Type of Publication	Region	Funding	COI	ICD11 Chapter	ICD11 Block	ICD11 Category
	A post-recovery analysis												
38977475	Effect of balloon dilatation among adult population with eustachian tube dysfunction: a systematic review	Alghamdi AS, Aloufi BA, Almalki SM, Bosaeed KM,	Eur Arch Otorhinolaryngol	10	2024	Al-Hada Armed Foces Hospital	Systematic review and Meta-Analysis	Makkah	None	None	10 Ear & mastoid	Diseases of middle ear or mastoid	Disorders of Eustachian tube
39082905	A Systematic Review of the Clinical Effectiveness of Cochlear Implant Surgery in Pediatric and Adult Patients	Musleh A,	Niger J Clin Pract	7	2024	King Khalid University	Systematic review and Meta-Analysis	Asir	None	None	24 Factors influencing health status or contact with health services	Reasons for contact with the health services	Presence of device, implants or grafts
39113464	The First 100 Children Treated in a Newly Established Pediatric Vertigo Center	Alnoury MK, Salameh S, Ostrovska A, Gurberg J,	J Otolaryngol Head Neck Surg	1	2024	King Abdulaziz University	Observational Studies	Makkah	None	None	21 Symptoms, signs or clinical findings, not elsewhere classified	Symptoms, signs or clinical findings of the nervous system	Symptoms or signs involving the nervous system
39146844	The impact of hearing loss and cochlear implantation on the quality of life in children	Bukhari AF, Zawawi F,	Int J Pediatr Otorhinolaryngol	9	2024	King Abdulaziz University	Observational Studies	Makkah	None	None	24 Factors influencing health status or contact with health services	Reasons for contact with the health services	Presence of device, implants or grafts

PMID	Title	Authors	Journal/Book	Month	Year	First author institution	Type of Publication	Region	Funding	COI	ICD11 Chapter	ICD11 Block	ICD11 Category
39155857	Cochlear Implantation: Small Cochlear Diameter May Indicate Degree of Abnormality	Altamimi FN, Fatemah A, Al-Amro M, Al Montasher A, Al Otaibi S, Al Muhawas F,	J Int Adv Otol	3	2024	Alfaisal University	Observational Studies	Riyadh	None	None	24 Factors influencing health status or contact with health services	Reasons for contact with the health services	Presence of device, implants or grafts
39200858	Influence of Cochlear Anatomy on Intraoperative Electrically Evoked Compound Action Potentials	Fatani N, Abdelsamad Y, Alsanosi A,	J Clin Med	8	2024	King Saud University	Observational Studies	Riyadh	None	None	X Extension Codes	Anatomy and topography	Auditory system
39202074	Outcomes of Active Middle Ear Implants: Speech Perception and Quality of Life	Salamah M, Alradhi A, Alzhrani F, Yousef M,	J Pers Med	8	2024	King Saud University	Observational Studies	Riyadh	None	A.A works for MED-EL in the clinical support department without any marketing or sales activities.	24 Factors influencing health status or contact with health services	Reasons for contact with the health services	Presence of device, implants or grafts
39218468	Newborn hearing screening in Eastern Saudi Arabia: A tertiary hospital experience	Al-Shaikh Sulaiman AA,	Saudi Med J	8	2024	Imam Abdulrahman Bin Faisal University	Observational Studies	Eastern Province	None	None	24 Factors influencing health status or contact with health services	Reasons for contact with the health services	Contact with health services for purposes of examination or investigation

PMID	Title	Authors	Journal/Book	Month	Year	First author institution	Type of Publication	Region	Funding	COI	ICD11 Chapter	ICD11 Block	ICD11 Category
39229297	Simultaneous closure of a perilymphatic fistula and placement of cochlear implant in a case of complex inner ear malformation	Almuzaini H, M√ºller J, Wilhelm F, Polterauer D, Schuster M,	Clin Case Rep	9	2024	Taibah University	Observational Studies	Madinah	None	None	24 Factors influencing health status or contact with health services	Reasons for contact with the health services	Presence of device, implants or grafts
39254052	Efficacy of Adenoidectomy versus Tympanostomy on the Otitis Media with Effusion: A Systematic Review and Meta-Analysis	Alamri AA, Amoodi HA, Alsubaie SA, Alsehly AA, Alshuaibi RO, Alotaibi LA, Almaghrabi SJ, Mozahim SF, Mozahim NF, Tonkal A,	Afr J Paediatr Surg	10	2024	King Abdulaziz University	Systematic review and Meta-Analysis	Makkah	None	None	24 Factors influencing health status or contact with health services	Reasons for contact with the health services	Contact with health services for specific surgical interventions
39268302	Noise-Induced Hearing Loss Awareness and Prevention: A Cross-Sectional Study Among the Population of the Southern Region of Saudi Arabia	Alzahrani RA, Taishan WS, Ali M, Almaymoni AA, Althunayyan TS, Al Sulaiman IN, Hobani AB, Ibrahim ZA, Alharbi MO, Alzahrani T, Mony JM, Aljedaani Y,	Cureus	8	2024	Al Baha University	Observational Studies	Al Bahah	None	None	10 Ear & mastoid	Diseases of inner ear	Noise effects on inner ear
39311217	Tinnitus Prevalence, Associated Characteristics, and Treatment Patterns among Adults in Saudi Arabia	Alanazi AA,	Audiol Res	9	2024	King Saud bin Abdulaziz University for Health Sciences	Observational Studies	Riyadh	None	None	21 Symptoms, signs or clinical findings, not elsewhere classified	Symptoms, signs or clinical findings of ear or mastoid process	Symptoms or signs involving the ear or mastoid process

PMID	Title	Authors	Journal/Book	Month	Year	First author institution	Type of Publication	Region	Funding	COI	ICD11 Chapter	ICD11 Block	ICD11 Category
39328654	Aetiologies of Ear Infections Among Patients Who Visited King Fahad Hospital in Al-Baha, Saudi Arabia	Alfahemi H, Alghamdi M, Fadlalla MA, Halwani M, Elbadry RM, Alghamdi MK, Alghamdi FS, Alghamdi AM, Sallam TA,	Cureus	8	2024	Al Baha University	Observational Studies	Al Bahah	None	None	10 Ear & mastoid	Diseases of External Ear	Noninfectious inflammation of external ear
39379090	Hearing loss in patients with dementia in Saudi Arabia	Alqahtani L, Alotaibi L, Alkhunein J, Alduaiji R, Alqadiri R, Alibrahim F, binSalih S, Balubaid H,	Neurosciences (Riyadh)	10	2024	King Abdullah International Medical Research Center	Observational Studies	Riyadh	None	None	10 Ear & mastoid	Disorders with hearing impairment	Acquired hearing impairment
39405036	Adult Simultaneous Cochlear Implantation: Local Anesthesia	Fatani N, Alahmadi A, Almassalmeh Y, Abdelrahman AM, Alotaibi N, Yousef M, Alotaibi M, Aldokhayel F, Hagr A,	Ear Nose Throat J	10	2024	King Saud University	Observational Studies	Riyadh	None	None	24 Factors influencing health status or contact with health services	Reasons for contact with the health services	Presence of device, implants or grafts
39435307	A rare finding of pulmonary nodules in a middle ear neuroendocrine tumor: a case report and review of the literature	Alharbi N, Alotaibi G, Alabood S, AlSulaiman I, Bakry E,	J Surg Case Rep	10	2024	Buraidah Central Hospital	Observational Studies	Qassim	None	None	02 Neoplasms	Benign neoplasms, except of lymphoid, haematopoietic, central nervous system or related tissues	Benign neoplasm of middle ear, respiratory or intrathoracic organs
39441740	Retrospective Analysis of Cerebrospinal Gushers in Cochlear Implant Surgery: Incidence,	Hazazi M, Almashharawi E, Alamry S, Alkusayer MM, Altimyat A, Alsalamah Y,	Ear Nose Throat J	10	2024	Prince Sultan Military Medical City	Systematic review and Meta-Analysis	Riyadh	None	None	24 Factors influencing health status or contact with health services	Reasons for contact with the health services	Presence of device, implants or grafts

PMID	Title	Authors	Journal/Book	Month	Year	First author institution	Type of Publication	Region	Funding	COI	ICD11 Chapter	ICD11 Block	ICD11 Category
	Risk Factors, and Outcomes-A Systematic Review and Meta-analysis												
39442196	Evaluation of remote check in children with cochlear implants	Badghaish R, Alrushaydan D, Aljabr M, Al-Amro M, Yalcouy H,	Int J Pediatr Otorhinolaryngol	11	2024	King Saud University	Observational Studies	Riyadh	Cochlear Arabia Regional Headquarter	Cochlear Arabia Regional Headquarter	24 Factors influencing health status or contact with health services	Reasons for contact with the health services	Presence of device, implants or grafts
39485705	International survey of bimodal hearing and bilateral cochlear implant service provision for adults	Alfakhri M, Campbell N, Lineton B, Rowan D, Boyle P,	Cochlear Implants Int	11	2024	King Saud University	Observational Studies	Riyadh	King Saud University	None	24 Factors influencing health status or contact with health services	Reasons for contact with the health services	Presence of device, implants or grafts
39498437	Perrault syndrome: a forgotten presentation for infertile women	Alkhonezan M, Alkhonezan S, Al-Jaroudi D,	Clin Case Rep	11	2024	Imam Mohammad Ibn Saud Islamic University	Observational Studies	Riyadh	None	None	20 Developmental anomalies	Multiple developmental anomalies or syndromes	Syndromic genetic deafness
39516527	Estimation of outer-wall length in optimizing cochlear implantation in malformed inner ears	Alshalan A, Abdelsamad Y, Alahmadi A, Santoro F, Alhabib S, Almuhawas F, Alzhrani F, Alsanosi A, Dhanasingh A,	Sci Rep	11	2024	Jouf University	Observational Studies	Al Jawf	None	None	X Extension Codes	Anatomy and topography	Auditory system

PMID	Title	Authors	Journal/Book	Month	Year	First author institution	Type of Publication	Region	Funding	COI	ICD11 Chapter	ICD11 Block	ICD11 Category
39546436	X-ray guided anatomy-based fitting: The validity of OTOPLAN	Alahmadi A, Abdelsamad Y, Hafez A, Hagr A,	PLoS One	11	2024	King Saud University	Observational Studies	Riyadh	None	None	24 Factors influencing health status or contact with health services	Reasons for contact with the health services	Fitting, adjustment or management of devices
39575670	Cochlear Implantation: Predicting Round Window Niche Visibility Using One Measurement in High-Resolution Temporal Bone Computed Tomography	Telmesani L, Al-Ramah M, Abdelsamad Y, Telmesani L,	J Int Adv Otol	3	2024	Imam Abdulrahman bin Faisal University	Observational Studies	Eastern Province	None	None	24 Factors influencing health status or contact with health services	Reasons for contact with the health services	Presence of device, implants or grafts
39610809	Cochlear nerve visualization in Normal anatomy and inner ear malformations	Assiri M, Khurayzi T, Almuhawas F, Schlemmer K, Hagr A, Dhanasingh A,	Laryngoscope Investig Otolaryngol	11	2024	Abha Pediatric Hospital	Observational Studies	Asir	None	Anandhan Dhanasingh is employed by MED,ÄêEL as the Head of Electrodes Research & Inner Ear Malformations within R&D	X Extension Codes	Anatomy and topography	Auditory system

PMID	Title	Authors	Journal/Book	Month	Year	First author institution	Type of Publication	Region	Funding	COI	ICD11 Chapter	ICD11 Block	ICD11 Category
										department.			
39625087	School-based hearing screening of first-grade students in Saudi Arabia: A pilot study	Alothman NI, Alanazi AA,	S Afr J Commun Disord	11	2024	Princess Nourah bint Abdulrahman University	Observational Studies	Riyadh	None	None	24 Factors influencing health status or contact with health services	Reasons for contact with the health services	Contact with health services for purposes of examination or investigation
39644477	Diffuse face and ear hypertrichosis caused by 5% topical minoxidil in an adult woman with spontaneous resolution	Alkhayal FA, Alkinani AA,	Dermatol Online J	8	2024	Prince Sultan Military Medical City	Observational Studies	Riyadh	None	None	14 Diseases of the skin	Adverse cutaneous reactions to medication	Drug-induced hair abnormalities
39648290	Is the role of probe-tube, real-ear measurements changing for adults with mild-to-moderate hearing loss?	Almufarrij I, Sindi N, Pepler A, Munro KJ,	Int J Audiol	12	2024	King Saud University	Observational Studies	Riyadh	King Abdullah International Medical Research Center	None	10 Ear & mastoid	Disorders with hearing impairment	Hearing impairment (unspecified)

PMID	Title	Authors	Journal/Book	Month	Year	First author institution	Type of Publication	Region	Funding	COI	ICD11 Chapter	ICD11 Block	ICD11 Category
39658661	Hearing implants in pediatrics with cochlear nerve deficiency: an updated systematic review	Alahmadi A, Abdelsamad Y, AlAmari NA, Alyousef MY, Al-Momani M, Altamimi FN, Alhabib SF, Hagr A,	Eur Arch Otorhinolaryngol	12	2024	King Saud University	Systematic review and Meta-Analysis	Riyadh	None	None	24 Factors influencing health status or contact with health services	Reasons for contact with the health services	Presence of device, implants or grafts
39668226	The impact of cochlear implants on speech and language outcomes in pre-lingually deafened Arabic-speaking children: a systematic review	Alsari NAM,	Eur Arch Otorhinolaryngol	12	2024	King Saud University	Systematic review and Meta-Analysis	Riyadh	None	None	24 Factors influencing health status or contact with health services	Reasons for contact with the health services	Presence of device, implants or grafts
39692803	A spot review on relations between socioeconomic aspect and clinical recurrence of cholesteatoma	Aljehani MJ, Mukhtar RM, AlFallaj R, Alhusayni RA, Alraddadi RM, Alhussaini R,	Eur Arch Otorhinolaryngol	12	2024	Ohud Hospital	Observational Studies	Madinah	None	None	10 Ear & mastoid	Diseases of middle ear or mastoid	Cholesteatoma of middle ear
39710885	Robotic Versus Manual Electrode Insertion in Cochlear Implant Surgery: An Experimental Study	Alhabib SF, Alzhrani F, Alsanosi A, Al-Amro M, Alballaa A, Shami I, Hagr A, Alahmadi A, Sharif T, Stichling M, Matulic M, Assadi MZ, Abdelsamad Y, Almuhawas F,	Clin Exp Otorhinolaryngol	12	2024	King Saud University	Experimental studies	Riyadh	NA	NA	24 Factors influencing health status or contact with health services	Reasons for contact with the health services	Presence of device, implants or grafts

PMID	Title	Authors	Journal/Book	Month	Year	First author institution	Type of Publication	Region	Funding	COI	ICD11 Chapter	ICD11 Block	ICD11 Category
39765013	Consanguinity and hearing loss prevalence among Saudi Arabia population	Aljabri SF, Alrumaih SS, Mashhrawi YMA, Alhusayni MS, Bayamin RA, Kublan Alkublan RM, Binhazzaa A,	Int J Pediatr Otorhinolaryngol	12	2024	Imam Mohammad Ibn Saud Islamic University	Systematic review and Meta-Analysis	Riyadh	None	None	10 Ear & mastoid	Disorders with hearing impairment	Congenital hearing impairment
39776543	Exploration of preschool hearing screening at primary healthcare centers in Riyadh, Saudi Arabia: A cross-sectional study	Alanazi AA, Almutairi RA, Alsuhaibani KS, Alruwaily Y, Alqudiey HA, Alabbas MA, Alanazi SR,	J Otol	10	2024	King Saud bin Abdulaziz University for Health Sciences	Observational Studies	Riyadh	None	None	24 Factors influencing health status or contact with health services	Reasons for contact with the health services	Contact with health services for purposes of examination or investigation
28615800	Question mark ear deformity-revisited.	Mohammad M Al-Qattan; Noha M Al-Qattan	Eur J Plast Surg	11	2017	King Saud University	Observational Studies	Riyadh	King Saud University	None	20 Developmental anomalies	Structural developmental anomalies primarily affecting one body system	Structural developmental anomalies of the ear
38671619	Pediatric Acute Mastoiditis in Saudi Arabia: Demographic Insights, Clinical Profiles, and Prognostic Factors.	Sarah Alshehri; Khalid A Alahmari	Children (Basel)	3	2024	King Khalid University	Observational Studies	Asir	King Khalid University	None	10 Ear & mastoid	Diseases of middle ear or mastoid	Mastoiditis or related conditions
26309441	Inflammatory Myofibroblastic Tumor Arising in the External Ear: Unexpected Location. (Case Report).	Ahmed Al-Humidi; Abdullah Al-Khamiss	Int J Health Sci (Qassim)	4	2015	King Saud University	Observational Studies	Riyadh	NA	None	02 Neoplasms	Malignant neoplasms, except primary neoplasms of lymphoid, haematopoietic, central nervous system or related tissues	Malignant mesenchymal neoplasms

PMID	Title	Authors	Journal/Book	Month	Year	First author institution	Type of Publication	Region	Funding	COI	ICD11 Chapter	ICD11 Block	ICD11 Category
36505591	Relationship between tinnitus and headache in Riyadh, Saudi Arabia.	Yousef M Alluhaymid; Lojain J Alsiwat; Sundus Basodan; Murad Omar Almomani	J Family Med Prim Care	9	2022	King Saud University	Observational Studies	Riyadh	none	None	21 Symptoms, signs or clinical findings, not elsewhere classified	Symptoms, signs or clinical findings of ear or mastoid process	Symptoms or signs involving the ear or mastoid process
39018728	Referred otalgia in patients with thyroid cancer: Case series from Saudi Arabia.	Rajab Ahmed Alzahrani	Int J Surg Case Rep	8	2024	Al Baha University	Observational Studies	Al Bahah	none	None	10 Ear & mastoid	Disorders of ear, not elsewhere classified	Otalgia or effusion of ear
36836405	Cochlear Implantation: The Volumetric Measurement of Vestibular Aqueduct and Gusher Prediction.	Asma Alahmadi; Yassin Abdelsamad; Fida Almuhawas; Nezar Hamed; Marzouqi Salamah; Abdulrahman Alsanosi	J Pers Med	1	2023	King Saud University	Observational Studies	Riyadh	none	None	24 Factors influencing health status or contact with health services	Reasons for contact with the health services	Presence of device, implants or grafts
33681060	Awareness and attitudes of Saudi parents toward otitis media in children.	Mohammed Alsuhaibani; Muna Almijmaj; Abdulaziz Almushaigeh; Raghad Alhomidani; Yasser Aldakheel; Aqeel Alaqeel	J Family Med Prim Care	12	2020	Qassim University	Observational Studies	Qassim	none	None	10 Ear & mastoid	Diseases of middle ear or mastoid	Otitis media
33628279	Chronic Effects of Pediatric Ear Infections on Postural Stability.	Ohud A Sabir; Eric G Johnson; Ammar E Hafiz; Rhonda N Nelson; Mitali	Int J Pediatr	2	2021	King Abdulaziz University	Observational Studies	Makkah	King Abdulaziz University	None	10 Ear & mastoid	Diseases of middle ear or mastoid	Otitis media

PMID	Title	Authors	Journal/Book	Month	Year	First author institution	Type of Publication	Region	Funding	COI	ICD11 Chapter	ICD11 Block	ICD11 Category
		Hudlikar; Isha Sheth; Noha S Daher											
36993026	Practice of ear candling during lockdown due to COVID-19 in the Kingdom of Saudi Arabia.	Samar M Alanazi; Norah A Albdaya; Lama S Alhosaini; Fahad Z Alotaibi; Alhanouf A AlQabbani; Eman A Hajr	J Family Med Prim Care	11	2022	Imam Mohammad Ibn Saud Islamic University	Observational Studies	Riyadh	none	None	X Extension Codes	Dimensions of external causes	Objects, living things or substances involved in causing injury
20507343	C2orf37 mutational spectrum in Woodhouse-Sakati syndrome patients.	A M Alazami; S A Schneider; D Bonneau; L Pasquier; M Carecchio; M Kojovic; K Steindl; M de Kerdanet; M M Nezarati; K P Bhatia; B Degos; E Goh; F S Alkuraya	Clin Genet	12	2010	King Faisal Specialist Hospital and Research Centre	Observational Studies	Riyadh	King Faisal Specialist Hospital and Research Center	None	05 Endocrine, nutritional or metabolic diseases	Endocrine diseases	Disorders of the pituitary hormone system
39282550	Microbial spectrum, management challenges, and outcome in patients with otogenic skull base osteomyelitis.	Salma S AlSharhan; Marwan J Alwazzeh; Mona K ALRammah; Wasan F ALMarzouq; Aishah A AlGhuneem; Afnan J Alshrefy; Nada A Albahrani;	Infez Med	9	2024	Imam Abdulrahman Bin Faisal University	Observational Studies	Eastern Province	None	None	15 Diseases of the musculoskeletal system or connective tissue	Osteopathies or chondropathies	Osteomyelitis or osteitis

PMID	Title	Authors	Journal/Book	Month	Year	First author institution	Type of Publication	Region	Funding	COI	ICD11 Chapter	ICD11 Block	ICD11 Category
		Lena S Telmesani; Amal A AlGhamdi; Laila M Telmesani											
36570452	VOR gain of lateral semicircular canal using video head impulse test in acute unilateral vestibular hypofunction: A systematic review.	Mohamad Alfarghal; Mohammed Abdullah Algarni; Sujeet Kumar Sinha; Aishwarya Nagarajan	Front Neurol	12	2022	King Abdulaziz Medical City	Systematic review and Meta-Analysis	Makkah	NA	None	10 Ear & mastoid	Diseases of inner ear	Disorders of vestibular function
26380140	Aural Myiasis, a Rare Cause of Earache.	Ibrahim Al Jabr	Case Rep Otolaryngol	8	2015	King Faisal University	Observational Studies	Eastern Province	NA	None	01 Certain infectious or parasitic diseases	Parasitic diseases	Infestations by ectoparasites
27865143	The postauricular fasciocutaneous flap with an adipofascial extension: A case report.	Mohammed M Al-Qattan; Nawarah Al-Arfaj	Int J Surg Case Rep	11	2016	King Saud University	Observational Studies	Riyadh	King Saud University	None	24 Factors influencing health status or contact with health services	Reasons for contact with the health services	Contact with health services for specific surgical interventions
28875046	Ganglioneuroma of the External Auditory Canal and Middle Ear.	Hesham Saleh Almofada; Michael Steven Timms; M Anas Dababo	Case Rep Otolaryngol	8	2017	King Faisal Specialist Hospital and Research Centre	Observational Studies	Riyadh	None	None	02 Neoplasms	Neoplasms of central nervous system or related structures	Primary neoplasms of brain

PMID	Title	Authors	Journal/Book	Month	Year	First author institution	Type of Publication	Region	Funding	COI	ICD11 Chapter	ICD11 Block	ICD11 Category
21514926	Normal and pathological findings for the facial nerve on magnetic resonance imaging.	K Al-Noury; A Lotfy	Clin Radiol	8	2011	King Abdulaziz University	Observational Studies	Makkah	King Abdulaziz University	None	X Extension Codes	Anatomy and topography	Auditory system
38731136	Factors Influencing Treatment Success in Cholesteatoma Management: A Cross-Sectional Study.	Sarah Alshehri; Mohammed Abdullah M Al Shalwan; Abdulkhaliq Abdullah A Oraydan; Abdulrahman Saeed H Almuaddi; Ahmed Jubran A Alghanim	J Clin Med	4	2024	King Khalid University	Observational Studies	Asir	King Khalid University	None	10 Ear & mastoid	Diseases of middle ear or mastoid	Cholesteatoma of middle ear
33173680	Staged Ear Transfer and Reconstruction in a Severe Case of Neurofibromatosis Type 1.	Badr M I Abdulrauf	Plast Reconstr Surg Glob Open	10	2020	King Faisal Specialist Hospital and Research Centre	Observational Studies	Makkah	None	None	20 Developmental anomalies	Multiple developmental anomalies or syndromes	Phakomatoses or hamartoneoplastic syndromes
31681691	Light-assisted removal of ear canal live insect-A noninvasive approach for first level responders.	Amal Jaber Alfaifi; Liaqat Ali Khan; Hadi Mohammed Mokarbesh	J Family Med Prim Care	9	2019	Jazan Heatlh Affairs	Observational Studies	Jizan	None	None	22 Injury, poisoning or certain other consequences of external causes	Effects of foreign body entering through natural orifice	Foreign body in ear
38188895	Nephrotic syndrome: Pretibial epidermolysis bullosa in a patient with CD151 tetraspanin defect: A case report.	Khamisa Almokali; Hissah Alshalawi; Marwh G Aldriwesh; Raniah S Alotibi	Int J Health Sci (Qassim)	NA	2024	King Abdullah Specialized Children's Hospital	Observational Studies	Riyadh	None	None	14 Diseases of the skin	Genetic or developmental disorders affecting the skin	Genetically-determined epidermolysis bullosa

PMID	Title	Authors	Journal/Book	Month	Year	First author institution	Type of Publication	Region	Funding	COI	ICD11 Chapter	ICD11 Block	ICD11 Category
34660426	The need for a Smart Phone Application to Facilitate Communication Between Deaf-Mute and Hearing-Impaired Patients and Dentists.	Omar H Alkadhi; Baraa I Abdulrahman; Shiama A Alhawas; Leen A Almanie; Haifa E Alsalmi; Asayil A Aljumah	J Family Med Prim Care	8	2021	Riyadh Elm University	Observational Studies	Riyadh	None	None	10 Ear & mastoid	Disorders with hearing impairment	Hearing impairment (unspecified)
38727149	Optimized Management of Patulous Eustachian Tube: Shim Size Modifications to Minimize Middle Ear Effusion and Enhancing Treatment Outcomes.	Saleh AlAmry; Homood M Almutairi; Thamer M AlBilasi; Firas K Almarri	Ear Nose Throat J	5	2024	Prince Sultan Military Medical City	Observational Studies	Riyadh	None	None	10 Ear & mastoid	Disorders of ear, not elsewhere classified	Otalgia or effusion of ear
11924114	Biotinidase deficiency: a treatable genetic disorder in the Saudi population.	S Joshi; M A al-Essa; A Archibald; P T Ozand	East Mediterr Health J	11	1999	King Faisal Specialist Hospital and Research Centre	Observational Studies	Riyadh	NA	None	05 Endocrine, nutritional or metabolic diseases	Metabolic disorders	Inborn errors of metabolism

PMID	Title	Authors	Journal/Book	Month	Year	First author institution	Type of Publication	Region	Funding	COI	ICD11 Chapter	ICD11 Block	ICD11 Category
30697101	The Impact of using the term "Diabetic Ear" for the patients with Skull Base Osteomyelitis.	Abdulaziz S AlEnazi; Salma S Al Sharhan; Laila M Telmesani; Nasser A Aljazan; Bander M Al Qahtani; Mohamed A Lotfy	J Family Community Med	NA	2019	Imam Abdulrahman Bin Faisal University	Observational Studies	Eastern Province	None	None	15 Diseases of the musculoskeletal system or connective tissue	Osteopathies or chondropathies	Osteomyelitis or osteitis
8759534	Facial nerve schwannoma: nerve fibre dissemination.	M Hajjaj; F H Linthicum	J Laryngol Otol	7	1996	Ohud Hospital	Observational Studies	Madinah	NA	None	02 Neoplasms	Neoplasms of central nervous system or related structures	Primary neoplasm of spinal cord, cranial nerves, paraspinal nerves or remaining parts of central nervous system
31452605	The Correlation Between Preoperative Findings of High-Resolution Computed Tomography (HRCT) and Intraoperative Findings of Chronic Otitis Media (COM).	Mariam Aljehani; Rayan Alhussini	Clin Med Insights Ear Nose Throat	8	2019	Ohud Hospital	Observational Studies	Madinah	None	None	10 Ear & mastoid	Diseases of middle ear or mastoid	Otitis media
23461765	Left ear advantages in detecting emotional tones using dichotic listening task	Abdulrahman D Alzahrani; Marwan A Almuhammadi	Laterality	3	2013	King Abdulaziz University	Experimental studies	Makkah	NA	None	V Supplementary section for functioning assessment	Generic functioning domains	Mental functions

PMID	Title	Authors	Journal/Book	Month	Year	First author institution	Type of Publication	Region	Funding	COI	ICD11 Chapter	ICD11 Block	ICD11 Category
	in an Arabic sample.												
7726378	3-Methylglutaconic aciduria: ten new cases with a possible new phenotype.	A al Aqeel; M Rashed; P T Ozand; J Brismar; G G Gascon; A al Odaib; O Dabbagh	Brain Dev	11	1994	Prince Sultan Military Medical City	Observational Studies	Riyadh	King Faisal Specialist Hospital and Research Center	None	05 Endocrine, nutritional or metabolic diseases	Metabolic disorders	Inborn errors of metabolism
23960809	Incidence of Helicobacter pylori infection and their clarithromycin-resistant strains in otitis media with effusion regarding phenotypic and genotypic studies.	Nahla A Melake; Ghada H Shaker; Magdy A Salama	Saudi Pharm J	10	2012	King Saud University	Observational Studies	Riyadh	King Saud University	None	10 Ear & mastoid	Diseases of middle ear or mastoid	Otitis media
33005281	Perinatal atypical teratoid/rhabdoid tumor involving the deep ear structures and complicated by arterial infarction.	Omar Abdullah Alharbi; Musab Hamoud Almushayqih; Abdulmalik Alqahtani; Mohanned Saleh Alsuhaibani	Radiol Case Rep	11	2020	King Faisal Specialist Hospital and Research Centre	Observational Studies	Riyadh	None	None	02 Neoplasms	Neoplasms of central nervous system or related structures	Primary neoplasms of brain

PMID	Title	Authors	Journal/Book	Month	Year	First author institution	Type of Publication	Region	Funding	COI	ICD11 Chapter	ICD11 Block	ICD11 Category
34355054	The Utility of Smartphone 3D Scanning, Open-Sourced Computer-aided Design, and Desktop 3D Printing in the Surgical Planning of Microtia Reconstruction: a Step by Step Guide and Concept Assessment.	Abdualziz Alazzam; Sultan Aljarba; Feras Alshomer; Bassam Alawirdhi	JPRAS Open	12	2021	King Abdulaziz Medical City	Observational Studies	Riyadh	None	None	24 Factors influencing health status or contact with health services	Reasons for contact with the health services	Contact with health services for specific surgical interventions
3378145	Pure word deafness (acquired verbal auditory agnosia) in an Arabic speaking patient.	B A Yaqub; G G Gascon; M Al-Nosha; H Whitaker	Brain	4	1988	King Saud University	Observational Studies	Riyadh	NA	None	10 Ear & mastoid	Disorders with hearing impairment	Acquired hearing impairment
23978857	Intraoperative monitoring of the facial nerve.	Syed A Kamal; Sameer A Al-Bahkaly; Eyas A Othman	Neurosciences (Riyadh)	10	2002	King Abdulaziz Medical City	Observational Studies	Riyadh	NA	None	X Extension Codes	Anatomy and topography	Auditory system
24125535	De novo mutation in the KCNQ1 gene causal to Jervell and Lange-Nielsen syndrome.	J Y Al-Aama; S Al-Ghamdi; A Y Bdier; A A M Wilde; Zahurul A Bhuiyan	Clin Genet	11	2014	King Abdulaziz University	Observational Studies	Makkah	King Abdulaziz City for Science and Technology	None	11 Diseases of the circulatory system	Cardiac arrhythmia	Cardiac arrhythmia associated with genetic disorder

PMID	Title	Authors	Journal/Book	Month	Year	First author institution	Type of Publication	Region	Funding	COI	ICD11 Chapter	ICD11 Block	ICD11 Category
29038714	Ear diseases and factors associated with ear infections among the elderly attending hospital in Arar city, Northern Saudi Arabia.	Naif Gharbi Alenezi; Abdulmajeed Ahmed Alenazi; Yahia Abdelgawad Elsayed Elboraei; Abdulrhman Ahmed Alenazi; Tariq Hulayyil Alanazi; Anwar Khalid Alruwaili; Abdulrahman Sulaiman Alanazi; Bader Khalid Alruwaili; Abdulelah Farhan Alanazi	Electron Physician	9	2017	Northern Border University Arar	Observational Studies	Northern Borders	NA	None	10 Ear & mastoid	Disorders with hearing impairment	Hearing impairment (unspecified)
39981014	Beyond the Brain-Vogt-Koyanagi-Harada Syndrome (VKH): A Rare Brain Eye and Ear (BEE) Syndrome Presenting initially as Aseptic Meningitis- a Neurologist Perspective.	Erum Shariff; Asma Khalaf Alzuabi	Ethiop J Health Sci	7	2024	Imam Abdulrahman Bin Faisal University	Observational Studies	Eastern Province	None	None	14 Diseases of the skin	Skin disorders involving specific cutaneous structures	Disorders of skin colour

PMID	Title	Authors	Journal/Book	Month	Year	First author institution	Type of Publication	Region	Funding	COI	ICD11 Chapter	ICD11 Block	ICD11 Category
28580161	Early onset of Fazio-Londe syndrome: the first case report from the Arabian Peninsula.	Mohammad Arif Hossain; Abdulrahman Obaid; Mohammad Rifai; Hala Alem; Tarek Hazwani; Ali Al Shehri; Majid Alfadhel; Yoshikatsu Eto; Wafaa Eyaid	Hum Genome Var	5	2017	King Abdulaziz Medical City	Observational Studies	Riyadh	Ministry of Health	None	08 Diseases of the nervous system	Motor neuron diseases or related disorders	Motor neuron disease
30891482	A novel homozygous mutation of GJB2-A new variant of keratitis-ichthyosis-deafness syndrome?	Issam Hamadah; Mansoor Haider; Muzamil Chisti	JAAD Case Rep	3	2019	King Faisal Specialist Hospital and Research Centre	Observational Studies	Riyadh	None	None	20 Developmental anomalies	Multiple developmental anomalies or syndromes	Syndromes with skin or mucosal anomalies as a major feature
11573121	Ear, nose and throat manifestations of relapsing polychondritis in a child.	F A Fadl; K M Hassan; N M Oyetunji; S A Al-Nour	Saudi Med J	8	2001	King Fahad Specialist Hospital (Buraydah)	Observational Studies	Qassim	NA	None	15 Diseases of the musculoskeletal system or connective tissue	Osteopathies or chondropathies	Chondropathies
1721798	Intracranial haemorrhages after Nebo hierochonticus scorpion sting.	S H Annobil; M F Omojola; E Vijayakumar	Ann Trop Paediatr	NA	1991	King Saud University	Observational Studies	Riyadh	None	None	22 Injury, poisoning or certain other consequences of external causes	Harmful effects of substances	Harmful effects of or exposure to noxious substances, chiefly nonmedicinal as to source, not elsewhere classified
24027747	A Saudi Patient with an Interstitial Deletion of Short Arm of Chromosome	Ali Y Mersal; Mahaboob K Basha; Zaina S Brinji; Ghazal Avand	J Clin Neonatol	1	2013	King Faisal Specialist Hospital and	Observational Studies	Makkah	None	None	20 Developmental anomalies	Multiple developmental anomalies or syndromes	Syndromes with central nervous system anomalies as

PMID	Title	Authors	Journal/Book	Month	Year	First author institution	Type of Publication	Region	Funding	COI	ICD11 Chapter	ICD11 Block	ICD11 Category
	3 (p13 to p21) and its Association with Joubert's Syndrome Features.					Research Centre							a major feature
25386258	Becker nevus on the neck with ear involvement.	Khalid Mohammad Al Aboud	Dermatol reports	1	2011	King Faisal Hospital	Observational Studies	Makkah	NA	None	20 Developmental anomalies	Structural developmental anomalies primarily affecting one body system	Developmental hamartomata of the epidermis and epidermal appendages
2036697	Anaesthetic-induced ventricular tachyarrhythmia in Jervell and Lange-Nielsen syndrome.	Y Adu-Gyamfi; A Said; U M Chowdhary; A Abomelha; S K Sanyal	Can J Anaesth	4	1991	Imam Abdulrahman Bin Faisal University	Observational Studies	Eastern Province	NA	None	11 Diseases of the circulatory system	Cardiac arrhythmia	Cardiac arrhythmia associated with genetic disorder
27632453	Tympanostomy tubes and prophylactic antibiotic ear drops.	S A Al-Ghamdi	Saudi Med J	7	1999	King Saud University	Experimental studies	Riyadh	NA	None	24 Factors influencing health status or contact with health services	Reasons for contact with the health services	Contact with health services for specific surgical interventions
27279517	An eosinophilic variant granulomatosis with polyangiitis involving the dura, bilateral orbits, and mastoids.	Hasan Al-Hakami; Abdurhman S Al-Arfaj; Mohammed Al-Sohaibani; Najma A Khalil	Saudi Med J	6	2016	King Saud University	Observational Studies	Riyadh	None	None	04 Diseases of the immune system	Nonorgan specific systemic autoimmune disorders	Vasculitis
23377306	An unusual case of mastoid osteoma.	Jihan A Al-Maddah; Sami S Ismail	Neurosciences (Riyadh)	1	2004	Prince Sultan Military Medical City	Observational Studies	Riyadh	None	None	02 Neoplasms	Benign neoplasms, except of lymphoid, haematopoietic, central nervous system or related tissues	Benign mesenchymal neoplasms

PMID	Title	Authors	Journal/Book	Month	Year	First author institution	Type of Publication	Region	Funding	COI	ICD11 Chapter	ICD11 Block	ICD11 Category
2001103	A new syndrome of congenital hypoparathyroidism, severe growth failure, and dysmorphic features.	S A Sanjad; N A Sakati; Y K Abu-Osba; R Kaddoura; R D Milner	Arch Dis Child	2	1991	King Faisal Specialist Hospital and Research Centre	Observational Studies	Riyadh	NA	None	20 Developmental anomalies	Structural developmental anomalies primarily affecting one body system	Structural developmental anomalies of the ear
15005702	The dentofacial features of Sanjad-Sakati syndrome: a case report.	M I Al-Malik	Int J Paediatr Dent	3	2004	King Fahd Armed Forces Hospital	Observational Studies	Makkah	None	None	20 Developmental anomalies	Multiple developmental anomalies or syndromes	Syndromes with skeletal anomalies as a major feature
37431447	Molecular Profile and the Effectiveness of Antimicrobials Drugs Against Staphylococcus aureus and Pseudomonas aeruginosa in the Diagnostic Approaches of Otitis Infection.	Mohammed S Almuhayawi; Hattan S Gattan; Mohammed H Alruhaili; Mohanned Talal Alharbi; Mohammed K Nagshabandi; Muyassar K Tarabulsi; Saad M Almuhayawi; Soad K Al Jaouni; Samy Selim; Awadh Alanazi; Yasir Alruwaili; Osama Ahmed Faried; Islam Amin; Mohamed E Elnosary	Infect Drug Resist	7	2023	King Abdulaziz University	Observational Studies	Makkah	Ministry of Education | King Abdulaziz University	None	10 Ear & mastoid	Diseases of middle ear or mastoid	Otitis media

PMID	Title	Authors	Journal/Book	Month	Year	First author institution	Type of Publication	Region	Funding	COI	ICD11 Chapter	ICD11 Block	ICD11 Category
17663471	Four siblings with distal renal tubular acidosis and nephrocalcinosis, neurobehavioral impairment, short stature, and distinctive facial appearance: a possible new autosomal recessive syndrome.	Eissa Faqeih; Samhar I Al-Akash; Nadia Sakati; Prof Ahmad S Teebi	Am J Med Genet A	9	2007	King Fahad Medical City	Observational Studies	Riyadh	None	None	20 Developmental anomalies	Structural developmental anomalies primarily affecting one body system	Structural developmental anomalies of the ear
29942180	Severe retinal degeneration at an early age in Usher syndrome type 1B associated with homozygous splice site mutations in MYO7A gene.	Ehab Abdelkader; Lama Enani; Patrik Schatz; Leen Safieh	Saudi J Ophthalmol	10	2018	King Khaled Eye Specialist Hospital	Observational Studies	Riyadh	None	None	20 Developmental anomalies	Multiple developmental anomalies or syndromes	Syndromic genetic deafness
33855150	Multiscale sterilizable 3D printed auricular templates to guide cartilaginous framework sizing and sculpture during autologous microtia reconstruction.	Bushra Alhazmi; Feras Alshomer; Bassam Alawirdhi	JPRAS Open	6	2021	King Abdulaziz Medical City	Observational Studies	Riyadh	None	None	24 Factors influencing health status or contact with health services	Reasons for contact with the health services	Contact with health services for specific surgical interventions

PMID	Title	Authors	Journal/Book	Month	Year	First author institution	Type of Publication	Region	Funding	COI	ICD11 Chapter	ICD11 Block	ICD11 Category
38882757	Knowledge and Attitude Toward Otitis Externa: A Saudi Arabian Perspective.	Naif A Albulayhid; Khalid H Al-Jabr; Mohammed M Almazroua; Mubarak H Aldossari; Ibrahim A Alsannat; Abdulwhab M Alotaibi; Yazeed F Almalki; Faihan T Alotaibi; Faris A Alselmi; Mohammed H Karrar Alsharif	J Pharm Bioallied Sci	4	2024	Prince Sattam bin Abdulaziz University	Observational Studies	Riyadh	None	None	10 Ear & mastoid	Diseases of External Ear	Noninfectious inflammation of external ear
37761534	Parents' Knowledge of and Attitude toward Acute Otitis Media and Its Treatment in Children: A Survey at Primary Healthcare Centers in the Aseer Region.	Hayfa Abdulkhaleq AlHefdhi; Maraam Mohammed Al Qout; Alhanouf Yahya Alqahtani; Meshal Mohammed Alqahtani; Roaa Mohammed Asiri; Omair Mohammed Alshahrani; Hanan Delem Almoghamer; Naglaa Youssef; Ramy Mohamed Ghazy	Children (Basel)	9	2023	King Khalid University	Observational Studies	Asir	None	None	10 Ear & mastoid	Diseases of middle ear or mastoid	Otitis media

PMID	Title	Authors	Journal/Book	Month	Year	First author institution	Type of Publication	Region	Funding	COI	ICD11 Chapter	ICD11 Block	ICD11 Category
32251975	Imaging features of mucopolysaccharidoses in the head and neck.	Manal Nicolas-Jilwan	Int J Pediatr Otorhinolaryngol	7	2020	King Faisal Specialist Hospital and Research Centre	Observational Studies	Riyadh	None	None	05 Endocrine, nutritional or metabolic diseases	Metabolic disorders	Inborn errors of metabolism
29408517	Clinical features of LONP1-related infantile cataract.	Arif O Khan; Amani AlBakri	J AAPOS	6	2018	King Faisal Specialist Hospital and Research Centre	Observational Studies	Riyadh	NA	None	20 Developmental anomalies	Structural developmental anomalies primarily affecting one body system	Structural developmental anomalies of the ear
34527879	CHARGE syndrome: A case report of two new CDH7 gene mutations.	Alberto Galvez-Ruiz; Alicia Galindo-Ferreiro; Anthony J Lehner	Saudi J Ophthalmol	7	2020	King Khaled Eye Specialist Hospital	Observational Studies	Riyadh	None	None	05 Endocrine, nutritional or metabolic diseases	Endocrine diseases	Disorders of the pituitary hormone system
38440639	Risk Factors for Chronic and Recurrent Otitis Media in Children: A Review Article.	Kholood Assiri; Jibril Hudise; Ali Obeid	Indian J Otolaryngol Head Neck Surg	2	2024	King Faisal Medical City for Southern Regions	Systematic review and Meta-Analysis	Asir	NA	None	10 Ear & mastoid	Diseases of middle ear or mastoid	Otitis media
34611010	Cochlear implantation in post-lingual adults: A 25-year experience at King Abdullah Ear Specialist Center,	Isra A Aljazeeri; Abdulrahman Alomar; Fatimah AlTassan; Jawaher Alkhayyal; Abdulrahman Alsanosi	Saudi Med J	10	2021	King Saud University	Observational Studies	Riyadh	None	None	24 Factors influencing health status or contact with health services	Reasons for contact with the health services	Presence of device, implants or grafts

PMID	Title	Authors	Journal/Book	Month	Year	First author institution	Type of Publication	Region	Funding	COI	ICD11 Chapter	ICD11 Block	ICD11 Category
	Riyadh, Saudi Arabia.												
39239147	Nasopharyngeal and temporal bone abscess following necrotizing otitis externa: a case report.	Ahmad S Alharthi; Zohour A Almalki; Johara A Alnafie; Hazem K Althobaiti; Mohamed M Ibrahim	J Surg Case Rep	9	2024	Al-Hada Armed Foces Hospital	Observational Studies	Makkah	None	None	10 Ear & mastoid	Diseases of External Ear	Infectious diseases of external ear
35712297	Convolutional Neural Network Based Real Time Arabic Speech Recognition to Arabic Braille for Hearing and Visually Impaired.	Surbhi Bhatia; Ajantha Devi; Razan Ibrahim Alsuwailem; Arwa Mashat	Front Public Health	5	2022	King Faisal University	Experimental studies	Eastern Province	King Faisal University	None	10 Ear & mastoid	Disorders with hearing impairment	Hearing impairment (unspecified)
35282282	Perception, knowledge, and attitude of medical doctors in Saudi Arabia about the role of physiotherapists in vestibular rehabilitation: a cross-sectional survey.	Danah Alyahya; Faizan Z Kashoo	PeerJ	3	2022	Majmaah University	Observational Studies	Riyadh	Majmaah University	None	21 Symptoms, signs or clinical findings, not elsewhere classified	Symptoms, signs or clinical findings of the nervous system	Symptoms or signs involving the nervous system

PMID	Title	Authors	Journal/Book	Month	Year	First author institution	Type of Publication	Region	Funding	COI	ICD11 Chapter	ICD11 Block	ICD11 Category
31630791	Homozygous Loss-of-Function Mutations in AP1B1, Encoding Beta-1 Subunit of Adaptor-Related Protein Complex 1, Cause MEDNIK-like Syndrome.	Hessa S Alsaif; Mohammad Al-Owain; Martin E Barrios-Llerena; Ghada Gosadi; Yousef Binamer; David Devadason; Jane Ravenscroft; Mohnish Suri; Fowzan S Alkuraya	Am J Hum Genet	11	2019	King Faisal Specialist Hospital and Research Centre	Observational Studies	Riyadh	King Salman Center for Disability Research	None	20 Developmental anomalies	Multiple developmental anomalies or syndromes	Syndromic genetic deafness
37240814	Effectiveness of Epley-Canalith Repositioning Procedure versus Vestibular Rehabilitation Therapy in Diabetic Patients with Posterior Benign Paroxysmal Positional Vertigo: A Randomized Trial.	Mohammad Abu Shaphe; Mohammed M Alshehri; Ramzi Abdu Alajam; Rashid Ali Beg; Najat Ibrahim A Hamdi; Saravanakumar Nanjan; Vandana Esht; Mohammed A Aljahni; Hashim Ahmed; Ausaf Ahmad; Aafreen; Ashfaque Khan; Abdur Raheem Khan	Life (Basel)	5	2023	Jazan University	Experimental studies	Jizan	Ministry of Education	None	10 Ear & mastoid	Diseases of inner ear	Episodic vestibular syndrome
33817048	Otitis media detection using tympanic membrane	Adi Alhudhaif; Zafer C√∂mert; Kemal Polat	PeerJ Comput Sci	2	2021	Prince Sattam bin Abdulaziz University	Observational Studies	Riyadh	Prince Sattam bin Abdul	None	10 Ear & mastoid	Diseases of middle ear or mastoid	Otitis media

PMID	Title	Authors	Journal/Book	Month	Year	First author institution	Type of Publication	Region	Funding	COI	ICD11 Chapter	ICD11 Block	ICD11 Category
	images with a novel multi-class machine learning algorithm.								aziz University				
37892347	Clinical and Genetic Characterization of Patients with Primary Ciliary Dyskinesia in Southwest Saudi Arabia: A Cross Sectional Study.	Ali Alsuheel Asseri; Ayed A Shati; Ibrahim A Asiri; Reem H Aldosari; Hassan A Al-Amri; Mohammed Alshahrani; Badriah G Al-Asmari; Haleimah Alalkami	Children (Basel)	10	2023	King Khalid University	Observational Studies	Asir	Ministry of Education	None	20 Developmental anomalies	Structural developmental anomalies primarily affecting one body system	Structural developmental anomalies of the respiratory system

PMID	Title	Authors	Journal/Book	Month	Year	First author institution	Type of Publication	Region	Funding	COI	ICD11 Chapter	ICD11 Block	ICD11 Category
26223816	Quality standards for bone conduction implants	Gavilan J, Adunka O, Agrawal S, Atlas M, Baumgartner WD, Brill S, Bruce I, Buchman C, Caversaccio M, De Bodt MT, Dillon M, Godey B, Green K, Gstoettner W, Hagen R, Hagr A, Han D, Kameswaran M, Karltorp E, Kompis M, Kuzovkov V, Lassaletta L, Li Y, Lorens A, Martin J, Manoj M, Mertens G, Mlynski R, Mueller J, O'Driscoll M, Parnes L, Pulibalathingal S, Radeloff A, Raine CH, Rajan G, Rajeswaran R, Schmutzhard J, Skarzynski H, Skarzynski P, Sprinzl G, Staecker H, Stephan K, Sugarova S, Tavora D, Usami S, Yanov Y, Zernotti M,	Acta Otolaryngol	6	2015	King Saud University	Consensus	Riyadh	None	None	24 Factors influencing health status or contact with health services	Reasons for contact with the health services	Presence of device, implants or grafts

PMID	Title	Authors	Journal/Book	Month	Year	First author institution	Type of Publication	Region	Funding	COI	ICD11 Chapter	ICD11 Block	ICD11 Category
		Zorowka P, de Heyning PV.											

PMID	Title	Authors	Journal/Book	Month	Year	First author institution	Type of Publication	Region	Funding	COI	ICD11 Chapter	ICD11 Block	ICD11 Category
24898070	Acute mastoiditis in children with cochlear implants: is explantation required?	Zawawi F, Cardona I, Akinpelu OV, Daniel SJ.	Otolaryngol Head Neck Surg	6	2014	King Abdulaziz University	Systematic review and Meta-Analysis	Makkah	None	None	24 Factors influencing health status or contact with health services	Reasons for contact with the health services	Presence of device, implants or grafts
23210007	Assessment of noise levels in 200 Mosques in Riyadh, Saudi Arabia	Al Shimemeri SA, Patel CB, Abdulrahman AF.	Avicenna J Med	10	2011	King Saud University	Observational Studies	Riyadh	None	None	10 Ear & mastoid	Diseases of inner ear	Noise effects on inner ear
18946585	Meningoencephalocele presenting as a mass at the external auditory meatus	Jamjoom BA, Sharab M, Jamjoom AB.	Saudi Med J	10	2008	National Guard	Observational Studies	Makkah	None	None	20 Developmental anomalies	Structural developmental anomalies primarily affecting one body system	Structural developmental anomalies of the ear
17684424	Unusual presentation of internal carotid artery aneurysm in the middle ear	Muhayawi SM, Tawfik HA.	Ann Saudi Med	7	2007	King Abdulaziz University	Observational Studies	Makkah	None	None	10 Ear & mastoid	Diseases of middle ear or mastoid	Other specified diseases of middle ear or mastoid
25338707	Overlap between CHARGE and Kabuki syndromes: more than an interesting clinical observation?	Patel N, Alkuraya FS.	Am J Med Genet A	1	2015	King Faisal Specialist Hospital and Research Centre	Observational Studies	Riyadh	None	None	05 Endocrine, nutritional or metabolic diseases	Endocrine diseases	Disorders of the pituitary hormone system
24246099	Lipoma of the middle ear: an unusual presentation in a 6 year old child	Aldosari B.	Acta Otorrinolaringol Esp	11	2014	King Saud University	Observational Studies	Riyadh	None	None	02 Neoplasms	Benign neoplasms, except of lymphoid, haematopoietic, central nervous system or related tissues	Benign mesenchymal neoplasms

PMID	Title	Authors	Journal/Book	Month	Year	First author institution	Type of Publication	Region	Funding	COI	ICD11 Chapter	ICD11 Block	ICD11 Category
18553515	Expanding the "E" in CHARGE	Alazami AM, Alzahrani F, Alkuraya FS.	Am J Med Genet A	7	2008	King Faisal Specialist Hospital and Research Centre	Observational Studies	Riyadh	None	None	05 Endocrine, nutritional or metabolic diseases	Endocrine diseases	Disorders of the pituitary hormone system
28333782	The Normal Adult Human Internal Auditory Canal: A Volumetric Multidetector Computed Tomography Study	Essbaiheen F, Hegazi T, Rosenbloom L.	Otol Neurotol	7	2017	King Saud University	Observational Studies	Riyadh	None	None	X Extension Codes	Anatomy and topography	Auditory system
34719516	Recognition and Comprehension of Multiple-Meaning Words: Examining a Vocabulary Intervention With Hard of Hearing Students	Alasim KN.	Am Ann Deaf	10	2021	Prince Sattam bin Abdulaziz University	Experimental studies	Riyadh	None	None	10 Ear & mastoid	Disorders with hearing impairment	Hearing impairment (unspecified)
32533172	The Effects of a Vocabulary Intervention on Teaching Multiple-Meaning Words to Students Who Are d/Deaf and Hard of Hearing	Alqraini FM, Paul PV.	J Deaf Stud Deaf Educ	9	2020	Prince Sattam bin Abdulaziz University	Experimental studies	Riyadh	None	None	10 Ear & mastoid	Disorders with hearing impairment	Hearing impairment (unspecified)

PMID	Title	Authors	Journal/Book	Month	Year	First author institution	Type of Publication	Region	Funding	COI	ICD11 Chapter	ICD11 Block	ICD11 Category
8787257	Comparison of total intravenous, balanced inhalational and combined intravenous-inhalational anaesthesia for tympanoplasty, septorhinoplasty and adenotonsillectomy	Van den Berg AA, Savva D, Honjol NM, Prabhu NV.	Anaesth Intensive Care	10	1995	Armed Forces Hospital	Experimental studies	Riyadh	None	none	24 Factors influencing health status or contact with health services	Reasons for contact with the health services	Contact with health services for specific surgical interventions
17587954	Tympanoplasty: Factors influencing surgical outcome	Al-Ghamdi SA.	Ann Saudi Med	11	1994	King Saud University	Observational Studies	Asir	None	none	24 Factors influencing health status or contact with health services	Reasons for contact with the health services	Contact with health services for specific surgical interventions
6822769	Hearing levels of school children in Dammam	Ashoor A.	J Laryngol Otol	1	1983	King Faisal University	Observational Studies	Eastern Province	None	none	10 Ear & mastoid	Disorders with hearing impairment	Hearing impairment (unspecified)
20573147	Great auricular nerve blockade using high resolution ultrasound: a volunteer study	Thallaj A, Marhofer P, Moriggl B, Delvi BM, Kettner SC, Almajed M.	Anaesthesia	8	2010	King Saud University	Observational Studies	Riyadh	King Saud University	none	24 Factors influencing health status or contact with health services	Reasons for contact with the health services	Contact with health services for nonsurgical interventions not involving devices

Data File S2. Scimago Journal Rank (SJR) scores and quartile classifications for included journals

Journal	Year	Q	SJR rank	Journal	Year	Q	SJR rank
BDJ Open	2016	Q3	0.019	Int J Pediatr	2021	Q2	0.409
J Multidiscip Healthc	2019	Q1	0.0912	J Med Life	2023	Q3	0.417
Neurosciences (Riyadh)	2002	Q4	0.1	J Otol	2024	Q3	0.417
Otolaryngol Pol	1994	Q4	0.103	J Otol	2024	Q3	0.417
Otolaryngol Pol	1996	Q4	0.103	J Craniofac Surg	2023	Q3	0.42
Neurosciences (Riyadh)	2006	Q4	0.107	Saudi Med J	2024	Q3	0.42
Indian J Otolaryngol Head Neck Surg	2011	Q4	0.107	S Afr J Commun Disord	2019	Q3	0.428
J Laryngol Otol	1996	Q3	0.11	J Int Adv Otol	2024	Q3	0.429
Neurosciences (Riyadh)	2004	Q4	0.124	J Int Adv Otol	2024	Q3	0.429
Sultan Qaboos Univ Med J	2010	Q4	0.125	Saudi Med J	2020	Q3	0.437
Ann Saudi Med	2004	Q3	0.126	Saudi Med J	2020	Q3	0.437
Sultan Qaboos Univ Med J	2011	Q4	0.128	Saudi Med J	2020	Q3	0.437
Dermatol Reports	2011	Q4	0.13	Saudi Med J	2020	Q3	0.437
Neurosciences (Riyadh)	2009	Q4	0.131	J Craniofac Surg	2024	Q2	0.437
Int Med Case Rep J	2014	Q4	0.133	JAAD Case Rep	2019	Q3	0.445
Neurosciences (Riyadh)	2012	Q4	0.138	Acta Inform Med	2022	Q3	0.446
Neurosciences (Riyadh)	2012	Q4	0.138	Ethiop J Health Sci	2024	Q3	0.447
J Clin Neonatol	2013	Q4	0.139	ScientificWorldJournal	2020	Q3	0.453
Saudi J Med Med Sci	2018	Q4	0.13	S Afr J Commun Disord	2024	Q1	0.461
Indian J Otolaryngol Head Neck Surg	2012	Q4	0.153	Medicine (Baltimore)	2024	Q3	0.469
Saudi Med J	1999	Q2	0.153	Int J Neonatal Screen	2020	Q2	0.482
Saudi Med J	2000	Q2	0.155	Neuroradiol J	2018	Q3	0.484
Saudi Med J	2000	Q2	0.155	Neuroradiol J	2020	Q3	0.49
Saudi Med J	2000	Q2	0.155	J Otol	2023	Q2	0.49
Saudi Med J	2000	Q2	0.155	Acta Otolaryngol	2021	Q2	0.493
Saudi Med J	2000	Q2	0.155	Eur Ann Otorhinolaryngol Head Neck Dis	2023	Q2	0.51
Saudi Med J	2001	Q2	0.157	Ann Hum Genet	2024	Q3	0.513
West Afr J Med	2003	Q3	0.16	J Int Adv Otol	2020	Q3	0.518
West Afr J Med	2003	Q3	0.16	Int J Pediatr Otorhinolaryngol	2024	Q2	0.52
Saudi Med J	2005	Q3	0.173	Int J Pediatr Otorhinolaryngol	2024	Q2	0.52
Saudi Med J	2002	Q2	0.178	Int J Pediatr Otorhinolaryngol	2024	Q2	0.52
Saudi Med J	2002	Q2	0.178	Int J Pediatr Otorhinolaryngol	2024	Q2	0.52
Saudi Med J	2002	Q2	0.178	Assist Technol	2023	Q2	0.522
Ann Saudi Med	1997	Q2	0.191	Audiol Res	2024	Q2	0.542
Ann Saudi Med	1997	Q2	0.191	Children (Basel)	2023	Q2	0.545
Ann Saudi Med	1996	Q2	0.191	Children (Basel)	2023	Q2	0.545
Ann Saudi Med	1996	Q2	0.191	J Laryngol Otol	2023	Q2	0.55
Ann Saudi Med	1996	Q2	0.191	Audiol Neurootol	2022	Q2	0.554

Ann Saudi Med	1992	Q2	0.191	Audiol Neurootol	2022	Q2	0.554
Ann Saudi Med	1995	Q2	0.191	Int J Pediatr Otorhinolaryngol	2022	Q2	0.554
Ann Saudi Med	1993	Q2	0.191	Int J Pediatr Otorhinolaryngol	2022	Q2	0.554
J Coll Physicians Surg Pak	2011	Q3	0.197	Int J Pediatr Otorhinolaryngol	2022	Q2	0.554
East Mediterr Health J	1999	Q2	0.197	J Family Community Med	2021	Q2	0.559
Saudi Med J	2008	Q3	0.198	Acta Otorhinolaryngol Ital	2022	Q2	0.56
Saudi Med J	2008	Q3	0.198	J Laryngol Otol	2020	Q2	0.561
Saudi Med J	2006	Q3	0.2	J Epidemiol Glob Health	2017	Q3	0.566
Saudi Med J	2010	Q3	0.205	Int J Pediatr Otorhinolaryngol	2023	Q2	0.568
Saudi Med J	2007	Q3	0.208	Int J Pediatr Otorhinolaryngol	2021	Q2	0.579
Saudi Med J	2003	Q2	0.213	Int J Pediatr Otorhinolaryngol	2021	Q2	0.579
Saudi Med J	2003	Q2	0.213	Int J Pediatr Otorhinolaryngol	2021	Q2	0.579
Saudi Med J	2003	Q2	0.213	Eur Rev Med Pharmacol Sci	2023	Q2	0.58
Saudi Med J	2003	Q2	0.213	Med Sci Monit	2021	Q2	0.601
Pan Afr Med J	2014	Q3	0.221	Plast Reconstr Surg Glob Open	2021	Q2	0.608
Saudi Med J	2011	Q3	0.232	PeerJ Comput Sci	2021	Q2	0.612
Saudi Med J	2011	Q3	0.232	Cochlear Implants Int	2024	Q2	0.616
Saudi Med J	2004	Q2	0.235	Cochlear Implants Int	2024	Q2	0.616
Interv Med Appl Sci	2015	Q3	0.245	World Neurosurg	2018	Q2	0.631
J Trop Pediatr	1997	Q2	0.246	Int J Pediatr Otorhinolaryngol	2020	Q2	0.631
Adv Otorhinolaryngol	2000	Q3	0.246	Int J Pediatr Otorhinolaryngol	2020	Q2	0.631
Adv Otorhinolaryngol	2000	Q3	0.246	Int J Pediatr Otorhinolaryngol	2020	Q2	0.631
Adv Otorhinolaryngol	2000	Q3	0.246	Int J Pediatr Otorhinolaryngol	2020	Q2	0.631
Minerva Pediatr	2015	Q3	0.261	Laryngoscope Investig Otolaryngol	2024	Q2	0.641
Int J Surg Case Rep	2016	Q3	0.261	Int J Pediatr Otorhinolaryngol	2018	Q2	0.648
Adv Otorhinolaryngol	1997	Q3	0.27	Laryngoscope Investig Otolaryngol	2023	Q2	0.649
Saudi Med J	2013	Q3	0.27	Spectrochim Acta A Mol Biomol Spectrosc	2024	Q2	0.664
Ann Saudi Med	2016	Q3	0.27	Int J Pediatr Otorhinolaryngol	2019	Q2	0.668
Saudi Pharm J	2012	Q3	0.289	J AAPOS	2018	Q2	0.668
J Family Community Med	1994	Q3	0.292	Children (Basel)	2024	Q2	0.674
J Family Community Med	2003	Q3	0.292	Plast Reconstr Surg Glob Open	2023	Q2	0.682
Ann Trop Paediatr	1991	Q2	0.293	Hum Genome Var	2017	Q3	0.692
Saudi J Gastroenterol	2011	Q3	0.302	PeerJ	2022	Q2	0.695
Saudi Med J	2016	Q3	0.306	Laryngoscope Investig Otolaryngol	2022	Q1	0.701
Saudi Med J	2016	Q3	0.306	Acta Otolaryngol	2019	Q2	0.706
Br J Neurosurg	1991	Q2	0.319	Infez Med	2024	Q2	0.711
J Chin Med Assoc	2013	Q3	0.35	Am J Audiol	2019	Q2	0.712
J Otolaryngol	1996	Q2	0.382	Am J Audiol	2019	Q2	0.712
J Otolaryngol	1997	Q2	0.382	Life (Basel)	2023	Q2	0.713
J Otolaryngol	1997	Q2	0.382	J Epilepsy Res	2022	Q2	0.727

Occup Med (Lond)	2001	Q2	0.383	J Pers Med	2023	Q2	0.736
Am J Otolaryngol	1998	Q2	0.39	J Pers Med	2023	Q2	0.736
Am J Otolaryngol	1998	Q2	0.39	J Pers Med	2023	Q3	0.736
Am J Otolaryngol	1999	Q2	0.39	Infect Drug Resist	2023	Q2	0.743
Ann Saudi Med	2014	Q3	0.392	Cochlear Implants Int	2019	Q2	0.746
Ann Saudi Med	2014	Q3	0.392	Plast Reconstr Surg Glob Open	2020	Q2	0.759
J Psychoactive Drugs	2004	Q2	0.405	Int J Pediatr Otorhinolaryngol	2017	Q2	0.783
Ann Occup Hyg	2001	Q2	0.411	Int J Pediatr Otorhinolaryngol	2017	Q2	0.783
J Epidemiol Glob Health	2014	Q4	0.443	Eur Arch Otorhinolaryngol	2018	Q1	0.787
J Otolaryngol	2004	Q2	0.445	Eur Arch Otorhinolaryngol	2018	Q1	0.787
Int J Pediatr Otorhinolaryngol	1995	Q2	0.463	Eur Arch Otorhinolaryngol	2024	Q1	0.787
Int J Pediatr Otorhinolaryngol	1994	Q2	0.463	Gene	2013	Q1	0.976
Int J Pediatr Otorhinolaryngol	1993	Q2	0.463	Eur Arch Otorhinolaryngol	2024	Q1	0.787
Int J Pediatr Otorhinolaryngol	1996	Q2	0.463	Eur Arch Otorhinolaryngol	2024	Q1	0.787
Int J Pediatr Otorhinolaryngol	1996	Q2	0.463	Eur Arch Otorhinolaryngol	2024	Q1	0.787
ORL J Otorhinolaryngol Relat Spec	1996	Q2	0.463	Eur Arch Otorhinolaryngol	2024	Q1	0.787
ORL J Otorhinolaryngol Relat Spec	1996	Q2	0.463	Eur Arch Otorhinolaryngol	2024	Q1	0.787
J Laryngol Otol	2015	Q2	0.464	Eur Arch Otorhinolaryngol	2023	Q1	0.792
Br J Radiol	1997	Q2	0.465	Eur Arch Otorhinolaryngol	2023	Q1	0.792
Genet Test Mol Biomarkers	2010	Q3	0.478	Eur Arch Otorhinolaryngol	2023	Q1	0.792
J Laryngol Otol	2011	Q3	0.482	Eur Arch Otorhinolaryngol	2023	Q1	0.792
J Laryngol Otol	2012	Q2	0.511	Res Dev Disabil	2021	Q2	0.796
J Laryngol Otol	1978	Q2	0.516	J Vestib Res	2023	Q1	0.801
J Laryngol Otol	1992	Q2	0.516	PLoS One	2024	Q1	0.803
J Laryngol Otol	1992	Q2	0.516	PLoS One	2024	Q1	0.803
J Laryngol Otol	1987	Q2	0.516	BMC Pediatr	2020	Q1	0.806
J Surg Case Rep	2019	Q4	0.14	J Otolaryngol Head Neck Surg	2024	Q1	0.809
J Laryngol Otol	1995	Q2	0.516	Audiol Neurootol	2021	Q1	0.82
J Laryngol Otol	1994	Q2	0.516	Int J Environ Res Public Health	2022	Q2	0.828
J Laryngol Otol	1993	Q2	0.516	PLoS One	2023	Q1	0.839
J Laryngol Otol	1997	Q2	0.516	PLoS One	2023	Q1	0.839
J Laryngol Otol	1998	Q2	0.516	Otol Neurotol	2023	Q1	0.849
J Laryngol Otol	1999	Q2	0.516	Int J Audiol	2021	Q1	0.85
Cochlear Implants Int	2010	Q2	0.52	PLoS One	2021	Q1	0.852
J Laryngol Otol	2002	Q2	0.521	J Egypt Public Health Assoc	2023	Q1	0.854
J Laryngol Otol	2002	Q2	0.521	Eur Arch Otorhinolaryngol	2022	Q1	0.857
Int J Paediatr Dent	2004	Q2	0.537	Eur Arch Otorhinolaryngol	2022	Q1	0.857
Brain Dev	1994	Q2	0.54	Eur Arch Otorhinolaryngol	2022	Q1	0.857
Ophthalmic Genet	2012	Q2	0.553	Eur Arch Otorhinolaryngol	2022	Q1	0.857
J Clin Anesth	2016	Q2	0.558	Child Care Health Dev	2018	Q2	0.863

J Laryngol Otol	2009	Q2	0.563	Comput Intell Neurosci	2022	Q1	0.863
J Plast Reconstr Aesthet Surg	2005	Q2	0.573	J Pers Med	2024	Q2	0.863
Laterality	2013	Q2	0.603	Otol Neurotol	2024	Q1	0.866
J Otolaryngol Head Neck Surg	2011	Q2	0.609	Eur Arch Otorhinolaryngol	2019	Q1	0.871
J Otolaryngol Head Neck Surg	2011	Q2	0.609	Sci Rep	2023	Q1	0.874
J Vestib Res	2016	Q2	0.616	Sci Rep	2023	Q1	0.874
Int J Hyg Environ Health	2004	Q1	0.628	Sci Rep	2024	Q1	0.874
Int J Pediatr Otorhinolaryngol	2002	Q1	0.629	Sci Rep	2024	Q1	0.874
Int J Pediatr Otorhinolaryngol	2002	Q1	0.629	Sci Rep	2024	Q1	0.874
Int J Pediatr Otorhinolaryngol	2002	Q1	0.629	Otol Neurotol	2022	Q1	0.887
Clin Otolaryngol Allied Sci	1997	Q1	0.64	Res Dev Disabil	2024	Q2	0.9
J Psychoactive Drugs	2002	Q1	0.658	Clin Exp Otorhinolaryngol	2024	Q1	0.91
Int J Pediatr Otorhinolaryngol	2006	Q2	0.661	J Clin Med	2024	Q1	0.919
ORL J Otorhinolaryngol Relat Spec	2012	Q2	0.665	J Clin Med	2024	Q1	0.919
J Plast Reconstr Aesthet Surg	2007	Q1	0.666	J Clin Med	2024	Q1	0.919
BMC Res Notes	2014	Q2	0.669	J Clin Med	2024	Q1	0.919
Ophthalmic Genet	2013	Q3	0.691	BMC Endocr Disord	2017	Q1	0.925
Cochlear Implants Int	2009	Q2	0.692	Eur Arch Otorhinolaryngol	2021	Q1	0.926
Otolaryngol Head Neck Surg	1994	Q1	0.703	BMC Med Genet	2011	Q3	0.98
Otolaryngol Head Neck Surg	1999	Q1	0.703	Eur Arch Otorhinolaryngol	2021	Q1	0.926
Pharmacotherapy	2002	Q1	0.705	Eur Arch Otorhinolaryngol	2021	Q1	0.926
Int J Pediatr Otorhinolaryngol	2015	Q2	0.707	Am J Otol	1986	Q1	0.992
Int J Pediatr Otorhinolaryngol	2016	Q2	0.707	Int J Audiol	2018	Q1	0.933
Int J Pediatr Otorhinolaryngol	2016	Q2	0.707	Am J Otol	1996	Q1	0.992
Int J Pediatr Otorhinolaryngol	2016	Q2	0.707	Int J Audiol	2024	Q1	0.941
Int J Pediatr Otorhinolaryngol	2005	Q1	0.722	Am J Otol	1996	Q1	0.992
Ann Plast Surg	2004	Q1	0.746	Otol Neurotol	2021	Q1	0.962
Plast Reconstr Surg	1998	Q1	0.749	Biomedicines	2023	Q1	0.962
Eur Arch Otorhinolaryngol	2011	Q2	0.754	Laryngoscope	2011	Q1	0.999
Eur Arch Otorhinolaryngol	2011	Q2	0.754	Ann Plast Surg	2009	Q1	1.061
Am J Med Genet A	2007	Q3	0.759	Mol Vis	2012	Q1	1.068
Int J Pediatr Otorhinolaryngol	2014	Q2	0.774	Front Neurol	2023	Q2	0.966
J Surg Case Rep	2023	Q3	0.18	Int J Audiol	2022	Q1	0.971
Can J Anaesth	1996	Q1	0.783	J Neurol Sci	2015	Q2	1.068
Can J Anaesth	1991	Q1	0.783	Front Neurol	2022	Q2	0.978
Int J Pediatr Otorhinolaryngol	2010	Q1	0.798	BMC Med Genet	2016	Q3	1.088
Eur Arch Otorhinolaryngol	2014	Q2	0.842	Trop Geogr Med	1985	Q1	1.15
Eur J Med Genet	2014	Q3	0.862	Ann Otol Rhinol Laryngol	1996	Q1	1.183
Eur Arch Otorhinolaryngol	2015	Q2	0.863	Ann Otol Rhinol Laryngol	1997	Q1	1.183
J Voice	2012	Q1	0.87	BMJ Open	2021	Q1	0.982

Int J Pediatr Otorhinolaryngol	2012	Q1	0.876	Sci Rep	2021	Q1	1.005
Int J Pediatr Otorhinolaryngol	2012	Q1	0.876	Laryngoscope	1988	Q1	1.222
Arch Dis Child	1991	Q1	0.915	Int J Audiol	2015	Q1	1.236
Clin Radiol	2011	Q1	0.927	Otol Neurotol	2014	Q1	1.26
Eur J Med Genet	2015	Q3	0.928	Am J Med Genet A	2014	Q2	1.278
Int Tinnitus J	2024	Q3	0.185	J Endocr Soc	2019	Q2	1.017
Int Tinnitus J	2023	Q3	0.188	Laryngoscope	2024	Q1	1.029
Int J Surg Case Rep	2022	Q3	0.193	Laryngoscope	2024	Q1	1.029
Am Ann Deaf	2022	Q4	0.199	Am J Trop Med Hyg	2007	Q1	1.393
J Surg Case Rep	2024	Q3	0.199	Otolaryngol Head Neck Surg	2023	Q1	1.078
J Surg Case Rep	2024	Q3	0.199	Otol Neurotol	2012	Q1	1.403
BMJ Case Rep	2019	Q3	0.204	J Neurosurg	2001	Q1	1.482
Eur J Plast Surg	2017	Q3	0.207	AJR Am J Roentgenol	2000	Q1	1.581
Clin Case Rep	2024	Q4	0.21	J Pediatr Genet	2021	Q2	1.089
Clin Case Rep	2024	Q4	0.21	Clin Genet	2010	Q2	1.618
Radiol Case Rep	2020	Q4	0.221	Clin Genet	2014	Q2	1.729
Trop Doct	2019	Q4	0.227	Prenat Diagn	2014	Q2	1.78
Indian J Surg Oncol	2022	Q3	0.227	Laryngoscope	2022	Q1	1.103
Afr J Paediatr Surg	2024	Q3	0.227	Eur J Hum Genet	2009	Q1	2.001
Med Arch	2018	Q3	0.228	J Clin Epidemiol	2015	Q1	2.861
Ann Saudi Med	2019	Q3	0.232	J Med Genet	2012	Q1	3.356
J Comput Tomogr	1987	Q1	0.963	Brain	1988	Q1	3.994
Ann Saudi Med	2019	Q3	0.232	Front Public Health	2022	Q1	1.125
Ann Saudi Med	2019	Q3	0.232	Diabetes Care	2013	Q1	4.67
Int J Surg Case Rep	2020	Q3	0.232	Otol Neurotol	2020	Q1	1.147
Int J Surg Case Rep	2019	Q3	0.233	Laryngoscope	2021	Q1	1.167
Int J Surg Case Rep	2019	Q3	0.233	Laryngoscope	2021	Q1	1.167
BMJ Case Rep	2023	Q4	0.235	Otol Neurotol	2019	Q1	1.169
Am Ann Deaf	2021	Q3	0.237	Clin Interv Aging	2020	Q1	1.184
Ann Saudi Med	2017	Q3	0.238	Otol Neurotol	2017	Q1	1.233
Int J Surg Case Rep	2024	Q3	0.238	Int Immunopharmacol	2024	Q1	1.239
Int J Surg Case Rep	2024	Q3	0.238	Diabetes Metab Res Rev	2020	Q1	1.307
Curr Med Imaging	2021	Q3	0.242	Genes (Basel)	2020	Q2	1.337
Am J Case Rep	2019	Q3	0.251	Otolaryngol Head Neck Surg	2022	Q1	1.348
Indian J Otolaryngol Head Neck Surg	2024	Q3	0.254	Pediatr Infect Dis J	2017	Q1	1.392
J Pharm Bioallied Sci	2023	Q2	0.261	Am J Med Genet B Neuropsychiatr Genet	2020	Q1	1.393
Sultan Qaboos Univ Med J	2019	Q3	0.264	Sci Rep	2017	Q1	1.533
Am J Case Rep	2018	Q3	0.271	Ann Med	2023	Q1	3.337
Am J Case Rep	2022	Q3	0.271	Genet Med	2018	Q1	6.285
Am J Case Rep	2022	Q3	0.271	Am J Hum Genet	2019	Q1	7.376

Niger J Clin Pract	2024	Q3	0.273	Acta Otolaryngol	2015	Q2	0.712
Niger J Clin Pract	2022	Q3	0.277	Otolaryngol Head Neck Surg	2014	Q1	1.176
Am J Case Rep	2023	Q3	0.282	Avicenna J Med	2011		
Indian J Otolaryngol Head Neck Surg	2022	Q3	0.284	Saudi Med J	2008	Q3	0.198
Neurosciences (Riyadh)	2024	Q3	0.285	Ann Saudi Med	2007	Q3	0.184
J Med Imaging Radiat Sci	2022	Q3	0.286	Am J Med Genet A	2015	Q2	1.117
Ear Nose Throat J	2021	Q3	0.287	Acta Otorrinolaringol Esp	2014	Q3	0.298
Ear Nose Throat J	2021	Q3	0.287	Am J Med Genet A	2008	Q2	1.095
Ear Nose Throat J	2021	Q3	0.287	Otol Neurotol	2017	Q1	1.233
Pan Afr Med J	2020	Q3	0.287	Am Ann Deaf	2021	Q3	0.237
Ear Nose Throat J	2021	Q3	0.287	J Deaf Stud Deaf Educ	2020	Q1	0.862
Dermatol Online J	2024	Q3	0.291	Anaesth Intensive Care	1995	Q1	0.452
J Family Community Med	2017	Q3	0.292	Ann Saudi Med	1994	Q2	0.191
J Family Community Med	2019	Q3	0.292	J Laryngol Otol	1983	Q3	0.11
JPRAS Open	2021	Q3	0.306	Anaesthesia	2010	Q1	1.497
JPRAS Open	2021	Q3	0.306	Cureus	2019	NA	NA
Am Ann Deaf	2023	Q3	0.321	Cureus	2019	NA	NA
Indian J Otolaryngol Head Neck Surg	2019	Q3	0.322	Cureus	2020	NA	NA
J Pharm Bioallied Sci	2024	Q2	0.323	Cureus	2020	NA	NA
Saudi Med J	2021	Q3	0.345	Cureus	2021	NA	NA
Saudi Med J	2021	Q3	0.345	Cureus	2021	NA	NA
Saudi Med J	2021	Q3	0.345	Cureus	2021	NA	NA
Saudi Med J	2021	Q3	0.345	Cureus	2022	NA	NA
Saudi Med J	2021	Q3	0.345	Cureus	2022	NA	NA
Ann Saudi Med	2021	Q3	0.354	Cureus	2023	NA	NA
Saudi J Ophthalmol	2020	Q3	0.359	Cureus	2023	NA	NA
Afr J Reprod Health	2021	Q3	0.36	Cureus	2023	NA	NA
Saudi J Ophthalmol	2018	Q3	0.36	Cureus	2023	NA	NA
Saudi Med J	2022	Q3	0.361	Cureus	2023	NA	NA
Saudi Med J	2022	Q3	0.361	Cureus	2023	NA	NA
Saudi Med J	2022	Q3	0.361	Cureus	2023	NA	NA
Ann Saudi Med	2018	Q3	0.364	Cureus	2024	NA	NA
Ear Nose Throat J	2022	Q3	0.366	Cureus	2024	NA	NA
Ear Nose Throat J	2022	Q3	0.366	Cureus	2024	NA	NA
Ear Nose Throat J	2022	Q3	0.366	Cureus	2024	NA	NA
Ear Nose Throat J	2022	Q3	0.366	Cureus	2024	NA	NA
Ear Nose Throat J	2024	Q3	0.369	Cureus	2024	NA	NA
Ear Nose Throat J	2024	Q3	0.369	J Family Med Prim Care	2022	NA	NA
Ear Nose Throat J	2024	Q3	0.369	J Family Med Prim Care	2022	NA	NA
Ear Nose Throat J	2024	Q3	0.369	J Family Med Prim Care	2020	NA	NA

Ear Nose Throat J	2024	Q3	0.369	J Family Med Prim Care	2022	NA	NA
Ear Nose Throat J	2024	Q3	0.369	J Family Med Prim Care	2019	NA	NA
Audiol Res	2022	Q3	0.371	J Family Med Prim Care	2021	NA	NA
Ann Med Surg (Lond)	2021	Q3	0.373	JCEM Case Rep	2022	NA	NA
Ann Saudi Med	2020	Q3	0.373	JPGN Rep	2024	NA	NA
Ann Saudi Med	2020	Q3	0.373	Undersea Biomed Res	1992	NA	NA
Ann Saudi Med	2020	Q3	0.373	Case Rep Otolaryngol	2021	NA	NA
Saudi Med J	2023	Q3	0.379	Case Rep Otolaryngol	2015	NA	NA
Saudi Med J	2019	Q3	0.381	Case Rep Otolaryngol	2017	NA	NA
Saudi Med J	2019	Q3	0.381	Clin Med Insights Ear Nose Throat	2019	NA	NA
Saudi Med J	2019	Q3	0.381	Electron Physician	2017	NA	NA
Ear Nose Throat J	2023	Q3	0.382	Int J Health Sci (Qassim)	2007	NA	NA
J Med Life	2022	Q3	0.386	Int J Health Sci (Qassim)	2007	NA	NA
Ann Med Surg (Lond)	2022	Q3	0.387	Int J Health Sci (Qassim)	2014	NA	NA
J Int Adv Otol	2023	Q3	0.387	Interact J Med Res	2020	NA	NA
J Craniofac Surg	2022	Q3	0.396	Int J Health Sci (Qassim)	2015	NA	NA
J Int Adv Otol	2022	Q3	0.399	Int J Health Sci (Qassim)	2024	NA	NA
